# Cross-Species Insights from Single-Nucleus Sequencing Highlight Aging-Related Hippocampal Features in Tree Shrew

**DOI:** 10.1093/molbev/msaf020

**Published:** 2025-02-28

**Authors:** Liu-Lin Xiong, Rui-Ze Niu, Li Chen, Li-Ren Huangfu, Jing Li, Lu-Lu Xue, Yi-Fei Sun, Li-Mei Wang, Yong-Ping Li, Jia Liu, Ting-Hua Wang

**Affiliations:** Department of Neurosurgery, Institute of Neurological Disease, National-Local Joint Engineering Research Center of Translational Medicine, West China Hospital, Sichuan University, Chengdu 610041, Sichuan, China; Department of Anesthesiology, The First People’s Hospital of Zunyi (The Third Affiliated Hospital of Zunyi Medical University), Zunyi 563000, Guizhou, China; Translational Neuromedicine Laboratory, Affiliated Hospital of Zunyi Medical University, Zunyi 563003, Guizhou, China; Science and Education Department, Mental Health Center of Kunming Medical University, Kunming 650034, Yunnan, China; Department of Neurosurgery, Institute of Neurological Disease, National-Local Joint Engineering Research Center of Translational Medicine, West China Hospital, Sichuan University, Chengdu 610041, Sichuan, China; Institute of Neuroscience, Kunming Medical University, Kunming 650500, Yunnan, China; Institute of Neuroscience, Kunming Medical University, Kunming 650500, Yunnan, China; State Key Lab of Biotherapy, Sichuan University, Chengdu 610041, Sichuan, China; Institute of Neuroscience, Kunming Medical University, Kunming 650500, Yunnan, China; Institute of Neuroscience, Kunming Medical University, Kunming 650500, Yunnan, China; Institute of Neuroscience, Kunming Medical University, Kunming 650500, Yunnan, China; Institute of Neuroscience, Kunming Medical University, Kunming 650500, Yunnan, China; Department of Neurosurgery, Institute of Neurological Disease, National-Local Joint Engineering Research Center of Translational Medicine, West China Hospital, Sichuan University, Chengdu 610041, Sichuan, China

**Keywords:** tree shrew, cross-species, aging, neural stem cells, single-nucleus RNA sequencing

## Abstract

The tree shrew brain has garnered considerable attention due to its remarkable similarities to human brain. However, the cellular composition and genetic signatures of tree shrew hippocampus across postnatal life remain poorly characterized. Here, we establish the first single-nucleus transcriptomic atlas of tree shrew hippocampus spanning postnatal life, detailing the dynamics and diversity of the neurogenic lineage, oligodendrocytes, microglia, and endothelial cells. Notably, cross-species transcriptomic comparison among humans, macaques, tree shrews, and mice reveals that the tree shrew transcriptome resembles that of macaques, making it a promising model for simulating human neurological diseases. More interestingly, we identified a unique class of tree shrew-specific neural stem cells and established *SOX6*, *ADAMTS19*, and *MAP2* as their markers. Furthermore, aberrant gene expression and cellular dysfunction in the tree shrew hippocampus are linked to neuroinflammation and cognitive impairment during tree shrew aging. Our study provides extensive resources on cell composition and transcriptomic profiles, serving as a foundation for future research on neurodevelopmental and neurological disorders in tree shrews.

## Graphical Abstract

**Graphical Abstract:**
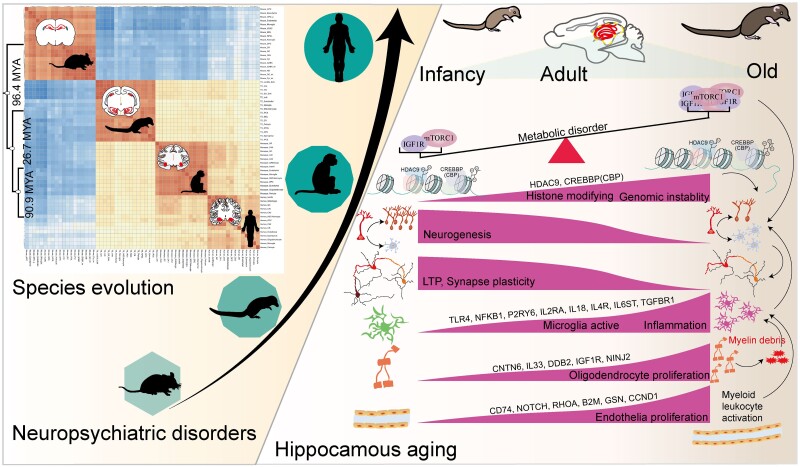

## Introduction

The ethical and practical challenges in acquiring disease-free human brain tissues, particularly the hippocampus, from individuals across different age groups, limit unbiased studies of human hippocampal degeneration. Molecular genetic evidence suggests that humans and tree shrews (TS, *Tupaia belangeri*) shared a common ancestor ∼90.9 million years ago (Fan et al. 2013). Genome sequencing has further confirmed that TS is a close relative of primates and is more closely to humans than rodents (Fan et al. 2019). TS brain exhibits greater anatomical, physiological, and developmental similarities to the human brain compared with other commonly used small laboratory animals (Wang et al. 2019b). Additionally, TS possess several advantages over primates for biomedical research, including smaller body size, shorter breeding cycles, and lower maintenance costs. These features make TS a promising alternative model for studying the evolution, function, and disease pathology of the central nervous system (CNS).

Previous studies have explored the TS genome, transcriptome, epigenome, and radiomics, providing foundational insights into their structure and function (Dai et al. 2017; Huang et al. 2018; Lu et al. 2018). Given their similar genetic, physiological, and neurological characteristics to humans, TS are well suited as models for investigating primate hippocampal aging. However, traditional bulk data approaches are intrinsically restricted in dissecting the heterogeneity and complex microenvironment of tissues. The hippocampus, a highly heterogeneous structure, undergoes significant cellular changes across the lifespan that contribute to cognitive decline (Faye et al. 2018), necessitating single-cell resolution for a comprehensive analysis. Single-cell/single-nucleus RNA sequencing (scRNA-seq/snRNA-seq) techniques have proven invaluable for decoding transcriptional alterations and cellular heterogeneity in the process of aging across various organs (Angelidis et al. 2019; Hao et al. 2022). Despite their potential, the single-cell architecture and functional characteristics of TS remain poorly understood, particularly in the hippocampus. This gap hinders their application in functional and pathological studies, including precise neuron-type classification and intercellular communication analysis.

In this study, we represented the first single-cell resolution resource for the TS hippocampus across postnatal life and cross-species comparative study. We delineated the cellular composition and molecular signatures within the TS hippocampus at single-cell resolution, identifying unique cellular and molecular features. Additionally, we investigated transcriptomic similarities and divergence across humans, macaques, TS, and mice, focusing on age-dependent changes in cellular abundance, cross-species relevance to various nervous system disorders, and specific physiological functions. These analyses highlight the considerable potential of TS serving as an alternative model for CNS studies. Our results uncovered aging-associated transcriptional alterations and hippocampal dysfunctions, offering new insights into the early onset of dysregulation in adult hippocampal neurogenesis and the factors contributing to a hostile microenvironment for neurogenesis in the aging hippocampus.

## Results

### Evolutionary Conservation and Divergence of Hippocampus Across Humans, Macaques, TS, and Mice

**Fig. 1.**
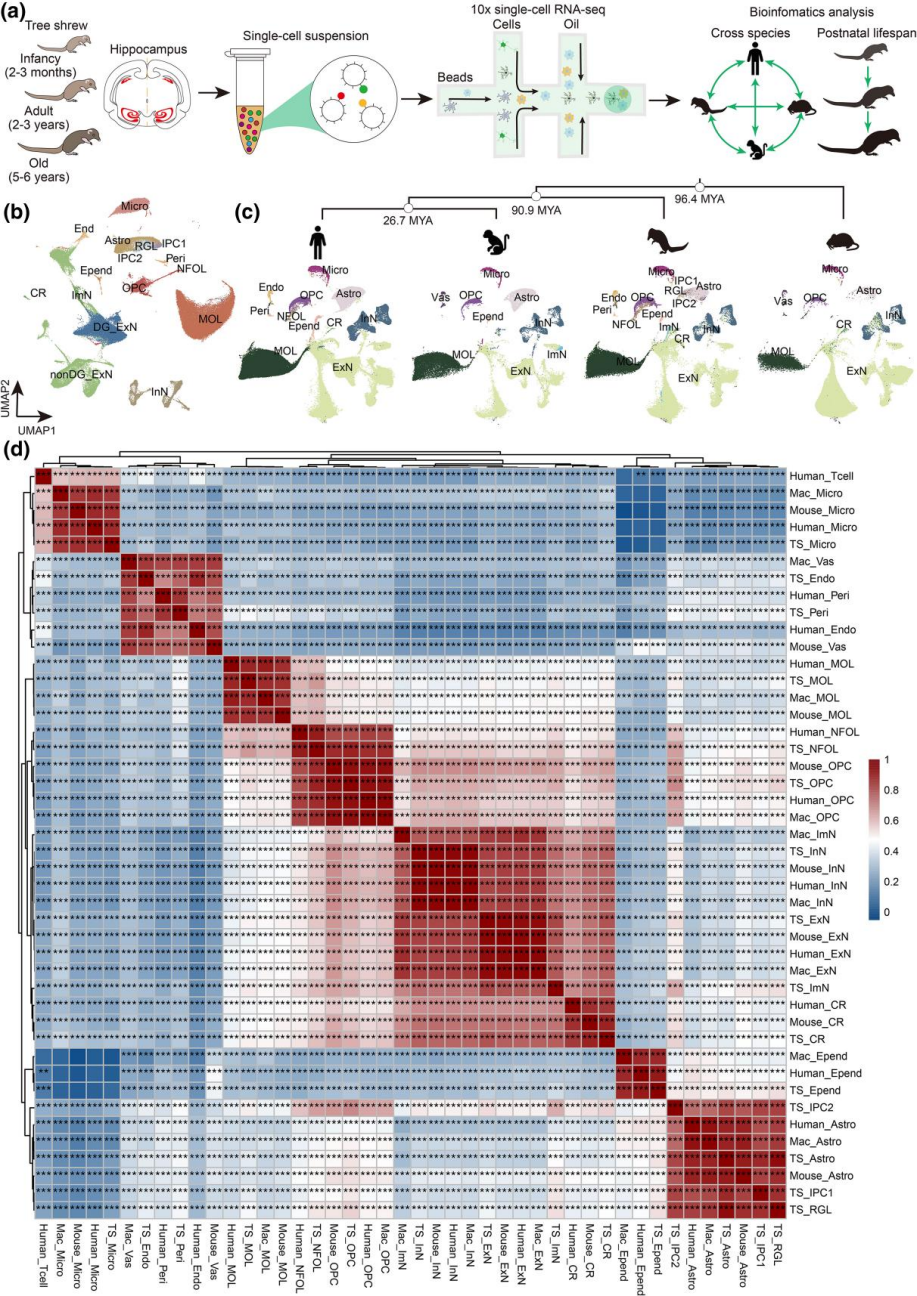
Transcriptomic cell type taxonomy of TS hippocampus and cross-species analysis. a) Flow chart of snRNA-seq and bioinformatics analysis of the TS hippocampus. Infancy, *n* = 5; adult, *n* = 5; old, *n* = 7 TS. b) UMAP plot showing distribution of different cell types in the TS hippocampus. c) Top, a phylogenetic tree of humans, macaques, mice, and TS. Each node represents the timing of evolutionary divergence, with the corresponding time labeled on the right of nodes (MYA: million years ago). Bottom, UMAP plot showing the alignment of hippocampus datasets with cells colored by species. d) Heatmap showing the correlation of cell types across species (Spearman, *P < 0.05, **P < 0.01, ***P < 0.001, significance test for Spearman's rank correlation coefficient using *t*-distribution approximation). RGLs, radial glia-like cells; nIPC, neuronal intermediate progenitor cells; Astro, Astrocytes; CR, Cajal–Retzius cells; DG, dentate gyrus; ExN, excitatory neurons; InN, inhibitory neurons; Astro, astrocytes; Micro, microglia; MOL, mature oligodendrocytes; OPC, oligodendrocyte progenitor cells; Endo, endothelial cells; Peri, pericytes.

Hippocampal cells were collected from infant (2 to 3 months old, *n* = 5, two males and three females), adult (2 to 3 years old, *n* = 5, two males and three females), and old (5 to 6 years old, *n* = 7, four males and three females) TS for snRNA-seq and cross-species comparative analysis (Fig. 1a and supplementary fig. S1a, Supplementary Material online). The resulting dataset included 108,824 high-quality transcriptomes obtained through 10 × Genomics sequencing, passing stringent quality control metrics (Materials and Methods and supplementary fig. S1b and c and supplementary table S1, Supplementary Material online). Unsupervised clustering and uniform manifold approximation and projection (UMAP) visualized 42 transcriptomically distinct cell populations (supplementary fig. S1d and supplementary table S1, Supplementary Material online). These populations cover all major cell categories in the hippocampus (Hochgerner et al. 2018; Zhong et al. 2020; Hao et al. 2022), which comprise (i) neuroglial cells: *VCAN*^+^*OLIG1*^+^ oligodendrocyte progenitor cells (OPCs), *BCAS1*^+^*FYN*^+^ newly formed oligodendrocytes (NFOLs), and *PLP1*^+^*ST18*^+^ mature oligodendrocytes (MOLs); (ii) *SNAP25*^+^ neurons: including *SLC6A1*^+^*GAD1*^+^ inhibitory neurons (InN), *SLC17A7*^+^ excitatory neurons (ExN), *RELN*^+^ Cajal–Retzius cells (CRs), and *PROX1*^+^*DCX*^+^ immature neurons (ImN); (iii) vascular cells: *EBF1*^+^*RGS5*^+^ endothelial (Endo) and *DCN*^+^*COL1A2*^+^ pericytes (Peri); (iv) *CD74*^+^*CSF1R*^+^ microglia (Micro); (v) *DNAH9*^+^*CFAP54*^+^ ependymal; (vi) *SLC1A3*^+^*GFAP*^+^*AQP4*^+^ astrocytes (Astro); and (vii) neuronal progenitor cells (NPCs): *SLC1A3*^+^*SOX2*^+^*ETNPPL*^+^ radial glia-like cells (RGLs), *SLC1A3*^+^*GFAP*^+^*SOX2*^+^*NOTCH2*^high^*WIF1*^high^ neuronal intermediate progenitor cells (IPC1), and *SOX2*^+^*EGFR*^+^*ASCL1*^+^*CDK1*^+^*TOP2A*^+^*NIFK*(*MKI67*)^+^ proliferative IPC (IPC2) **(**Fig. 1b and supplementary fig. S1d and e and supplementary tables S2 and S3, Supplementary Material online). *PROX1*, a transcription factor (TF) critical for dentate gyrus (DG) granule cell genesis (Zhong et al. 2020), was used to subclassify ExN as DG ExN or non-DG ExN (supplementary fig. S1f, Supplementary Material online). The gene set enrichment analysis (GSEA) conducted based on cellular markers suggests that the predominant cell types present in the hippocampus of the TS align with previously identified functional characteristics (supplementary tables S4 and S5, Supplementary Material online). Moreover, we constructed a regulatory network of TFs, which defined core TFs unique to each cell type (supplementary table S6, Supplementary Material online). Of these, the prominent genes comprising the cell-type-specific TFs network included *ETV6* for Micro, *THRA* for MOL, and *DLX2* for InN. The cell types most regulated by TFs are Micro and endothelial cells, which is consistent with observations in the macaque hippocampus (Zhang et al. 2021). Meanwhile, *ZMAT4* was shared by ImN, InN, and ExN, suggesting its essential roles in neuron specification and functional maintenance (supplementary table S6, Supplementary Material online**)**. The network depicted unique and coordinated transcriptional regulation for the establishment of hippocampal cell identities. Together, this comprehensive cellular atlas enables detailed delineation of age-related molecular and cellular alterations in the TS hippocampus.

To explore hippocampal conservation across species, we integrated hippocampal datasets from humans, macaques, mice, and TS (Fig. 1c and supplementary table S7, Supplementary Material online). A robust filtering strategy tailored for cross-species analysis before integration ensured consistency and comparability (Materials and Methods and supplementary fig. S2, Supplementary Material online). Transcriptomes were aligned to their respective reference genomes using STAR and normalized with Seurat's SCTransform. Orthologous genes were identified through homologene and biomaRt, retaining one-to-one orthologs based on human annotations for reliable functional analysis. Species-specific cellular composition revealed major cell types conserved across these species (supplementary fig. S3, Supplementary Material online). Spearman’s correlation analysis demonstrated higher transcriptomic similarity of TS hippocampal cells with macaques and humans than with mice (Fig. 1d and supplementary table S8, Supplementary Material online). Additionally, we used TooManycells, a graph-based algorithm suite for efficient and unbiased identification and visualization of cell clades (Schwartz et al. 2020), to visualize transcriptionally similar hippocampal cells across species. It maintains and presents clustering relationships across different clustering resolutions, accurately identifying and clearly displaying both rare subpopulations and abundant ones. To minimize discrepancies arising from cell annotations, we conducted our analysis using the original annotation data from the referenced literature. This method employs hierarchical clustering to group cell populations based on their gene expression profiles. The resulting tree structure illustrates the degree of similarity or dissimilarity between cell populations, with branches length indicating the level of dissimilarity. Cells with more similar gene expression profiles are grouped together, resulting in shorter branches. TooManycells results revealed higher clumpiness (quantifying the degree of “clumped” or colocalized for different cell populations) between TS and humans than that observed between humans and either macaques or mice (supplementary fig. S4a, Supplementary Material online). Additional stricter analytical criteria revealed that TS ExN are more closely related to those in humans and macaques (supplementary fig. S4b, Supplementary Material online). The cell-based TooManyCells tree further illustrated the heterogeneity and consistency of hippocampal cells among different species. The heatmap of the cross-species taxonomy matrix (supplementary fig. S4c, Supplementary Material online) displayed that most TS hippocampal cell types aligned closely with their counterparts in other species, indicating that hippocampal cells are highly conserved across primates, Scandentia, and rodents. Interestingly, some TS cell types, such as DG_ExN, nonDG_ExN, InN, OPC, oligodendrocytes, and endothelial cells, were more closely aligned with primates than with other rodents, whereas certain TS cell types, such as CR and Astro, exhibited closer similarities to those in mice than to those in macaques (supplementary fig. S4d, Supplementary Material online). This analysis highlights the evolutionary conservation and divergence of hippocampal cellular architecture, providing new insights into primate and rodent models of hippocampal biology.

Intercellular communication in the hippocampus plays a vital role in the structure and function of maintaining normal tissue development and homeostasis. We then performed a comparison with corresponding snRNA-seq datasets of the humans, macaques, mice, and TS to gain a better understanding of the cell–cell interactions across these four species (supplementary fig. S5a and b and supplementary table S9, Supplementary Material online). Notably, we identified several species-specific signaling pathways (supplementary fig. S5c, Supplementary Material online): human-specific pathways (*PARs, GALECTIN, IL16, CHEMERIN*), macaque-specific pathways (*AVP* and *CCK*), TS-specific pathways (*NTS*, *CCL6, IL6, RESISTIN*, and *SOMATOSTAIN*), and mouse-specific pathways (*CXCL, MIF, ENHO, VIP, NPY,* and *ACTIVIN*). In addition to these species-specific pathways, we observed many evolutionarily conserved signaling pathways such as *FGF*, *VEGF*, *EGF*, *NRG*, *PTN*, and *IGF,* which are associated with nerve formation, stem cell maintenance, and immune responses. Nevertheless, these conserved signaling pathways vary in their degree of interaction and cellular connections across different species. For instance, *EGF* is central to expansion of neural stem cells (NSCs) and neuroblasts (NB) and promotes, particularly in neurogenesis in the olfactory bulb and DG regions (Gómez-Gaviro et al. 2012). *PTN* is a neurotrophic factor that participates in regulating embryonic CNS development and NSC functions (García-Jiménez et al. 2022). Across all species, *EGF* and *PTN* emerged as prominent communication signals among NSCs, Astro, neurons, and oligodendrocytes, highlighting their importance in neurogenesis (supplementary fig. S5d, Supplementary Material online). Insulin and *IGF* signaling, which function in stem-cell homeostasis across species, also play critical roles in NSCs self-renewal, neurogenesis, cognition, and sensory functions (Ziegler et al. 2015). A recent study has indicated that *GABA* stimulates *IGF-1* release via *GABAA* and *GABAB* receptors, leading to growth promotion performance via *IGF1R* (Athapaththu et al. 2021). Particularly, among TS, macaques, and humans, there is a stronger communication between InN, RGLs, Astro, and oligodendrocytes through the *IGF* pathway (supplementary fig. S5d–f, Supplementary Material online), highlighting the conserved mechanisms involved in growth and neuroprotection.

These findings underscore the conservation of key signaling pathways across species and their relevance to neurodevelopment and immune regulation, with species-specific differences contributing to unique biological features. The identification of these pathways provides important insights into species-specific neurobiology and disease mechanisms, especially in the context of neurodegenerative and neurodevelopmental diseases.

### Cross-Species Comparison of Disease-Associated Gene Enrichment in Hippocampal Cell Types

**Fig. 2.**
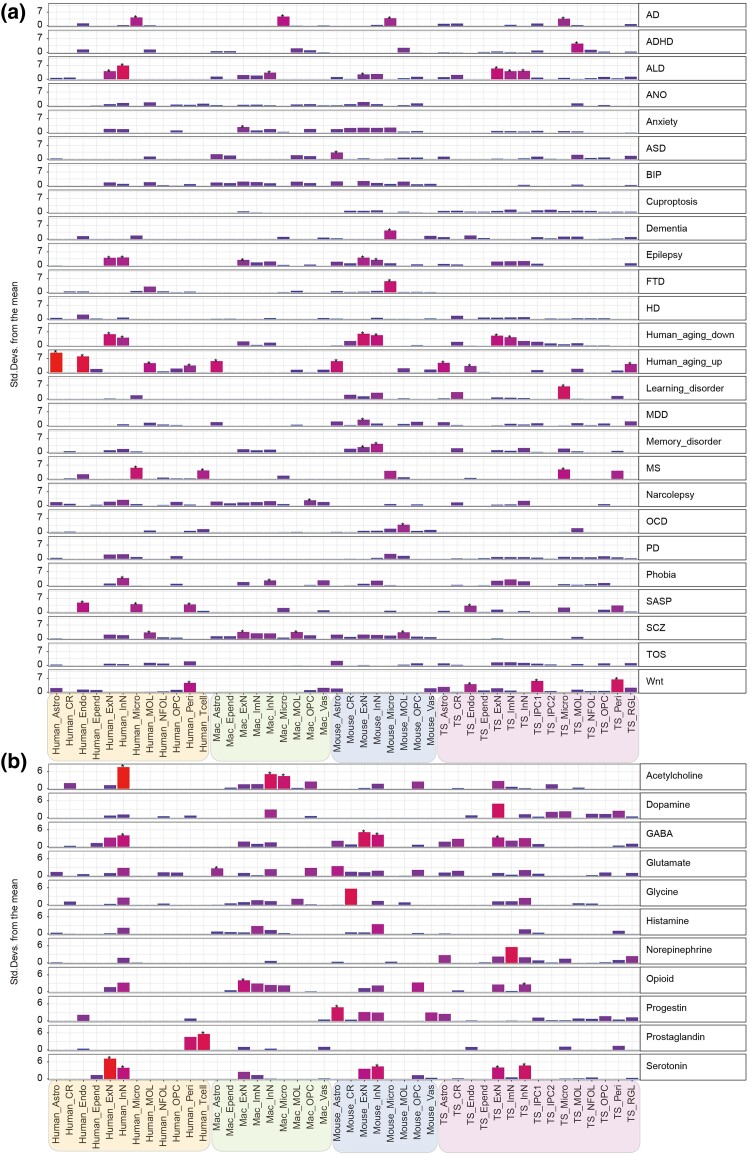
Enrichment of neurological disease genes and neurotransmitter receptors and transporters genes in each cell cluster across species. a) Cell-type enrichment level of 26 gene sets associated with neurological diseases in humans, macaques, mice, and TS. Bar plot showing the standard derivation of a certain disease risk gene set, with species-related cell types indicated below the plot. Asterisks denote the BH-corrected *P*-value < 0.05 calculated using EWCE (permutation test). b) Cell-type enrichment level of 11 gene sets associated with neurotransmitter receptors and transporters in the four species. AD, Alzheimer's disease; ADHD, attention deficit hyperactivity disorder; ALD, alcohol dependence; ANO, anorexia nervosa; ASD, autism spectrum disorder; BIP, bipolar disorder; FTD, frontotemporal dementia; HD, Huntington's disease; MDD, major depression disorder; MS, multiple sclerosis; OCD, obsessive–compulsive disorder; PD, Parkinson's disease; SASP, senescence-associated secretory phenotype; SCZ, schizophrenia; TOS, Tourette syndrome.

To establish connections between neurological diseases and specific cell types and to compare these associations across species, we investigated the enrichment of risk genes associated with nervous system disorders and aging in cell types from four species. These disorders include Alzheimer's disease (AD), attention deficit hyperactivity disorder (ADHD), alcohol dependence (ALD), anorexia nervosa (ANO), anxiety, autism spectrum disorder (ASD), bipolar disorder (BIP), epilepsy, learning and memory disorder, major depression disorder (MDD), narcolepsy, schizophrenia (SCZ), obsessive–compulsive disorder (OCD), multiple sclerosis (MS), Tourette syndrome (TOS), brain aging genes, and aged human Micro gene (HuMi_aged gene set) (Fig. 2 and supplementary table S10, Supplementary Material online). Disease-related genes were collected from previous studies and DisGeNET database (https://www.disgenet.org/). Notably, AD risk genes and HuMi_aged gene set were significantly enriched in microglial populations consistently across four species (Fig. 2a and supplementary fig. S6a, Supplementary Material online). In TS, ADHD risk gene showed significant enrichment in MOL, while ALD risk genes were enriched in ExN, ImN, and InN (Fig. 2a). TS Micro also exhibited significantly higher enrichment of risk genes for learning disorder and MS than that of other species (Fig. 2a). In Wnt signaling pathways, TS Endo, IPC1, and Peri cells demonstrated notable enrichment. Across all species, the upregulated aging-related genes were predominantly enriched in endothelial cells and glia populations, whereas the downregulated aging-related genes were primarily enriched in neurons among humans, mice, and TS (Fig. 2a). To further explore neurotransmitter system involvement, we assessed enrichment scores for 12 neurotransmitter systems across cell types and species (Fig. 2b). Neurotransmitters, which are critical for higher brain functions such as learning, memory, attention, and emotion regulation, were analyzed due to their significant role in neuropsychiatric disorders. As revealed, serotonin and Gama-Aminobutyric Acid (GABA) signaling showed significant enrichment in ExN and InN, aligning with findings in humans (Fig. 2b). These analyses were stratified by developmental stages—infancy, adulthood, and old age. In humans and TS, ExN and glial cell types exhibit significant enrichment for multiple disorder-associated genes during adulthood and senescence (supplementary fig. S6b, Supplementary Material online). Species-specific differences in enrichment patterns highlighted distinct genetic risk landscapes across cell populations. Furthermore, the risk scores of neuropsychiatric disease-associated genes increased significantly with age in the postnatal TS hippocampus, particularly for AD, ADHD, ANO, SCZ, MDD, ASD, BIP, OCD, and TOS, indicating heightened disease susceptibility in the hippocampus with aging (supplementary fig. S6c, Supplementary Material online). Together, these results supported TS present great potentials simulating such as AD, ADHD, ALD, aging, learning disorder, MS, and Wnt signaling-related diseases.

### Age-Related Molecular and Cellular Alteration in the TS Hippocampus

**Fig. 3.**
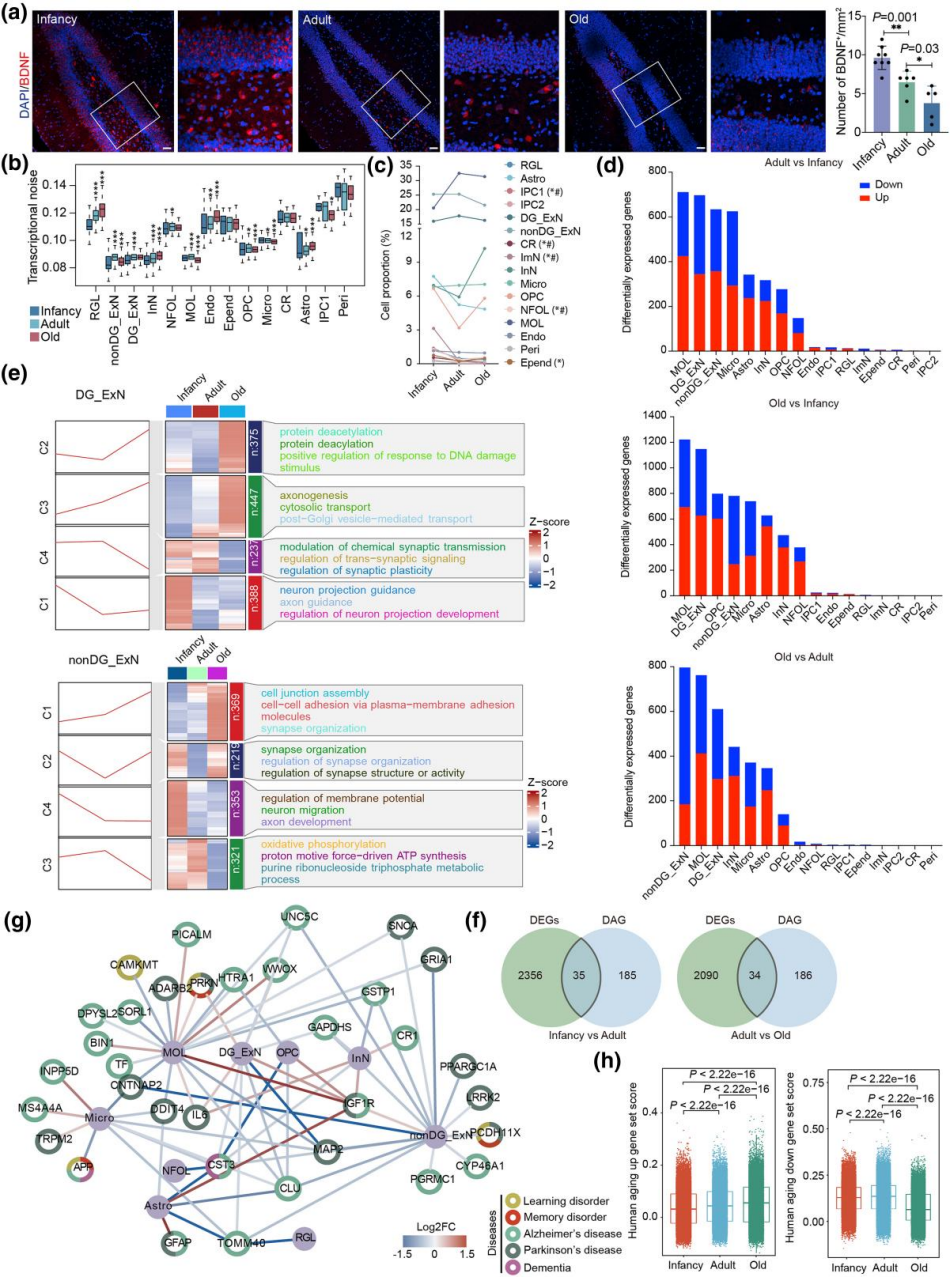
Cellular and molecular aging characteristics of the TS hippocampus. a) Representative microscopic fields and quantification of *BDNF*-positive cells in the hippocampal DG from infancy, adult, and old TS. Blue, DAPI. Scale bar: low magnification, 100 μm; high magnification, 20 μm. *n* = 5 to 8, one-way ANOVA, *P < 0.05, **P < 0.01. The white rectangular box represents the *BDNF*-positive cells. Similar outcomes were obtained in three repeated independent experiments. b) Boxplot showing transcriptional noise in different cell types in infancy, adult, and old TS hippocampus (two-sided Wilcoxon rank-sum test, *P < 0.05, **P < 0.01, ***P < 0.001). c) The cell proportion for each cell type in infancy, adult, and old TS hippocampus (one-way ANOVA, *P < 0.05 for comparison between adult and infancy, ^#^*P* < 0.05 for comparison between old and infancy). d) The bar chart shows the number of differentially expressed genes (DEGs) in different comparison groups (infancy vs adult, old vs adult, and old vs infancy) for each cell type (MAST, FDR-adjusted *P*-value < 0.05, |log_2_FC| > 0.25). e) Variation trend and functional enrichment of DEGs with age in DG_ExN and nonDG_ExN (FDR-adjusted *P*-value < 0.05, |log_2_FC| > 0.25). f) Venn intersected DEGs (FDR-adjusted *P*-value < 0.05, |log_2_FC| > 0.25) with DAGs (infancy vs adult and adult vs old) in the TS hippocampus. DAG, disease-associated gene. g) Network plot showing relationship between DEGs (FDR-adjusted *P*-value < 0.05, |log_2_FC| > 0.25) and aging-related diseases in different cell types in the TS hippocampus. h) Density plot showing gene set scores of human aging-related upregulated (left) and downregulated (right) genes in glial cells and endothelial cells in the TS hippocampus with age (two-sided Wilcoxon rank-sum test).

Next, we performed comprehensive transcriptomic analyses to investigate the molecular features and cellular dynamics of TS hippocampus across postnatal stages. Significant aging-related changes were revealed, particularly in genes associated with neurogenesis and synaptic function. *BDNF*^+^ cell number was notably reduced in the DG region of the TS hippocampus with advancing age (Fig. 3a). The abundance of *BDNF* in the hippocampus is closely correlated with neurogenesis and cognitive behavior (Horowitz et al. 2020; Xiao et al. 2020). It has been shown that senescence is the result of increased transcriptional instability rather than a coordinated transcriptional program and that age-related increased transcriptional noise may lead to altered fate changes and ambiguous cell-type identity (Angelidis et al. 2019). In order to further understand transcriptional stability during aging, we calculated transcriptional noise for different cell types. Transcriptional noise, defined as the variability in gene expression, was significantly increased in neurogenic cells (e.g. NSCs, OPCs), oligodendrocytes, Micro, and endothelial cells with aging (Fig. 3b), consistent with other reports of increased transcriptional instability in aged tissues (Angelidis et al. 2019; Zhang et al. 2021). Besides, we observed the proportions of Micro, InN, and proliferative IPC2 tended to increase with age, while IPC1, CR, and ImN drop dramatically after infancy (Fig. 3c and supplementary table S1, Supplementary Material online), highlighting the dynamic cellular changes in the hippocampus during aging. Using Cacao and Milo analyses (Batiuk et al. 2022; Dann et al. 2022), we highlighted significant changes in cell-type abundance across developmental stages (supplementary fig. S7, Supplementary Material online). These methods, with high sensitivity to subtle abundance differences, confirm the reliability of our cell proportion data and reveal dynamic shifts in key cell populations during aging.

We performed differential gene expression analysis for each cell population to compare three stages of hippocampal development using Model-based Analysis of Single-cell Transcriptomics (MAST). After correcting for potential cofounders, such as sex, and ribosomal and mitochondrial transcription fractions, we found significant changes in gene expression between infancy and adulthood (1,698 differentially expressed genes (DEGs), adjusted *P*-value < 0.05) (supplementary table S11, Supplementary Material online) and between adulthood and senescence (1,714 DEGs, adjusted *P*-value < 0.05) (Fig. 3d and supplementary table S12, Supplementary Material online). The increasing number of DEGs was observed in non-DG_ExN, DG_ExN, Astro, and MOL in aging TS compared with adult and infancy TS (Fig. 3d and supplementary fig. S8, Supplementary Material online). To survey the transcriptional alterations of the TS hippocampus across ages, we performed clustering analysis using the Mfuzz method for DEGs in these cell types across infancy, adult, and old groups. As shown, the gene sets were divided into four clusters (C1 to C4), including upregulated and downregulated genes, according to the trends in their expression as age progressed (Fig. 3e). Among these clusters enriched in DG_ExN, C1 and C4 associated with neuronal development, synaptic plasticity, and synaptic transmission were downregulated with age in TS hippocampus (Fig. 3e). Similar in nonDG_ExN, C3 and C4 associated with neuronal migration, oxidative phosphorylation, and metabolic process were downregulated in aging TS hippocampus (Fig. 3e). Meanwhile, in Astro and MOL, clusters associated with epithelial cell proliferation, cell chemotaxis, and leukocyte activation, respectively, were enriched in oligodendrocytes and endothelial cells and were upregulated with age in TS hippocampus (supplementary fig. S9, Supplementary Material online).

### Age-Related High-Risk Genes and Cellular Communication

Through further comparative analysis, cell-type-specific DEGs were presented for each cell type, since their alteration might compromise the corresponding cellular functions (supplementary fig. S10a–c, Supplementary Material online). We identified five significantly upregulated genes (*RALGDSli7, IGF1R, GATAD2B, MARK3, NPDC1-AS-1*) and 17 significantly downregulated genes (*CST3, LINC-MARPL42-8, LINC-FRMD8-1, TSORFli2504, LINC-HSPA8-21, TTR, MT3, LINC-RPL31-44, NDUFS7, NCAPD2, PCSK1N, CALM1li2, FTH1li4, ACTB, HSP90B1li3, CLU, HSPA5*) across glial types (Astro, OPC, MOL, Micro) and neuronal types (InN, ExN) in the aging TS hippocampus compared with adulthood (Fig. 3d and supplementary fig. S10a and supplementary table S11, Supplementary Material online). *CST3*, a gene involved in presynaptic differentiation (Um et al. 2014), was upregulated in nonDG-ExN, NFOL, and Astro of adult TS hippocampus, but downregulated in neurons, OPC, MOL, and Astro of aging TS hippocampus (supplementary fig. S10a and b, Supplementary Material online), suggesting dysregulated synapse formation during aging. Metallothionein3 (MT3) with neuroprotection in the aging brain is upregulated in glial cells and neurons at adulthood but downregulated in these cells during aging process (supplementary fig. S10a and b and supplementary table S12, Supplementary Material online). *CTNNA3*, associated with the risk of late-onset AD and plasma Aβ levels (Miyashita et al. 2007; Lincoln et al. 2013), is an upregulated gene in glial cells, ExN, and InN in aging TS (supplementary fig. S10c, Supplementary Material online). *IGF1R* exhibited significant upregulation in glial cells and neurons in the senescent TS (supplementary fig. S10c, Supplementary Material online).

To gain insights into the connection between aging and neurodegenerative diseases, we performed a comparative analysis of DEGs in TS hippocampus with gene sets associated with human neurodegenerative diseases, including AD and Parkinson's disease (PD) (Fig. 3f and g and supplementary fig. S10d, Supplementary Material online). We identified high-risk DEGs associated with neurodegenerative disorders, including *PICALM*, *SORL1*, *CST3*, *IGF1R*, *DDIT4*, and *HTRA1*, which were enriched in glial cells and neurons (Fig. 3g), indicating that these cell types are more susceptible to age-related diseases. Importantly, *IGF1R* was significantly upregulated in almost all cell types during TS hippocampal senescence, while *DDIT4*, a regulator of cell growth, proliferation, and survival, was significantly reduced in Micro, Astro, and MOL (Fig. 3g and supplementary fig. S10e, Supplementary Material online), suggesting potential nutrient sensing pathway composed of *IGF1R* and *DDIT4* involved in TS hippocampal senescence. These high-risk genes dysregulated in more than one cell type, such as *IGF1R*, *DDIT4*, and *CST3*, may be potential targets for delaying the onset of cognitive decline and neurodegenerative diseases in the elderly. Moreover, aging-related gene sets scoring delineated that glial cells and endothelial cells showed higher scores for the aging-upregulated genes and that they significantly increased with age, while neurons showed higher scores for aging-downregulated genes, which significantly decreased with age (Fig. 3h). These results indicate a homeostatic imbalance between neurogenic and glial cells, which may lead to impaired regulation of brain function.

To further explore aging-associated changes in cellular interactions, we performed a cell–cell communication analysis, focusing on three major types of communication: Secreted Signaling, extracellular matrix-Receptor, and Cell–Cell Contact. While cellular interactions increased in adulthood, they decreased in old TS (supplementary fig. S11a, Supplementary Material online), a pattern also observed in human and mice hippocampus. Notably, the intensity of cellular communication between non-DG_ExN and DG_ExN, non-DG_ExN and MOL, DG_ExN, and MOL was relatively reduced in old stage compared with adulthood, and the InN- and ependymal-specific cellular communication was relatively increased in old age (supplementary fig. S11b and c, Supplementary Material online). These results highlight age-related imbalances in cellular communication, particularly between neurogenic and glial cells, which could impair neurogenesis and neural signaling. We found that specific communication pathways were prominent at different stages of aging. In infant TS, signaling pathways associated with tissue regeneration, cell migration, and stem cell maintenance (Malanchi et al. 2011; Fernandes et al. 2017; van der Klaauw et al. 2019; Meng et al. 2022) (e.g. *PERIOSTIN, SEMA3, EGF, PDGF, WNT, CLDN*) were significantly elevated, suggesting robust cellular communication that supports neurogenesis, whereas these signaling were significantly reduced in the old group (supplementary fig. S11d, Supplementary Material online). Furthermore, cellular communication associated with inflammatory responses, such as *ANGPTL* (Zhao et al. 2022), *IL6* (Rummel 2016), *CCL* (Ivanovska et al. 2020), *PROS* (Colgan and Taylor 2010), and *ACTIVIN* (Sideras et al. 2013), was significantly enhanced in the aging TS hippocampus (supplementary fig. S11d, Supplementary Material online).

Altogether, our findings revealed age-related molecular and cellular features of the TS hippocampus, demonstrating impaired neurogenesis and neural circuits, and increased inflammatory response as the most affected and potential hallmarks of hippocampal aging.

### Molecular Alterations in the Process of Age-Related Neurogenesis Decline in TS Hippocampus

NSCs have been found existing in adult hippocampal tissues (Hao et al. 2022). In this study, we found the presence of *SLC1A3*^+^*GFAP*^+^*SOX2*^+^*ETNPPL*^+^ RGLs in adult TS hippocampus, suggesting their potential contribution to both neurogenesis and gliogenesis. Comparing the normalized numbers of nuclei across age groups (supplementary table S1, Supplementary Material online), we observed a selective reduction in RGLs, IPC1, and IPC2 in the adult and old groups compared with the infancy groups (Fig. 3c). Interestingly, the numbers of RGLs remained consistent between the adult and old samples (Fig. 3c and supplementary table S1, Supplementary Material online), aligning with reports of sustained but the low-level neurogenesis in adult humans (Sorrells et al. 2018; Tobin et al. 2019), macaques (Zhang et al. 2021; Hao et al. 2022), pigs (Wang et al. 2022a), and mice (Hochgerner et al. 2018). However, IPC1 and IPC2 exhibited a further decline in the old group compared with adults, with a corresponding reduction in non-DG_ExN cells (Fig. 3b and supplementary table S1, Supplementary Material online). Immunofluorescence staining confirmed a significant age-related decrease in *GFAP*/*SOX2* double-positive cells in the subgranular zone (SGZ) of the TS hippocampal DG (supplementary fig. S12a and b, Supplementary Material online), suggesting diminished hippocampal neurogenesis in aged TS.

**Fig. 4.**
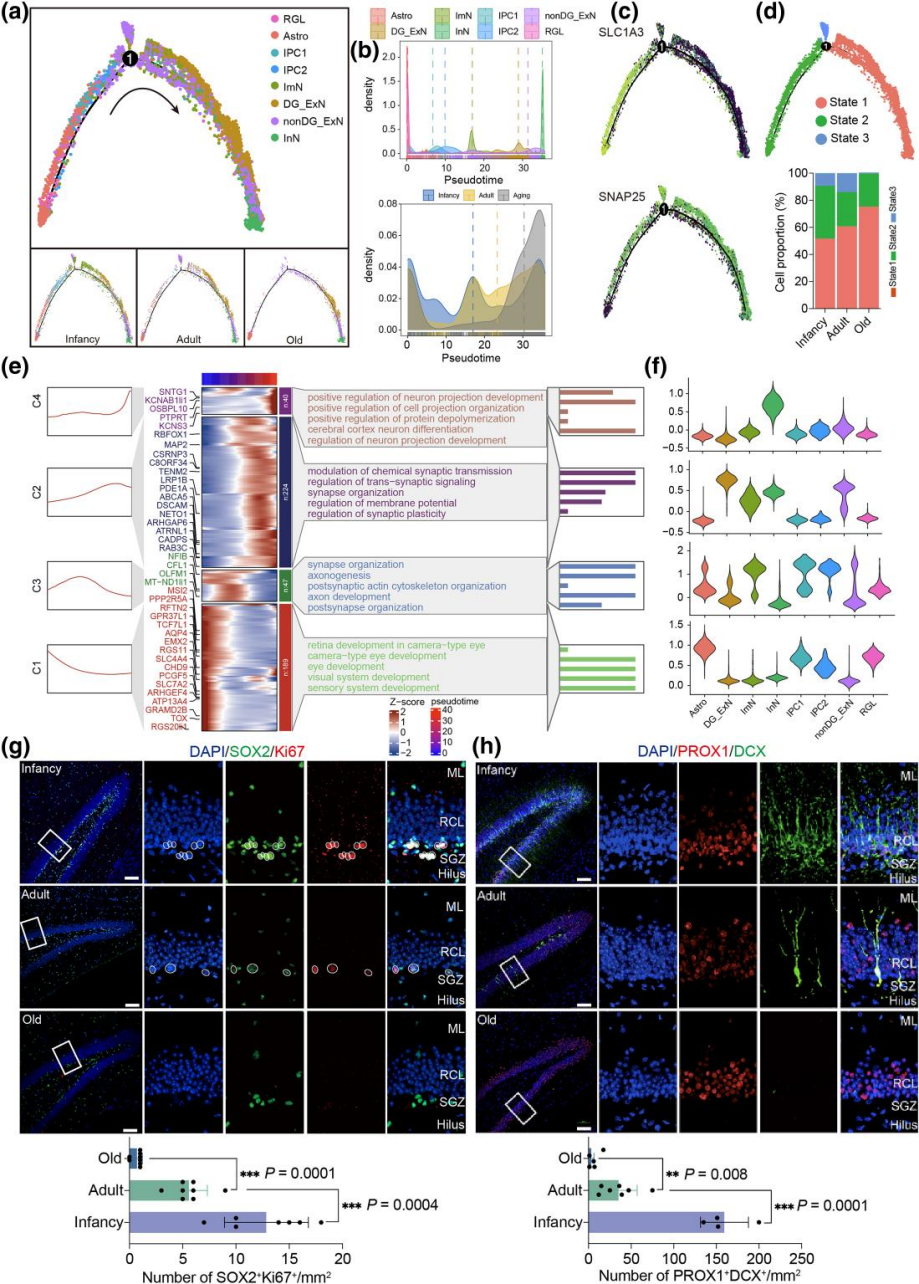
Aging-related cellular and molecular alterations along the trajectories of the neurogenesis. a) Pseudotime analysis of the neurogenic lineage cells in the TS hippocampus. The points are colored by cell types (top) and age (bottom). The arrows indicate the directions of differentiation trajectories. b) Density plot showing the distribution of neurogenic lineage cells and group along the trajectory. c) Pseudotime analysis showing the expression levels of indicated genes along the trajectory from RGLs to neurons of the TS hippocampus. d) Top, pseudotime analysis of neurogenic lineage cells at 3 states in the TS hippocampus. Cells are colored by the states. Bottom, bar plot showing the proportions of neurogenic lineage state in the hippocampus from infancy, adult, and old groups. e) The line and heatmap showing the expression profiles along the pseudotime of DEGs (*q* value < 1 × 10^−4^), which were divided into four clusters (C1 to C4) with the expression pattern and enriched GO terms of the corresponding cluster represented on the right. f) The violin diagram shows the expression levels of the C1 to C4 genes in different cell types. g) Representative microscopic fields and quantification of *SOX2*/*Ki67* double-positive cells in the hippocampal DG from infancy, adult, and old TS. Blue, DAPI. Scale bar: low magnification, 100 μm; high magnification, 10 μm. *n* = 6 to 8, one-way ANOVA, ***P < 0.001. White rectangular boxes represent the focused areas, and white dashed circles indicate the cell bodies of the cells of interest. Similar outcomes were obtained in three repeated independent experiments. h) Representative microscopic fields and quantification of *PROX1*/*DCX* double-positive cells in the DG of the hippocampus from infancy, adult, and aged TS. Blue, DAPI. Scale bar: low magnification, 100 μm; high magnification, 10 μm. *n* = 4 to 7, one-way ANOVA, **P < 0.01, ***P < 0.001. White rectangular boxes represent the focused areas. Similar outcomes were obtained in three repeated independent experiments. ML, molecular layer; GCL, granule cell layer; SGZ, subgranular zone.

To determine the molecular disruptions underlying this age-related impaired neurogenesis, we mapped the differentiation trajectories of RGLs in hippocampal neurogenesis. Using pseudotime analysis, we reconstructed transitions from quiescent RGLs to intermediate progenitor cells (IPC1, IPC2), ImN, and finally to mature neurons (Fig. 4a–c), with no obvious difference in cell-type distribution along the trajectories between the adult and old groups. Molecular cascades were characterized based on pseudotime, revealing differential expression patterns of key marker genes that marked transitions from quiescence to activation, proliferation, and differentiation (Fig. 4c). Clustering stage-specific gene expression along pseudotime identified four distinct expression profiles (C1 to C4), which were analyzed for enriched Gene Ontology terms (Fig. 4e). Genes in C2, which were progressively upregulated with the trajectories and enriched in mature neurons, were associated with synapse transmission and plasticity; in contrast, genes in C3 progressively downregulated along the trajectories and enriched in immature cell types were involved in axonogenesis and development (Fig. 4e and f). Collectively, these results highlight aberrant neurogenesis in the aged hippocampus, with compromised RGLs proliferation at the early stages and impaired neuronal functions at later stages.

Further analysis revealed age-related alteration in histone modification. Notably, *CREBBP* and *HDAC9* were upregulated in RGLs and Astro (supplementary fig. S12c and supplementary table S12, Supplementary Material online). Immunofluorescence staining confirmed the upregulation of *CREBBP* and *HDAC9* in the SGZ of the TS hippocampal DG (supplementary fig. S12d, Supplementary Material online), indicating that altered histone modifications might impact gene expression, contributing to age-associated aberrant neurogenesis. To explore the transcriptional regulatory networks of age-associated DEGs in neuronal lineage cells, we identified key TFs, including *TCF7L2, TCF7L1, ATF2,* and *SOX2* in RGLs and ZMAT4 in ImN (supplementary figs. S1f and S12e, Supplementary Material online). Specifically, GO enrichment analysis of *TCF7L2* target genes revealed regulation of neurogenesis and neurotransmitter uptake (supplementary fig. S12f, Supplementary Material online). Specifically, upregulated genes targeted by *TCF7L2*, such as *NFE2L2*, *ZHX2*, *VGLL4*, *PLP1*, *ZFP36L2*, and *NR2E1*, were enriched for negative regulation of neurogenesis (supplementary fig. S12g, Supplementary Material online). Additional immunostaining of *SOX2* and the proliferating marker *Ki67* validated the presence of proliferating NSCs in the SGZ. However, the number of proliferative NSCs declined significantly with age in TS hippocampus (Fig. 4g). Similarly, the population of immature neurons (*PROX1*/*DCX* double-positive cells) also decreased with age (Fig. 4h). Despite this decline, proliferative NSCs were still present even in aged TS hippocampus. Overall, these findings illustrate age-associated declines in hippocampal neurogenesis, driven by reduced NSCs proliferation and impaired neuronal differentiation and function.

### Divergent Characteristics of RGLs Across Humans, Macaques, TS, and Rodents

**Fig. 5.**
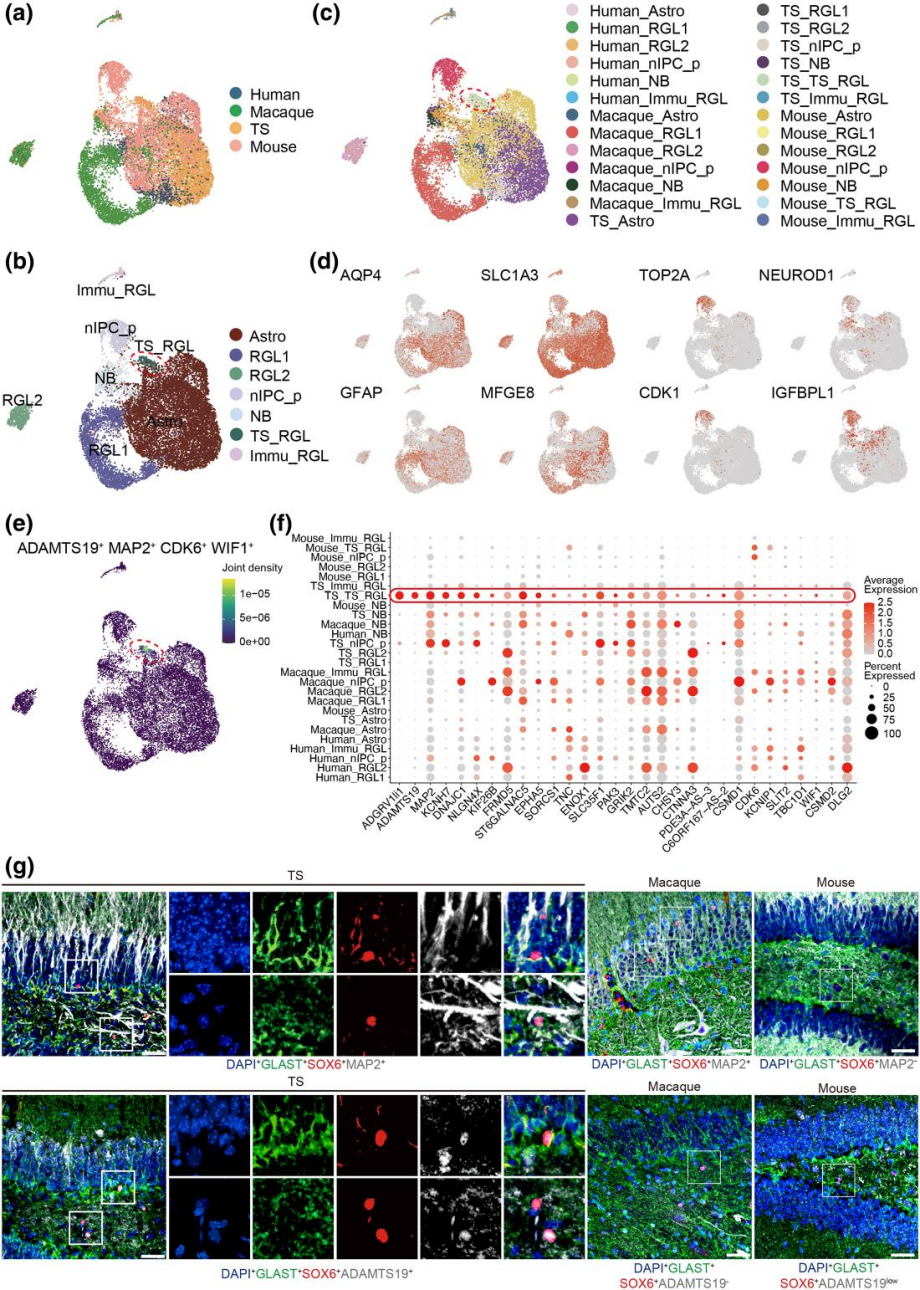
TS-specific NSCs were identified in the TS, macaques, and mice hippocampus. a) UMAP shows the integration of cells from humans, macaques, TS, and mice. b) UMAP shows the distribution of cell types in the integrated dataset (red dashed circles indicate the cell cluster of interest). c) UMAP shows the distribution of species-related cell types in the integrated datasets (red dashed circles indicate the cell cluster of interest). d) The expression of conserved genes in the integrated dataset among the seven distinct NSC subclusters. Cells are colored according to the gene expression levels (red, high; gray, low). e) UMAP shows the distribution of TS-specific NSCs (red dashed circles) in the integrated dataset. f) Bubble dot plots of the top TS-RGL-specific marker genes. The size of the dot indicates expression percentage, and the darkness of the color indicates average expression. g) Triple immunostaining of the TS_RGL markers (*GLAST*/*SOX6*/*MAP2*, and *GLAST*/*SOX6*/*ADAMTS19*) in hippocampal DG of TS (left), macaques (middle), and mice (right). Blue, DAPI. Scale bar: low magnification, 50 μm; high magnification, 10 μm. *n* = 3. White rectangular boxes represent the focused areas. Similar outcomes were obtained in three repeated independent experiments.

Next, we addressed whether RGLs in TS displayed unique features compared with those of well-studied primates and rodents. We integrated our dataset with those from human, macaque, and mouse hippocampal tissues. Through data integration and canonical marker gene identification, we divided the populations into seven cell populations: Astro (*GFAP^+^*, *HES5^+^, AQP4^+^*); RGL1, RGL2, nIPC_p, NB, TS_RGLs, and Immu_RGLs (Fig. 5a–d and supplementary fig. S13a, Supplementary Material online). RGLs were distinguished by their close similarity to Astro (exhibiting expression of *SLC1A3*, *GFAP*, *HES5*, and *SOX2*), along with distinct expression of recently described NSCs markers *ETNPPL*, *MFGE8*, and *LPAR1* (supplementary fig. S13a, Supplementary Material online) (Hochgerner et al. 2018; Wang et al. 2022b). *ETNPPL* is validated as a primate-specific NSCs marker (Wang et al. 2022b). Notably, RGLs also expressed both *NOTCH2* and *PADI2*, which are commonly increased in juvenile and adult mice (supplementary fig. S13a, Supplementary Material online) (Hochgerner et al. 2018). The absence of expression of cell-cycle genes such as *CDK1* and *TOP2A* further confirmed that these cells were in quiescent state, unlike the actively dividing nIPC_p cells (supplementary fig. S13a, Supplementary Material online). NB shared expression of the Eomes marker with nIPC_p cells, *CALB1*, *CABL2*, *SOX4*, *SOX11*, *IGFBP11*, and *DCX*. A cell-type alignment revealed that human, macaque, and TS cells showed higher similarity in gene expression patterns, while mice showed significantly lower alignment scores with other species (supplementary fig. S13b, Supplementary Material online). GO analysis of these cell-type-specific marker genes demonstrated that regulation of neuron projection development, dendrite development, and axonogenesis was enriched for RGLs, IPC, and NB, while astrocyte differentiation for Astro, nuclear division for nIPC_p, regulation of synaptic membrane plasticity for NB (supplementary fig. S13c, Supplementary Material online). Differential analysis showed that early NSCs (RGL1 and RGL2) exhibited similar characteristics across species, whereas later-stage NSCs (nIPC) and NB displayed considerable interspecies variability, suggesting that differences in brain structure and function between species may arise from differences in later NSCs differentiation (supplementary fig. S13c, Supplementary Material online). Further differential analysis identified 27 genes were significantly upregulated in Astro of humans, macaques, and TS, functionally associated with regulation of high-voltage-gated calcium channel activity and synaptic transmission. Meanwhile, 11 genes that were significantly highly expressed in NB of humans, macaques, and TS were linked to synaptic vesicle cycle and synapse organization (supplementary fig. S13d and e, Supplementary Material online). Interestingly, we found that TS_RGLs expressed higher levels of *SOX5* and *SOX6* compared with other NSCs (supplementary fig. S13a, Supplementary Material online). These genes are crucial for the transition from a quiescent state to an activated mitotic state in the adult RGLs under physiological conditions (Li et al. 2022). DEG analysis revealed that TS_RGLs specifically highly expressed *ADAMTS19*, *MAP2*, *CDK6*, and *WIF1* (Fig. 5e and f). Immunofluorescence staining confirmed the presence of specific TS_RGLs in the SGZ of TS hippocampal DG, marked by *GLAST*^+^*SOX6*^+^*ADAMTS19*^+^ and *GLAST*^+^*SOX6*^+^*MAP2*^+^ co-expression (Fig. 5g). Although *GLAST*/*SOX6*/*MAP2* triple-positive cells were observed in the macaque hippocampus, their numbers were low and do not express *ADAMTS19* (Fig. 5g). In contrast, the *GLAST*/*SOX6* double-positive cells were detected in the mice hippocampus, but they do not express *MAP2* and exhibited only low expression of *ADAMTS19* with fewer cell numbers (Fig. 5g). In summary, we identified a distinct NSCs population in the TS hippocampus, characterized by a set of TS- and primate-specific markers, and confirmed the presence of TS-specific NSCs (TS_RGLs) in the SGZ of the TS hippocampal DG at both the RNA and protein levels.

### Characteristics of InN and RC Population in TS Hippocampus

**Fig. 6.**
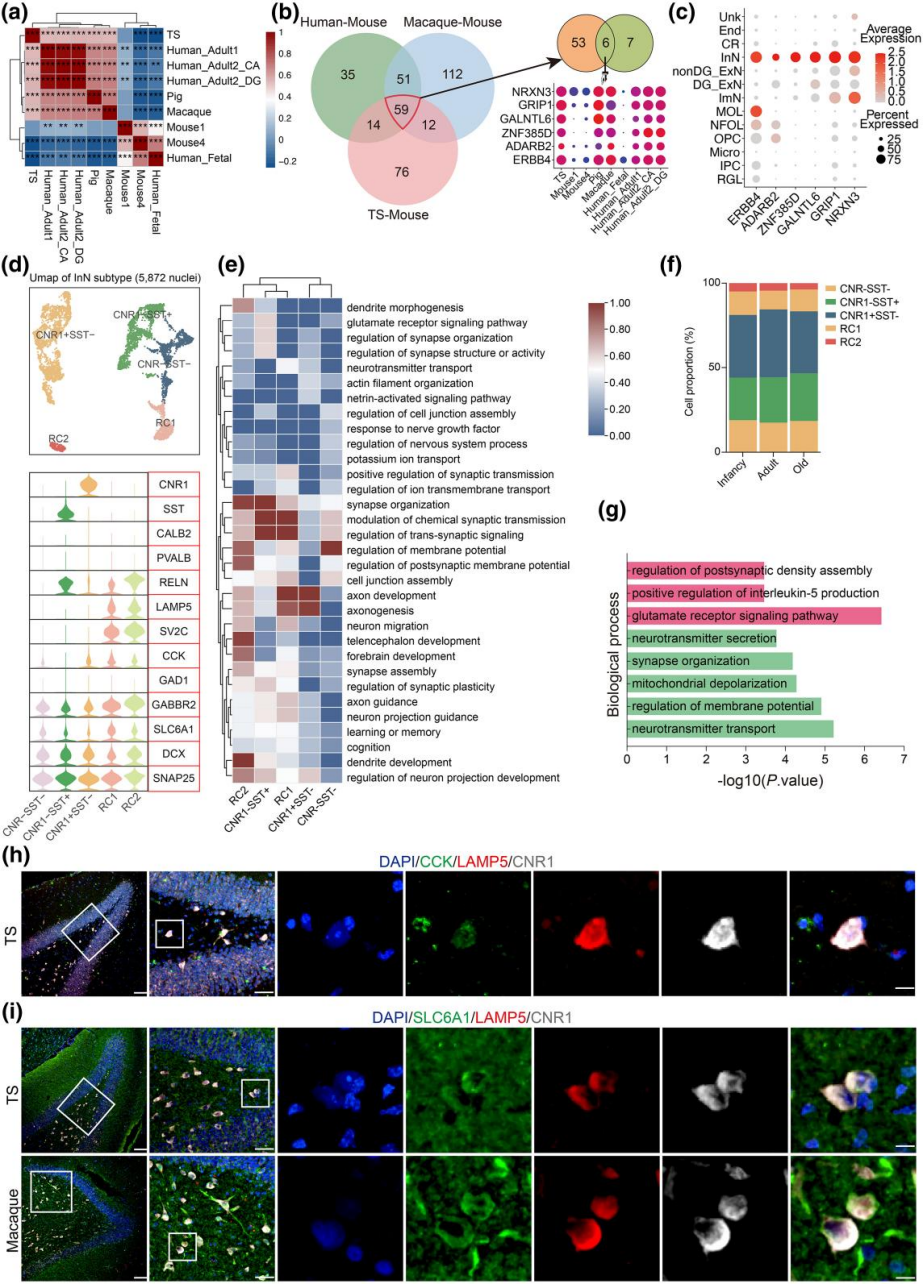
InN subpopulation analysis and identification of the RC population. a) Heatmap shows the correlation of InN across species (Spearman, *P < 0.05, **P < 0.01, ***P < 0.001, significance test for Spearman's rank correlation coefficient using *t*-distribution approximation). b) Left, Venn showing the DEGs between mouse and other species (two-sided Wilcoxon rank-sum test, adjusted *P*-value < 0.05, logFC > 0.25) about InN in hippocampus. Right-top, Venn showing the intersection of shared genes of TS/primate and TS microglia genes (two-sided Wilcoxon rank-sum test, adjusted *P*-value < 0.05, logFC > 0.25). Right-below, dot plot showing the expression of common genes of TS/primate-special and TS microglia-special (adjusted *P*-value < 0.05, logFC > 0.25). c) Dot plot showing the average expression of TS-specific genes for microglia in each cell type of TS hippocampus. d) Top, visualization of InN subclusters using UMAP. Below, the violin plot demonstrates the expression of the canonical marker genes of InN subclusters. RC, rosehip-like InN. e) Heatmap showing the enriched GO terms of cell-type-specific marker genes of different InN subclusters in the TS hippocampus, with their enriched functional annotations on the right. f) Bar plot showing the proportions of InN subclusters in the hippocampus from infancy, adult, and old groups. g) Enriched GO terms for RC2 aging-related genes from adult and old groups. h) Representative microscopic fields of *CCK*/*LAMP5*/*CNR1* triple-positive cells in the hippocampal DG from adult TS. i) Representative microscopic fields of *SLC6A1*/*LAMP5*/*CNR1* triple-positive cells in the DG of the hippocampus from adult TS (above) and macaques (below). Blue, DAPI. Scale bar: low magnification, 100 μm; high magnification, 50 μm, 10 μm. *n* = 3. White rectangular boxes represent the focused areas.

Impaired GABA-mediated neurotransmission has been implicated in many neurologic diseases, including epilepsy, intellectual disability, and psychiatric disorders (Hunt et al. 2013). To characterize the conservation and divergence of InN (GABAergic/InN) across species, we analyzed single-cell InN datasets from mice, pigs, TS, macaques, and humans. Spearman’s correlation analysis revealed a higher correlation between TS and pigs, macaques, and humans compared with mice (Fig. 6a). To obtain the unique marker gene for InN in TS and macaques, we performed a DEG analysis comparing mice with TS, macaques, and humans (Fig. 6b). The results showed that *NRXN3, GRIP1, GALNTL6, ZNF385D, ADARB2*, and *ERBB4* were consistently highly expressed in the inhibitory nerves of TS, pigs, macaques, and humans as compared to mice (Fig. 6b and c).

In TS, InN were subclassified as *CNR1*^+^*SST*^−^, *CNR*^−^*SST*^+^, *CNR1*^−^*SST*^−^, and *CCK*^+^*CNR1*^−^*SST*^−^*CALB2*^−^*PVALB*^−^ rosehip cells (RCs) (Boldog et al. 2018) (Fig. 6d). A unique population of RCs, a specialized interneuron subtype previously described in human cortical layer 1 but absent in rodents, was identified in the TS hippocampus (Fig. 6d). These cells exhibited a distinct gene expression profile, with GO enrichment showing associations of *CNR*^−^*SST*^+^ InN with regulation of membrane potential and synaptic signaling, *CNR*^−^*SST*^+^ InN with axonogenesis and axon development (Fig. 6e). RC1-related genes were linked to neurogenesis and synapse signaling, while RC2-related genes were enriched in neurogenesis, hippocampus development, regulation of synaptic plasticity, and learning or memory (Fig. 6e). Comparative analysis unveiled a higher proportion of *CNR1*^+^*SST*^−^ cells in each age groups than other subtypes (Fig. 6f), suggesting an essential role for *CNR1*^+^*SST*^−^ InN in hippocampal development and synaptic plasticity in TS. Additionally, the decline of RC2 in aged TS may contribute to the reduced neuronal function and altered cognition (Fig. 6f). GO analysis of genes downregulated in aged RC2 indicated enrichment in the neurotransmitter transport and regulation of membrane potential, while upregulated genes were enriched in the glutamate receptor signaling pathway, positive regulation of interleukin-5 production, and regulation of postsynaptic density assembly (Fig. 6g). However, RCs (*CCK^+^LAMP5^+^CNR1^−^*, *SLC6A1^+^LAMP5^+^CNR1^−^*) were not found in the TS and macaque hippocampus by immunofluorescence staining, possibly due to their low abundance or discrepancies between RNA and protein expression (Fig. 6h and i). In summary, we identified TS-RCs with transcriptional profiles similar to human RCs, suggesting their potential role in TS hippocampal aging.

### TS-specific Microglial Features and Age-Related Neuroinflammation in the TS Hippocampus

**Fig. 7.**
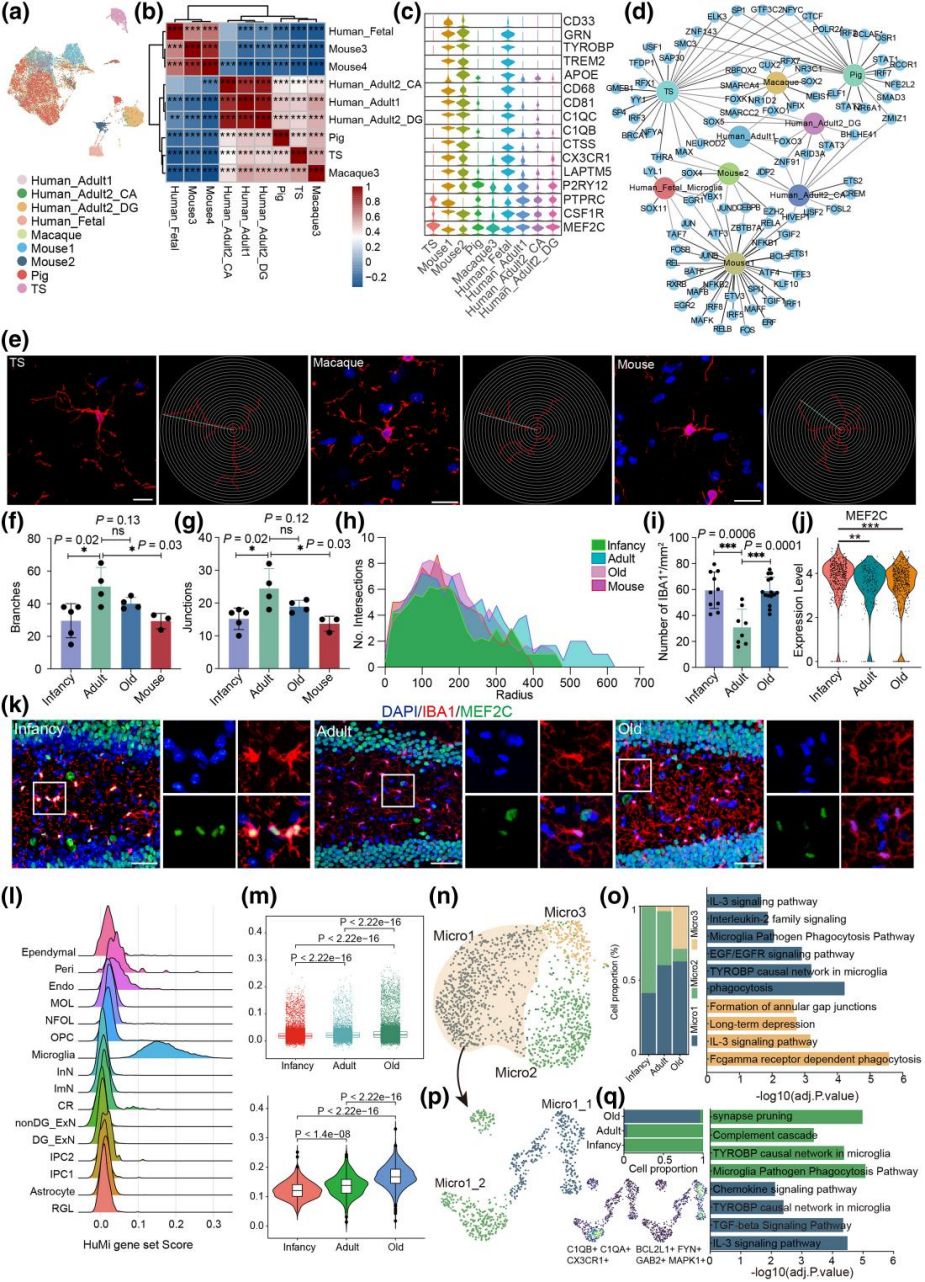
Transcriptional heterogeneity of cells derived from TS microglia. a) UMAP shows the distribution of cell types from humans, macaques, TS, pigs, and mice. b) Heatmap shows the correlation of microglia across species (Spearman, *P < 0.05, **P < 0.01, ***P < 0.001, significance test for Spearman's rank correlation coefficient using *t*-distribution approximation). c) The violin plot demonstrates the expression of the canonical marker genes of microglia across species. d) Network plot show species-specific transcriptional regulators of microglia. e)–h) Manual tracking and Sholl analysis-based quantification of cell morphology of hippocampal *IBA1*-positive microglia (red). Each symbol represents one individual; three to five cells analyzed per individual. Sholl analysis: step size = 20 μm. Blue, DAPI. Scale bar: 25 μm. Similar outcomes were obtained in three repeated independent experiments. One-way ANOVA, *P < 0.05, ns, not significant. i) Quantification of *IBA1*-positive cells in the hippocampal DG from infancy, adult, and old TS. One-way ANOVA, ***P < 0.001. j) Violin plots showing expression levels of *MEF2C* in microglia in the TS hippocampus from infancy, adult, and old groups (two-sided Wilcoxon rank-sum test, **P < 0.01, ***P < 0.001). k) Representative microscopic fields of *IBA1*/*MEF2C* double-positive cells in the hippocampal DG from infancy, adult, and old TS. Blue, DAPI. Scale bar: low magnification, 50 μm; high magnification, 10 μm. *n* = 3. l) Density plot showing gene set scores of HuMi_Aged microglia genes in different cell types in the TS hippocampus. m) Top, boxplot showing increasing gene set scores in TS hippocampus with age. Bottom, violin plots showing increasing HuMi_Aged gene set scores in microglia in the TS hippocampus from infancy, adult, and old groups (two-sided Wilcoxon rank-sum test). n) Visualization of microglia subclusters using UMAP. o) Left, bar plot showing the proportions of microglia subclusters in the hippocampus from infancy, adult, and old groups. Right, enriched GO terms in Micro1 and Micro3. p) Visualization of Micro1 subclusters using UMAP. q) Left-top, bar plot showing the proportions of Micro1 subclusters in the hippocampus from infancy, adult, and old groups. Left-bottom, UMAP showing the distribution of cluster-related DEGs in the Micro1. Right, enriched GO terms in Micro1_1 and Micro1_2.

Micro malfunction is implicated in various neurodegenerative and neuropsychiatric disorders. We identified a distinct microglial population in the TS hippocampus and analyzed single-cell Micro datasets from five different species: mice, pigs, TS, macaques, and humans. Cross-species analysis showed strong alignment across species, with TS Micro exhibiting greater similarity to macaques, followed by pigs and humans, and lower similarity to mice (Fig. 7a and b). Conserved microglial markers, including *CSF1R, PTPRC, P3RY12, CX3CR1,* and *MEF2C*, are expressed across species, (Fig. 7c), with *MEF2C* identified as a key conserved TF in microglial evolution, consistent with previous studies (Wang et al. 2022a). Immunofluorescent staining confirmed *MEF2C* expression in *IBA1*-positive Micro in the hippocampus of TS, mice, and macaques (supplementary fig. S14a, Supplementary Material online). Single-cell genetic regulatory network inferring and clustering analyses revealed TS shared more common TFs with pigs, followed by macaques (Fig. 7d). Furthermore, DEG analysis identified TS-specific microglial markers, including *PLD5, CYTL1, BLNK, PIPOX, MEF2C*, and others (supplementary fig. S14b, Supplementary Material online), as well as primate-shared markers, such as *LRMDA, MAN1A1, CAMK1D,* and *GAB2* (supplementary fig. S14c, Supplementary Material online). *IBA1* immunohistochemistry-based Sholl analysis revealed that the TS, macaques, and mice were positive for parenchymal *IBA1*^+^ cells (Fig. 7e), and TS Micro had longer dendrites, more branches, and larger radiation areas than mice, aligning more closely with macaques (Fig. 7e–h). These findings suggest that TS Micro exhibit a hybrid profile, with transcriptomic and morphological features closer to primates than to rodents.

Micro are key players in brain inflammation (Guo et al. 2024), and we next examined their molecular and functional alterations during aging in the TS hippocampus. The number of *IBA1*-positive Micro increased significantly in the aged (Fig. 7i). Sholl analysis showed corresponding increase in dendrite length, branching, and radiation areas in adult and aged TS hippocampus relative to infancy (Fig. 7f–h and supplementary fig. S14d and e, Supplementary Material online). Interestingly, *MEF2C* expression decreased with age at both RNA and protein levels (Fig. 7j and k and supplementary fig. S14f, Supplementary Material online), consistent with reports showing that *MEF2C* deficiency exacerbates microglial activation and behavioral deficits during immune challenges in mice during aging (Deczkowska et al. 2017). Then, we analyzed the HuMi_Aged gene set, which were significantly enriched in TS Micro and increased with age both at overall hippocampal levels and within Micro (Fig. 7l and m). Micro were further subclustered into three populations: Micro1, Micro2, and Micro3 (Fig. 7n), with HuMi_Aged gene set significantly enriched in Micro1 and Micro3, and Micro1 and Micro3 numbers significantly increasing in aged TS (Fig. 7o and supplementary fig. S15a and b, Supplementary Material online). The transcriptional regulatory network revealed a panel of key age-associated TFs of Micro, such as *ETV6, TCF7L2*, and *TAL1* (supplementary fig. S15c, Supplementary Material online). *TCF7L1/2, FOXO1/3, SOX2,* and *LEF1* (supplementary table S14, Supplementary Material online) were core TFs involved in the *WNT* signaling pathway. The gene set score analysis for the canonical *WNT* signaling pathway demonstrated an increase of the gene set score in aged samples (supplementary fig. S15d, Supplementary Material online). Through age-related DEGs analysis, a panel of genes related to microglial activation, such as *CD74, TLR4, NFKB1, P2RY6, IL2RA, IL18, TGFBR1, ITGAM,* and *IL6ST,* were upregulated in aged Micro compared with their younger counterparts (supplementary fig. S15e, Supplementary Material online). In particular, Micro2 represents a resting state of Micro expressing genes such as *P2RY12* and *SALL1*, while Micro1 and Micro3 populations were a group of active Micro, expressing proinflammatory markers such as *CD74, TLR4, TYROBP*, *IL6ST*, *TGFBR1*, *IL18*, and *NFKB1* at high levels (supplementary fig. S15e, Supplementary Material online). GO enrichment analysis inked Micro1 to phagocytosis and inflammatory response (Fig. 7o). Active Micro exert beneficial effects via phagocytosis and detrimental effects by secreting cytotoxic cytokines. To detail the nature of active Micro, Micro1 was divided into two subclusters, Micro1_1 and Micro1_2 (Fig. 7p). Micro1_2, predominantly found in young TS samples, expressed antiinflammatory genes such as *C1QB, C1QA,* and *CX3CR1* and was associated with synaptic pruning and phagocytosis of debris from apoptotic cells during the early postnatal stages (Fig. 7q and supplementary table S14, Supplementary Material online). In contrast, Micro1_1, enriched in aged TS samples, expressed proinflammatory markers *BCL2L1, FYN, GAB2*, and *MAPK1* and was linked to chemokine and *IL-3* signaling pathways, characteristic of aging-associated microglial activation (Fig. 7q and supplementary table S14, Supplementary Material online).

In summary, TS Micro exhibit age-dependent changes in morphology, function, and transcriptional profiles. During early stages, active Micro contribute to hippocampal development through phagocytic activity, while in aging, they shift to a predominantly proinflammatory role, potentially contributing to neuroinflammation and hippocampal dysfunction in TS.

### Transcriptomic Diversity of Oligodendrocyte Lineages in Postnatal TS Hippocampus

**Fig. 8.**
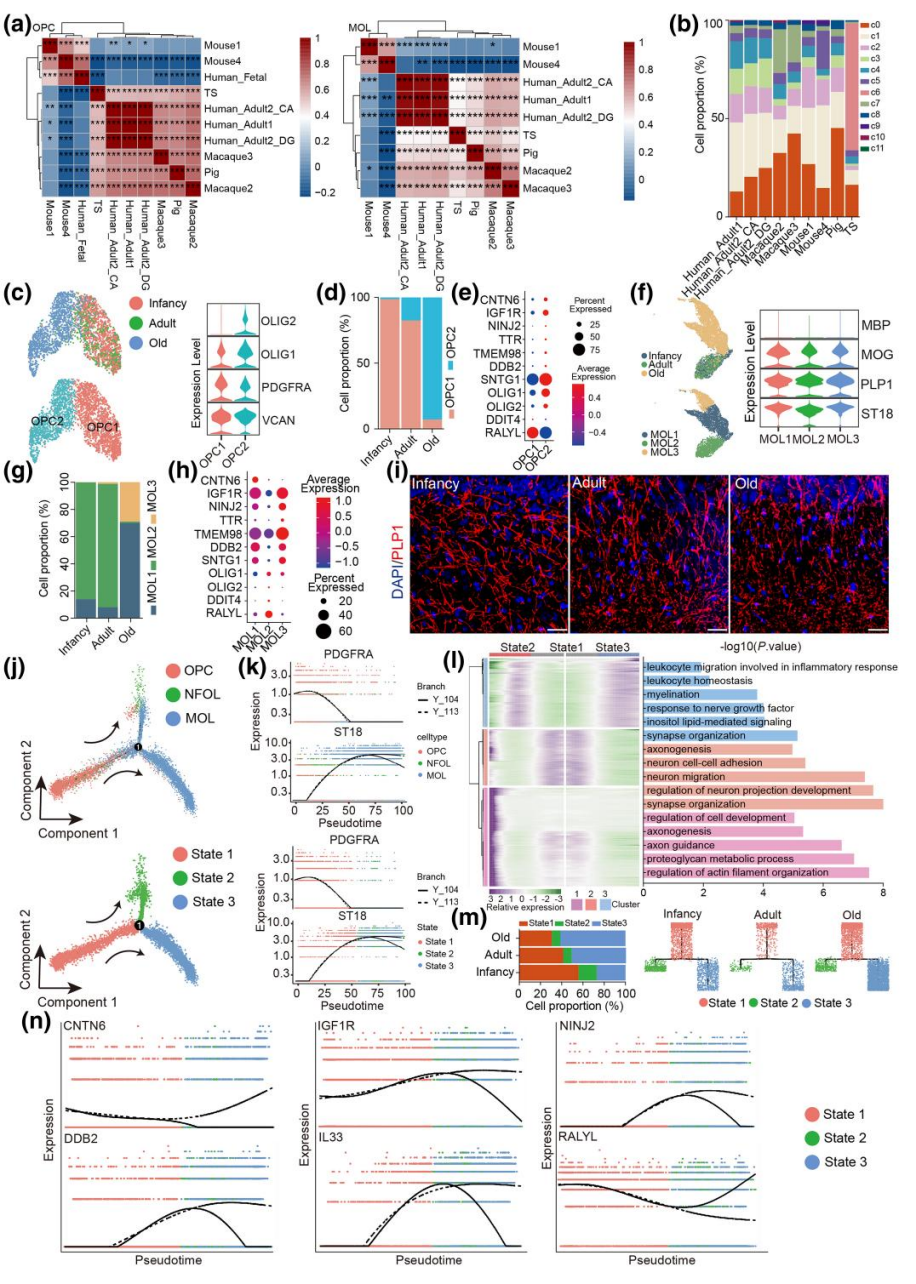
Identification of the OPC and MOL populations in TS hippocampus. a) Left, heatmap showing the correlation of OPC across species. Right, heatmap showing the correlation of MOL across species (Spearman, *P < 0.05, **P < 0.01, ***P < 0.001, significance test for Spearman's rank correlation coefficient using *t*-distribution approximation). b) The cell proportions for each cluster in different species. c) Left, visualization of OPC subclusters using UMAP. Right, violin plot showing the average expression of marker genes for the OPC subclusters. d) Bar plot showing the proportions of OPC subclusters in the hippocampus from infancy, adult, and old groups. e) Dot plot showing cluster-related DEGs. f) Left, visualization of MOL subclusters using UMAP. Right, violin plot showing the average expression of marker genes for the MOL subclusters. g) Bar plot showing the proportions of MOL subclusters in the hippocampus from infancy, adult, and old groups. h) Dot plot showing cluster-related DEGs. i) Representative microscopic fields of *PLP1*-positive cells (red) in the hippocampal CA3 from infancy, adult, and old TS. Blue, DAPI. Scale bar: 100 μm. Similar outcomes were obtained in three repeated independent experiments. j) Pseudotime analysis of OPC and MOL in the TS hippocampus. Cells are colored by the cell types (top) and the states (bottom). The arrows indicate the directions of differentiation trajectories. k) Pseudotime analysis showing the expression levels of indicated genes along the trajectory from OPC to MOL of the TS hippocampus. The points are colored by cell type (top) and cell state (bottom). l) Heatmap showing the expression profiles along the pseudotime of top DEGs (*q* value < 1 × 10^−4^) in MOL and OPC, which were then divided into three clusters (C1 to C3) with the expression pattern and enriched GO term of the corresponding cluster represented on the right. m) Left, bar plot showing the proportions of different states of MOL in the hippocampus from infancy, adult, and old groups. Right, the tree plots showing the state of OPC to MOL from infancy, adult, and old groups. n) Pseudotime analysis showing the expression levels of DEGs along the trajectory from OPC to MOL of the TS hippocampus. The points are colored by state.

OPCs are the most proliferative cells in the CNS, generate MOL throughout life, and potentially contribute to circuit formation and function (Allen and Lyons 2018). Single-cell analysis has revealed heterogeneity among oligodendrocytes in both mice and humans CNS (Jäkel et al. 2019). To explore this diversity, we integrated OPC and MOL single-cell datasets from TS, pigs, mice, macaques, and humans. Similar to InN, the transcriptomic profiles of TS OPC and MOL were closer to those of macaques and pigs (Fig. 8a), and 12 distinct hippocampal cell populations were conserved across species (Fig. 8b). TS OPCs were then subclustered into two populations (OPC1 and OPC2) with distinct gene expression profiles (Fig. 8c). Notably, OPC2 numbers increased with age (Fig. 8d), and DEG analysis revealed elevated expression of myelin regeneration related genes, including *CNTN6, IGF1R, NINJ2, TTR, TMEM98, DDB2*, and *SNTG1* (Fig. 8e). TS MOLs were subclustered into three populations (MOL1, MOL2, and MOL3), with MOL1 and MOL3 numbers increasing with age (Fig. 8f and g). DEG analysis showed a significant upregulation of myelin-related genes such as *CNTN6, IGF1R, NINJ2, TTR, TMEM98, DDB2*, and *SNTG1* in MOL1 and MOL3 (Fig. 8h), suggesting age-associated functional adaptations. However, despite an increase in *PLP1*-positive puncta in aged TS hippocampus, disrupted myelin sheaths were more prevalent (Fig. 8i), indicating myelin-related dysfunction. To trace age-related molecular changes, we reconstructed the developmental trajectory from OPC to MOL using pseudotime analysis (Fig. 8j). Differential expression of representative marker genes highlighted the sequential transition along this trajectory (Fig. 8k). Clustering of stage-specific gene expression along the pseudotime revealed three distinct clusters (C1 to C3) with distinct functional profiles (Fig. 8l): C1 was enriched for genes involved in regulation of actin filament organization and proteoglycan metabolic process, C2 for synapse organization and neuron migration, and C3 for leukocyte migration involved in inflammatory response and leukocyte homeostasis (Fig. 8l). Interestingly, C3 was specifically expressed in MOL subtypes (MOL1 and MOL3), whose populations expanded at an advanced age (Fig. 8m and supplementary fig. S15f–k, Supplementary Material online). Furthermore, joint analysis of cluster-specific and age-related DEGs identified hub genes, including *CNTN6, IGF1R, NINJ2, DDB2, IL33*, and *RALYL*, as key regulators of oligodendrocyte proliferation and aging (Fig. 8n).

In summary, TS oligodendrocyte lineages exhibit distinct transcriptomic diversity, with age-associated shifts in OPC and MOL populations contributing to myelin regeneration pathways but also to increased myelin dysfunction in aged TS hippocampus.

### Identification of Proliferating Endo Cells in the TS Hippocampus During Aging

Brain Endo cells, the major component of the neurovascular unit, are implicated in neuronal degeneration and neurodegenerative disorders (Hansen et al. 2025; Xia et al. 2025). In this study, we observed an increase in the Wnt gene set score in the aged samples (supplementary fig. S16a, Supplementary Material online), highlighting the role of Wnt signaling in endothelial activation and differentiation under brain inflammation (Liebner et al. 2018). Endo cells were subclustered into three populations (Endo1, Endo2, and Endo3) with distinct gene expression profiles. The number of Endo1 increased with age (supplementary fig. S16b and c, Supplementary Material online), and aged Endo1 cells showed elevated Wnt signaling activity (supplementary fig. S16d, Supplementary Material online). GO term analysis revealed that Endo1 was enriched for genes associated with proteasome, leukocyte activation, Wnt signaling pathway, endothelial development, and platelet aggregation (supplementary fig. S16e and f, Supplementary Material online). Notably, genes related to proteasomes (*PSMAs* and *PSMBs*) were elevated in the aged TS hippocampus. The ubiquitin-proteasome system, essential for essential for degrading unwanted or misfolded proteins (Schmidt et al. 2021), was impaired with age, leading to the accumulation of neurotoxic aggregates such as β-amyloid, tau, and α-synuclein, which are associated with neurodegenerative diseases such as AD, PD, Huntington's disease, and amyotrophic lateral sclerosis (supplementary fig. S16g, Supplementary Material online). Considering that age-related differential genes in MOL and Endo populations both regulate leukocyte activation, we performed cell communication analysis. As a result, we observed a significant increase in microglial signals derived from the MOL and Endo in the aged TS hippocampus, with significantly elevated *CD74* and *NOTCH* signaling (supplementary fig. S16h and i, Supplementary Material online). *CD74* and *NOTCH* signaling have been reported to exacerbate Micro-mediated neuroinflammation and contribute to neuropathological conditions (Vandenbark et al. 2019). In summary, these findings indicate identify proinflammatory endothelial cells and their molecular features during hippocampal aging in TS.

These changes suggest the formation of a deleterious microenvironment in the aged TS hippocampus, potentially exacerbating neuroinflammation and facilitating the progression of age-related neurodegenerative diseases.

## Discussion

The COVID-19 pandemic has significantly impacted research, limiting the availability of macaques for experimental studies. Although recent advancements in organoid and cloning techniques have offered promising alternatives for mimicking human biology, in vitro models fail to fully replicate the complexities of in vivo systems. This necessitates the research for new animal models to replace macaques. As a primate-like species, TS show high similarities with humans in physiology, anatomy, genetics, and metabolism (Hochgerner et al. 2018; Hao et al. 2022), making it a promising alternative for studying complex human diseases. In this study, we generated the first age-related single-nucleus transcriptomic atlas of the TS hippocampus. Based on their unique transcriptional signatures, we identified 15 distinct cell types, comprising the neurogenesis lineage, oligodendrocyte lineage, and niche cells. Cross-species comparisons revealed notable similarities and differences among TS, mice, macaques, and humans, further highlighting the potential of TS as an animal model. The present study not only provides a comprehensive single-nucleus transcriptomic landscape of TS hippocampus but also present a detailed comparison with other species for the first time. These insights enhance our standing of hippocampal biology and underscore the value of TS as a versatile and advanced model for scientific research.

This study compared hippocampal cell diversity and molecular features among TS, humans, macaques, and mice, revealing that TS are more transcriptomically similar to macaques and humans than to mice. Consistent with previous reports from mice (Hochgerner et al. 2018), macaques (Zhang et al. 2021; Franjic et al. 2022; Hao et al. 2022; Wang, Wang et al. 2022b) and humans (Franjic et al. 2022), we identified three major hippocampal cell lineages in TS: neurogenic lineage cells (RGLs, ImN, ExN, cInN, and CR), oligodendrocyte lineage cells (OPC, NFOL, and MOL), and niche cells (Micro, Astro, Endo, Peri, and Epend). Analysis of single-cell transcriptomes showed most classical marker genes for cell types were conserved among species, though with differing expression abundance. *SLC1A3 (GLAST)*, encoding a glutamate transporter, was highly expressed in Astro and RGLs of these four species, suggesting a conserved role in glutamatergic synaptic transmission. Similarly, *GFAP* and *AQP4* were highly enriched in Astro and RGLs, though expressed at lower levels than *SLC1A3*. Glutamate, the main excitatory neurotransmitter in the mammalian CNS, is removed from the synaptic cleft by sodium-dependent glutamate transporters (Jackson et al. 2001). Adult Astro play a critical role in glutamatergic synaptic input and maturation of adult-born hippocampal neurons and local dendritic spines (Sultan et al. 2015). These results suggest that *SLC1A3* is central to the cross-species conservation of synaptic transmission regulated by Astro. Although *GAD1* and *GAD2* are recognized as InN marker genes, they are hardly expressed in TS, whereas *ADARB2* (*ADAR3*) and *GRIP1* exhibited higher expression levels in TS InN. *ADAR3,* a catalytically inactive member of the Adenosine Deaminase Acting on RNA (*ADAR*) protein family, is exclusively expressed in the brain. While the reasons for the catalytic incapability of *ADAR3* have not been defined, its biological function in learning and memory may suggest a central role TS InN in hippocampal memory formation (Wang et al. 2019a). Similarly, glutamate receptor interacting protein 1 (*GRIP1*), an *AMPAR*-binding protein regulating the trafficking and synaptic targeting of *AMPARs*, is essential for long-term potentiation, learning, and memory (Tan et al. 2020). These findings suggest that TS InN may play a pivotal role in hippocampal memory processes. *DCX*, a widely adopted marker of NB and immature neurons (Tobin et al. 2019), is highly expressed in InN across species, suggesting that *DCX* is not only a specific marker for NB or immature granule neurons or that InN may share functional role with ImN. We also observed high expression of *MEF2C* across species, supporting its role as conserved TFs in microglial evolution, consistent with previous studies (Wang et al. 2022a). Furthermore, microglial *TYROBP*, a network hub and driver of sporadic late-onset AD (Zhang et al. 2013), was highly expressed in TS Micro. As a cytoplasmic adaptor for *TREM2* and other receptors, *TYROBP* is essential for microglial activation. However, *TREM2* expression was absent in TS hippocampus, suggesting that *TYROBP* signaling in TS Micro may function independently of *TREM2* and potentially play a foundational role in establishing the disease-associated Micro phenotype. Morphological analysis revealed that TS Micro exhibited longer dendrites, more branches, and larger radiation areas than mice, whereas the microglial morphology in mice more closely resembled that of young TS, highlighting species-specific developmental differences. Unexpectedly, we identified primate-specific rosehip cells in the TS hippocampus, which play a role in regulating synaptic plasticity, long-term potentiation, and neurogenesis in the TS. The reduction of rosehip cells in the TS hippocampus and the downregulation of the ability to regulate membrane potential and neurotransmitter transmission suggested that it plays a role in hippocampal senescence in TS.

Neurological diseases such as AD, ADHD, ALD, ANO, anxiety, ASD, BIP, epilepsy, learning and memory disorders, MDD, narcolepsy, SCZ, OCD, TOS, and brain aging have been extensively studied in the human brain, and significant proportions of genes have been proposed to be closely related to these diseases (Lu et al. 2004; Olah et al. 2018; Yang et al. 2022). This study systematically examined the enrichment of disease-associated risk genes across cell types from four species (humans, TS, macaques, and mice) to establish connections between neurological diseases and specific cell populations. AD and the HuMi_aged gene set were consistently enriched in Micro across all species, highlighting the central role of Micro in neuroinflammation and neurodegeneration. TS Micro, in particular, exhibited higher enrichment scores for Learning disorders and MS compared with other species, reinforcing their utility as a model for studying Micro-related neuroinflammatory processes. ADHD risk genes were significantly enriched in TS MOL, suggesting a potential link between myelin-related dysfunction and ADHD. ALD risk genes were enriched in ExN, ImN, and InN in TS, underscoring the role of neurotransmitter imbalances and synaptic dysregulation in addiction. TS hippocampus demonstrates remarkable potential for modeling diseases such as AD, ADHD, ALD, aging, learning disorders, MS, and Wnt signaling-related conditions, offering unique insights into species-specific and conserved mechanisms at the cellular and molecular levels. These results establish TS as a promising model for studying a broad range of neurological and psychiatric disorders.

Differences in intercellular communication contribute to diverse neuronal circuits which may underlie behavioral and emotional differences across species (Heller et al. 2020). We found that various signals are conserved across species, with differences in intensity and number. Particularly, *EGF, PTN, FGF*, *VEGF*, and *IGF* signaling pathways are conserved across species, serving a main function among RGLs, neurons, and oligodendrocytes across humans, macaques, and TS. Adult neurogenesis in the DG arises from quiescent RGLs and is supported by niche factors like *EGF* (Zhou et al. 2018). In the subventricular zone (SVZ), *EGFR* signaling promotes NPC proliferation and migration, and enhanced *EGFR* signaling in vivo expands NPC pool while reducing NSCs number and self-renewal (Aguirre et al. 2010). In the DG, *EGF* signaling helps maintain NSCs quiescence, preventing developmental exhaustion and sustaining continuous neurogenesis throughout adulthood (Zhou et al. 2018). *IGF* signaling is a key regulator of aging, metabolism, reproduction, and growth, and their evolution is conserved across various species (Menting et al. 2013; Weigelt et al. 2020; Szwed et al. 2021). *PTN* influences neuronal differentiation and the maintenance of neuronal plasticity, especially within hippocampal circuits (Ballesteros-Pla et al. 2023). *FGF* is critical for neurodevelopment, including the regulation of RGLs proliferation, neuronal differentiation, and synaptic plasticity, which additionally supports oligodendrocyte lineage progression (Mason 2007). Beyond its role in angiogenesis, *VEGF* promotes neurogenesis and provides neuroprotection by supporting RGLs survival and enhancing oligodendrocyte function (Mackenzie and Ruhrberg 2012). These conserved signaling pathways emphasize their fundamental roles in neural development, plasticity, and repair. Their shared involvement across humans, macaques, and TS highlights their evolutionary importance in maintaining the cellular functions of RGLs, neurons, and oligodendrocytes. Besides, we also identified several species-specific signaling pathways. Specifically, human-specific pathways include *PARs* (Steinhoff et al. 2005)*, GALECTIN* (Wang et al. 2023)*, IL16,* and *CHEMERIN* (Laffranchi et al. 2024). These genes are predominantly involved in neuroendocrine immune regulation, inflammation, and tissue remodeling. In macaques, we identified *AVP* (Pagani et al. 2015) and *CCK* (Dinan and Cryan 2012), which are associated with closely associated with neural regulation, social behavior, and stress responses, indicating potential roles in primate-specific social behavior and stress regulation. Pathways, such as *CXCL, MIF, ENHO, VIP, NPY,* and *ACTIVIN,* reflect inflammatory responses and vascular regulation, which are highly relevant in rodent models but differ in other species. TS-specific signaling pathways, including *NTS* (Christou et al. 2020), *CCL6* (Mantovani et al. 2004)*, IL6* (Hunter and Jones 2015)*, RESISTIN* (Steppan et al. 2001), and *SOMATOSTAIN* (Patel 1999), point to unique aspects of inflammatory regulation, metabolic signaling, and neural communication in TS. The identification of these TS-specific signaling pathways underscores their unique roles in the regulation of inflammation, metabolism, and neuroendocrine interactions. Further exploration of these pathways could provide deeper insights into TS physiology and uncover potential targets for therapeutic intervention. Our findings highlight that TS hippocampus is evolutionarily closer to macaques and humans than to rodents, while it also exhibits unique transcriptomic and cellular features. These results provide a deeper understanding of cross-species hippocampal biology and position TS as a promising model for studying complex human diseases.

The adult mammalian brain contains two primary reservoirs of regenerative NSCs (known as “neurogenic niches”): SVZ of the lateral ventricles and the DG of the hippocampus (Navarro Negredo et al. 2020). In vivo labeling and microscopy revealed a decline in neurogenesis in the SVZ and hippocampal neurogenic niches during aging and AD (Cope and Gould 2019; Tobin et al. 2019; Navarro Negredo et al. 2020; Yu et al. 2024). This decline likely involves a number of cellular processes, including increased NSCs dormancy, decreased NSCs self-renewal, decline in neuronal fate commitment, and NSCs aging or death (Dong et al. 2017; Navarro Negredo et al. 2020; Yan et al. 2023). In this study, we observed a decreasing trend in the number of RGLs with age, along with a decline in their ability to differentiate into new neurons. More importantly, significant remodeling of the transcriptomes of various cell types was identified, indicating dysregulation at multiple stages of the developmental trajectories of neurogenic lineage cells in the aged non-human primate hippocampus. These disruptions were observed at both the early stages of neurogenesis and the later stages involving synaptic transmission. Specifically, the transcriptional regulatory network analysis revealed that *TCF7L1* and *TCF7L2* are central TFs during the aging of NSCs and Astro in the TS hippocampus. The genes targeted by *TCF7L1* and *TCF7L2* are associated with the negative regulation of neurogenesis. Using snRNA-seq and cross-species analysis, we identified various NSCs that are hallmarks of adult hippocampal neurogenesis. The presence of quiescent RGLs and the actively proliferating IPCs provides strong support for the robustness of TS hippocampal neurogenesis. Notably, a unique NSCs population (TS_RGLs) was identified in the TS hippocampus, characterized by markers including *SOXD* (*SOX5* and *SOX6*), *ADAMTS19*, *MAP2*, *CDK6*, *WIF1*, and *SLIT2*, which was further confirmed by immunofluorescent staining. The expression of *SOXD* was enriched in activated RGLs and is a key mediator in the transition of adult RGLs from quiescence to an activated mitotic state under physiological conditions (Li et al. 2022). Meanwhile, *SLIT2,* an extracellular matrix protein, regulates the axonal migration during CNS development (Sherchan et al. 2020).

Moreover, neurogenic niches are specialized microenvironments comprising a variety of different cell types, including cells from the NSCs lineage, endothelial cells, and Micro. An elevated proinflammatory response was observed in the aged Micro, MOL, and Endo, which may trigger the formation of an aging microenvironment in the TS hippocampus. MOL and Endo exacerbate Micro-mediated neuroinflammation via *CD74* and *NOTCH* signaling pathways. In addition, we observed elevated oligodendrocyte proliferation in aged TS hippocampus. The myelin pieces were gradually released from aging myelin sheaths and subsequently cleared by Micro. Myelin fragmentation increases with age and leads to the formation of insoluble, lipofuscin-like lysosomal inclusions in Micro (Safaiyan et al. 2016; Guan et al. 2022). Additionally, senescent Micro show impaired phagocytic function and altered lipid metabolism, which causes the accumulation of lipid metabolites and eventually leads to myelin sheath degeneration (Ahn et al. 2022). Therefore, we provide strong evidence for disrupted hippocampal neurogenesis along with abnormal neuroinflammation modulation in TS during aging.

Altogether, we have generated, to the best of our knowledge, the first comprehensive cellular atlas of the TS hippocampus across the lifespan, from infancy to old age, representing an important species for neuroscience and evolutionary studies. We systematically examined the gene expression profiles and molecular characteristics of each cell type and performed cross-species comparisons at the single-cell level. Our analyses offer rich resources for understanding phenotypic changes associated with aging and the underlying mechanisms of hippocampal aging, potentially paving the way for the development of novel diagnostic and therapeutic approaches for age-related neurodegenerative diseases. One of the limitations of our study is the potential bias introduced by differences in the definition of age across species. Although we made efforts to control for physiological age and sex effects, the comparative equivalence of age between species remains a challenge. For example, the correlation between the age of mice and humans is debated, and this disparity in physiological age could influence the interpretation of cross-species comparisons. Additionally, while we matched for sex in our analysis, different species may have varying gene expression responses to sex, which means that sex-related differences may still exist even in sex-matched samples. These factors could introduce confounding effects in our results and necessitate further research to fully understand the role of age and sex in the transcriptional profiles of hippocampal cells across species. To address these limitations, future studies would benefit from more comprehensive multiomics approaches and experimental validation to explore and mitigate these confounding variables. Moreover, although we used orthologous gene mapping to integrate datasets and minimize species-specific bias, we recognize that discrepancies between datasets in terms of experimental conditions and protocols may influence the comparability of the results. To mitigate these limitations, further rigorous statistical methods should be innovated to adjust for potential differences in data processing across species. More reliable analysis methods are essential for checking the robustness of results and to ensure that the observed cross-species comparisons were not driven by technical artifacts.

## Materials and Methods

### Ethical Statement and TS Sources

Infant (2 to 3 months old, *n* = 5, two males and three females), adult (2 to 3 years old, *n* = 5, two males and three females), and old (5 to 6 years old, *n* = 7, four males and three females) TS were provided from the Animal Center of Kunming Medical University (no. SYXK(Dian) K2020-0004)) and housed in individual cages under a 12-h light/dark cycle, with food and water available throughout the study. We selected TS at different postnatal stages to capture key developmental and aging characteristics based on the published study by Lu et al. (2018). All experimental procedures were approved by the Experimental Animal Ethics Committee of Kunming Medical University (no. KMMU2020001) and were conducted in compliance with *Guide for the Care and Use of Laboratory Animals* published by the National Institutes of Health.

### Sample Collection and Nucleus Extraction

The hippocampus was carefully dissected from the TS with strict compliance to the ethical guidelines. The dissected tissues were washed with cold phosphate-buffered saline (PBS; Invitrogen, Carlsbad, CA, USA), quickly frozen, and then stored in liquid nitrogen before use. Prior to the library construction process, the tissues were first thawed, cut into small pieces, and then transferred to a 1.5-mL tube containing 1× homogenization buffer containing 30 mmol/L CaCl_2_, 18 mmol/L Mg(Ac)_2_, 60 mmol/L Tris–HCl (pH 7.8), 320 mmol/L sucrose, 0.1% nonidet P-40, and 0.1 mmol/L ethylenediaminetetraacetic acid (Invitrogen). The tissue pieces were then transferred to a 2-mL Dounce homogenizer and stroked on ice with loose pestles and then with tight pestles 15 times. The nucleus extraction was filtered with a 40-mm strainer and spanned down at a speed of 500 × *g* for 10 min at 4 °C to carefully discard the supernatant. The pellets were resuspended in PBS containing 0.1% bovine serum albumin (Invitrogen) and 20 U/mL RNase inhibitor for 10 × Genomics library construction (10 × Genomics, Pleasanton, CA, USA).

### Sequencing Libraries Construction

SnRNA-seq was performed using the 10 × Genomics Chromium platform with Chromium Controller Readiness Test to ensure optimal performance. Samples were processed with the 10 × Genomics Chromium Single Cell Kit v.3. The single-cell suspensions were prepared at a concentration of 1,000 cells per μL in 0.04% PBS–bovine serum albumin to maintain cell viability and minimize aggregation. The Chromium RT mix was added to each sample, and the single-cell capture was performed following 10 × Genomics' guidelines. Key parameters, such as the version of the kit, cell viability, initial cell stock concentration, and total cell stock volume, were carefully monitored to achieve a target capture of 5,000 to 10,000 cells per sample. The processes included cell barcoding and reverse transcription, followed by cDNA synthesis (12 to 14 PCR cycles, depending on the quality and yield of the cDNA) and library preparation. Quality control was rigorously performed according to the manufacturer's instructions to ensure high-quality libraries. Libraries were sequenced on the Illumina HiSeq 4000 platform (Hao et al. 2022) to generate sufficient read depth for comprehensive transcriptomic analysis.

### Preprocessing and Quality Control of snRNA-Seq Data

Raw sequencing data were processed using Cell Ranger software (v.7.0.1, 10 × Genomics) to align reads to the *T. belangeri* TS_3.0 genome (available at http://www.treeshrewdb.org/download.html). Cell-by-gene count matrices were generated following sequence alignment with Cell Ranger software (https://support.10xgenomics.com/), and stringent quality control measures were applied to ensure data reliability. We only retained cells with 200 to 6,000 detected genes and the percentage of the detected mitochondrial genes <2% of total gene expression to minimize artifacts from low-quality or stressed cells. To identify and remove potential doublets, we employed DoubletFinder (v.2.0.3), setting the expected doublet rate at 0.075. Genes expressed in fewer than three nuclei were excluded. Ribosomal RNA content was restricted to <5%, and cells with erythroid gene expression (*HBA1, HBA2, HBB, HBD, HBE1, HBG1, HBG2, HBM, HBQ1, HBZ*) >0.2% were also removed to minimize contamination from erythroid RNA. After sample integration and clustering, clusters lacking specific cell-type marker genes (expressing markers of more than one cell type) with relatively low gene content or disproportionately high mitochondrial gene expression were discarded.

### Identification of Cell Clusters

After filtering, unsupervised clustering was performed using Seurat v4 (https://satijalab.org/seurat/) (Butler et al. 2018). Datasets from different sequencing libraries underwent normalization (using “NormalizeData()” function with parameters “normalization.method = “LogNormalize, “scale.factor = 10000”) and identification of highly variable genes (HVGs) (using “FindVariableFeatures()” function with the options “selection.method = “vst, “nfeatures = 2000”). Then, we applied the “FindIntegrationAnchors” and “IntegrateData” functions to integrate all sequencing libraries with the top 20 significant principal components (PCs) (dim = 1:50). The top 2,000 HVGs of each dataset were used for downstream PC analysis (PCA). The top 30 significant PCs were selected for clustering and visualization using UMAP. We removed batch effects and preserved the biological variation present in our dataset by conducting a canonical correction analysis among individual samples with the functions “FindIntegrationAnchors” and “IntegrateData.” Clustering analysis was performed with the functions “FindNeighbors” and “FindClusters.”

### Identification of DEGs Across Cell Type

To identify genes differentially expressed in each group per cell type, *P*-values were calculated and false discovery rate (FDR)-corrected using MAST (Schirmer et al. 2019). All nuclei from infancy, adult, and aging samples for corresponding cell types were used. MAST was used to perform zero-inflated regression analysis by fitting a linear mixed model. To exclude gene expression changes stemming from confounders, such as sex, fractions of ribosomal and mitochondrial transcripts, the following model was fit with MAST:

zlm(∼condition + nCount_RNA + percent.mt + percent.rb + Sex, sca, method = glmer, ebayes = T), where percent.rb is the ribosomal RNA fraction and percent.mt is the mitochondrial RNA fraction.

To identify genes differentially expressed due to the age effect, likelihood ratio test was performed by comparing the model with and without the diagnosis factor. Genes with at least 50% increase or decrease in expression in a group vs other group and an FDR-corrected *P* < 0.05 were selected as differentially expressed.

### GO Term Enrichment Analysis

The “enrichGO()” function of cluster Profiler R package (Yu et al. 2012) was used for enrichment analysis, and the Benjamini-and-Hochberg (BH) method was employed for multiple test correction (OrgDb = org.Hs.eg.db, pAdjustMethod = “BH,” pvalueCutoff = 0.05). A GO term with an adjusted *P*-value of <0.05 was considered significantly enriched. Notably, we used the “org.Hs.eg.db” package because of the homogeneity between TS and humans and the lack of comprehensive TS resources.

### Gene Set Enrichment Analysis

GSEA was applied to identify a priori defined gene sets that showed statistically significant differences between two given clusters. We used the expression file as input and implied gene sets of KEGG pathways and Gene Ontology, which were collected in the Molecular Signatures Database (Zhong et al. 2018).

### Construction of Cellular Communication Network

Intercellular communication analysis was conducted using the CellChat (v.0.0.1) R package with default parameters. TS hippocampus datasets for infant, adult, and old were analyzed separately. Intercellular communication analysis was performed based on cell types. The cell–cell communication network was visualized using the “netVisual_aggregate” function, centrality score was computed and visualized using the “netAnalysis_signalingRole_network” function, and relative contribution of each ligand-receptor pair was visualized using the “netAnalysis_contribution” function.

### Transcriptional Noise Analysis

Transcriptional noise was estimated as described previously (Angelidis et al. 2019; Zhang et al. 2021). In brief, equal numbers of cells of each cell type were used among the young, adult, and old groups. All genes were ordered according to their expression levels, and those with the top 10% and bottom 10% expression levels were excluded. Transcriptional noise at the cell level was then calculated as the Euclidean distances between cells with the mean value of the corresponding cell type.

### Gene Set Score Analysis

Gene sets related to aging-related diseases were obtained from the DisGeNET database (https://www.disgenet.org/home/). Gene sets for AD and PD were generated by filtering with “disease Name” (“Alzheimer's disease” and “Parkinson disease,” respectively). The gene set of Learning Disorders consists of genes related to “Learning Disorders,” “Learning Disturbance,” and “Learning Disabilities.” Genes related to human brain aging, SASP, and HuMi_Aged were acquired from literature (Lu et al. 2004; Olah et al. 2018; Zhang et al. 2021). Gene set scores were acquired by analyzing the transcriptome of each input cell against the aforementioned gene sets using the Seurat function “AddModuleScore.” Changes in the scores between young, adult, and old samples were analyzed using the ggpubr R package via the Wilcoxon test.

### Cross-Species Comparison

We performed a cross-species comparison by integrating previously reported snRNA-seq datasets of mice (Hochgerner et al. 2018), macaques (Wang et al. 2022b), and humans (Wang et al. 2022b) hippocampus with TS hippocampus snRNA-seq dataset generated in this study. Prior to performing species integration analysis, we conducted individual quality control, dimensionality reduction, and cell annotation for the snRNA-seq data of each species. To ensure high-quality snRNA-seq data across species, we applied a consistent quality control pipeline with species-specific adjustments. Reads were aligned to the respective reference genomes (GRCh38 for humans, Macaca_fascicularis_6.0 for macaques, and mm10 for mice) using Cell Ranger to generate cell-by-gene count matrices, and DoubletFinder with doublet rate set at 0.075 was used to identify doublets for each sample individually. For all species, we excluded genes expressed in fewer than three nuclei, restricted ribosomal RNA content to <5%, and removed cells with erythroid gene expression exceeding 0.25% to minimize contamination. Additionally, we retained cells based on species-specific thresholds: for human and mouse datasets, cells with 200 to 6,000 detected genes were kept, while for macaques, the range was 200 to 8,000. Mitochondrial gene expression thresholds were also species-specific: <5% of total expression for humans and macaques and <10% for mice, to account for differences in mitochondrial activity and stress responses. Then, for cross-species integration, orthologous gene sets were determined using human genes as the reference and converted to other species using HomoloGene and biomaRt (Wang et al. 2022b). We focused exclusively on one-to-one orthologous genes to ensure accuracy in cross-species comparisons and excluded genes lacking orthologous matches in one or more species. After identifying the orthologous genes across different species, the integrated analysis was performed using the Seurat v3 pipeline (Stuart et al. 2019). Batch effects and technical variability were addressed using the “Harmony” package to ensure robust integration. In detail, we used “SelectIntegrationFeatures()” (nfeatures = 3,000) function to select the top 3,000 HVGs as integration features, followed by “FindIntegrationAnchors()” (anchor.features = 2,000, dims = 1:50) to identify anchor genes across species. Finally, the datasets were integrated using the “IntegrateData()” (dims = 1:50) function. After integration, the data were normalized, scaled, and centered by using the “ScaleData()” function, followed by dimensionality reduction by the “RunPCA()” function (npcs = 30). Unsupervised clustering was performed to identify cellular subpopulations. Cell identities were annotated based on the expression of canonical cell-type markers validated across species, ensuring consistency in cell-type definitions. Conserved cell types were identified based on shared markers between TS and humans and further validated by reference-based annotation in macaques and mice. Cluster-specific DEGs from TS were mapped back to human orthologs to determine conserved biological processes, pathways, and interspecies similarities.

When performing overall correlation analysis, expression-weighted cell-type enrichment (ECWE) analysis, ToomanyCells analysis, scDRS analysis, and CellChat analysis, we used age- and sex-matched snRNA-seq data from four species: humans, macaques, TS, and mice. This approach minimized confounding effects related to age and sex differences, while enhancing the comparability of cross-species results and improving statistical power. In cross-species analysis of individual cell types, we utilized snRNA-seq data from human, macaque, mouse, and pig samples. Since we primarily focused on the transcriptomic profiles and functional similarities and differences of a specific cell type across species, the effects of age and sex were disregarded.

### EWCE Analysis

EWCE (Skene and Grant 2016; Zhu et al. 2021) was employed for the analysis of disease gene enrichment using default parameters. This analysis was conducted separately for each species dataset. This analysis was performed separately for each species dataset. To mitigate the bias effect, we initially employ the “vst()” function to conduct a variance-stabilizing transformation on the unique molecular identifier count matrix. Subsequently, we utilize the “fix_bad_mgi_symbols()” function to rectify gene nomenclature. The “drop_uninformative_genes()” function is then applied to eliminate noninformative genes, thereby reducing computational time and minimizing noise in subsequent analyses. Following this, we implement the “generate.celltype.data()” function to compute the specificity matrix for each dataset. We perform 100 bootstrap resamplings with replacement on all detected genes within each species dataset as a background reference, and *P*-values are adjusted using the BH method. A significance threshold of 0.05 is established.

### Single-Cell Disease Relevance Score

The single-cell disease relevance score (scDRS) method was employed to assess the polygenic disease enrichment of individual cells within scRNA-seq data (Niu et al. 2024). Initially, to establish statistical significance, scDRS generates 1,000 sets of cell-specific raw control scores through Monte Carlo sampling of matched control gene sets that are aligned in terms of gene set size, mean expression, and expression variance with respect to the candidate disease genes. Subsequently, scDRS normalizes both the raw disease scores and the raw control scores for each cell—resulting in normalized disease scores and normalized control scores—and computes cell-level *P*-values based on the empirical distribution derived from pooled normalized control scores across all control gene sets and all cells.

### Cell Composition Analysis

To quantify the impact of aging on cell differentiation dynamics, we employed two distinct analytical methods for intergroup cell composition difference analysis. These included a cell composition analysis utilizing the Cacoa package (Batiuk et al. 2022) and a differential cell density analysis conducted with the miloR package (Dann et al. 2022). The “estimateCellLoadings()” function from the Cacoa package (v.0.4.0) was utilized for analyzing cellular components. Specifically, logarithmic ratio transformation was applied to the fractions of different cell types, followed by typical discriminant analysis using the candisc software package to derive weighted comparisons between the two sample groups. The separation coefficient was assessed through random subsampling of cells, and its robustness along with statistical significance was thoroughly evaluated. Then, we perform 1,000 resamplings, during which 1,000 cells are randomly selected from each group to assess the robustness of the test. The BH procedure is employed for multiple comparison correction. The Cacoa package's estimateCellDensity function is utilized to conduct a differential cell density analysis. To assess the differences in cell density between sample groups, we first employ the ks R package to compute the kernel density for each sample within the joint embedding space. Subsequently, we normalize the resulting density matrix across samples using quantile normalization techniques. To quantify the differences in cell density between sample groups, we perform a *t*-test on samples located within each grid box. To mitigate background noise, we filtered out boxes containing at least one cell, and the z-scores were represented as a heatmap. The sample labels were randomly shuffled 200 times to assess the robustness of the test. For single-cell differential abundance analysis, we utilized the Milo function to create a miloR object, followed by employing the buildGraph function to construct a K-nearest neighbor graph in UMAP space. Subsequently, the makeNhoods function was applied to define cellular neighborhoods, while the countCells function quantified the number of cells within each neighborhood across all samples. The testNhood function was employed to evaluate neighborhood differential abundance with a spatial FDR significance threshold set at 0.05. Visualization of differential abundance neighborhoods was achieved using the plotNhoodGraphDA function.

### Trajectory Analysis

For cell developmental trajectory assessment, the data for cells belonging to the corresponding cell population were used to create a separate Seurat object using the “SubsetData” function. The most variable genes for these clusters alone were identified using the FindVariableGenes function and the following parameters: x.low.cutoff = 0.003, x.high.cutoff = 3, and y.cutoff = 1. The Seurat object was imported into a CDS (CellDataSet) object using the Monocle function *importCDS* (Trapnell et al. 2014).

### Single-Cell Regulatory Network Inference and Clustering

To carry out TF network inference, data were subsampled by randomly selecting cells from each cell type. Analysis was performed as described using the single-cell regulatory network inference and clustering (SCENIC) R package (v.1.1.0, which corresponds to RcisTarget 1.2.0 and AUCell 1.4.1). The activity of the regulatory networks was evaluated on the full dataset in the scoring step with AUCell. Regulons annotated as “extended” include target genes harboring motifs that have been linked to the respective TF by lower confidence annotations (Aibar et al. 2017).

### Immunofluorescence Staining

Hippocampal tissues from TS, mice, and macaques at different life stages were dissected and fixed with 4% paraformaldehyde for up to 24 h and cryoprotected in 30% sucrose at 4 °C for 72 h. The tissue samples were frozen in optimal cutting temperature compound (Tissue-Tek) at −80 °C and sectioned at 15 µm on a cryostat microtome (Leica CM1950). Sections were rinsed with PBS and incubated for 30 min in 0.3% Triton X-100 (Sigma-Aldrich) and then for 2 h in 5% donkey serum (Vector Laboratories). Subsequently, sections were incubated overnight at 4 °C with the primary antibodies and for 2 h at room temperature with the secondary antibodies. Detailed antibody information is provided in supplementary table S15, Supplementary Material online. Sections were mounted with DAPI (Abcam) and covered with coverslips. Images were obtained using an LSM880 Zeiss microscope and a two-photon confocal microscope (NIS-Elements AX).

### Microglia Tracing and Sholl Analysis

For each microglia, the figure was imported into Fiji (ImageJ, v.2.3.0) and converted into 8-bit images. The z-project feature in Fiji was used to flatten the z-stacks and create the maximum 2D projection image. A skeleton image was generated for each RGC in 2D with the Simple Neurite Tracer plugin, which provides a semiautomated system of tracing over each dendrite in the z-stack. 2D Sholl analysis was performed with the Sholl plugin, which overlays concentric rings 20 microns apart and quantifies the number of skeleton intersections with each ring. The output data were used to generate 2D Sholl profiles, from which the branches, junction, and radiation areas were calculated.

### Mfuzz Analysis

Through the cluster analysis of expression patterns, the gene expression trend of infant, adult, and old TS hippocampus samples was presented.

### Quantification and Statistical Analysis

All data obtained from TS at each age gradient were collected from one sample with at least three independent experiments. Error bars represent SD. Samples from each group were tested for normality and homogeneity before statistical analysis. Statistical analyses were performed using one-way ANOVA with corresponding post hoc tests for multiple group comparison using SPSS Statistics 26.0 (IBM, Chicago, IL, USA). Graphical visualization was performed using GraphPad Prism software (v.9.1.1). The sample size and *P*-values are given in the figure legends. *P*-values were designated as follows: *P ≤ 0.05, **P ≤ 0.01, ***P ≤ 0.001, and ****P ≤ 0.0001.

## Supplementary Material

msaf020_Supplementary_Data

## Data Availability

The raw sequence data reported in this paper have been deposited in the Gene Expression Ominibus of National Center for Biotechnology Information under accession number GSE229035 and are accessible at https://www.ncbi.nlm.nih.gov/geo/query/acc.cgi?acc=GSE229035.

## References

[msaf020-B1] Aguirre A, Rubio ME, Gallo V. Notch and EGFR pathway interaction regulates neural stem cell number and self-renewal. Nature. 2010:467(7313):323–327. 10.1038/nature09347. 20844536 PMC2941915

[msaf020-B2] Ahn K, Lee SJ, Mook-Jung I. White matter-associated microglia: new players in brain aging and neurodegenerative diseases. Ageing Res Rev. 2022:75:101574. 10.1016/j.arr.2022.101574. 35093614

[msaf020-B3] Aibar S, González-Blas CB, Moerman T, Huynh-Thu VA, Imrichova H, Hulselmans G, Rambow F, Marine JC, Geurts P, Aerts J, et al SCENIC: single-cell regulatory network inference and clustering. Nat Methods. 2017:14(11):1083–1086. 10.1038/nmeth.4463. 28991892 PMC5937676

[msaf020-B4] Allen NJ, Lyons DA. Glia as architects of central nervous system formation and function. Science. 2018:362(6411):181–185. 10.1126/science.aat0473. 30309945 PMC6292669

[msaf020-B5] Angelidis I, Simon LM, Fernandez IE, Strunz M, Mayr CH, Greiffo FR, Tsitsiridis G, Ansari M, Graf E, Strom TM, et al An atlas of the aging lung mapped by single cell transcriptomics and deep tissue proteomics. Nat Commun. 2019:10(1):963. 10.1038/s41467-019-08831-9. 30814501 PMC6393476

[msaf020-B6] Athapaththu AMGK, Molagoda IMN, Jayasooriya R, Choi YH, Jeon YJ, Park JH, Lee BJ, Kim GY. Gamma-aminobutyric acid (GABA) promotes growth in zebrafish larvae by inducing IGF-1 expression via GABA(A) and GABA(B) receptors. Int J Mol Sci. 2021:22(20):11254. 10.3390/ijms222011254. 34681914 PMC8537617

[msaf020-B7] Ballesteros-Pla C, Sánchez-Alonso MG, Pizarro-Delgado J, Zuccaro A, Sevillano J, Ramos-Álvarez MP. Pleiotrophin and metabolic disorders: insights into its role in metabolism. Front Endocrinol (Lausanne). 2023:14:1225150. 10.3389/fendo.2023.1225150. 37484951 PMC10360176

[msaf020-B8] Batiuk MY, Tyler T, Dragicevic K, Mei S, Rydbirk R, Petukhov V, Deviatiiarov R, Sedmak D, Frank E, Feher V, et al Upper cortical layer-driven network impairment in schizophrenia. Sci Adv. 2022:8(41):eabn8367. 10.1126/sciadv.abn8367. PMC9555788 36223459

[msaf020-B9] Boldog E, Bakken TE, Hodge RD, Novotny M, Aevermann BD, Baka J, Bordé S, Close JL, Diez-Fuertes F, Ding SL, et al Transcriptomic and morphophysiological evidence for a specialized human cortical GABAergic cell type. Nat Neurosci. 2018:21(9):1185–1195. 10.1038/s41593-018-0205-2. 30150662 PMC6130849

[msaf020-B10] Butler A, Hoffman P, Smibert P, Papalexi E, Satija R. Integrating single-cell transcriptomic data across different conditions, technologies, and species. Nat Biotechnol. 2018:36(5):411–420. 10.1038/nbt.4096. 29608179 PMC6700744

[msaf020-B11] Christou N, Blondy S, David V, Verdier M, Lalloué F, Jauberteau MO, Mathonnet M, Perraud A. Neurotensin pathway in digestive cancers and clinical applications: an overview. Cell Death Dis. 2020:11(12):1027. 10.1038/s41419-020-03245-8 33268796 PMC7710720

[msaf020-B12] Colgan SP, Taylor CT. Hypoxia: an alarm signal during intestinal inflammation. Nat Rev Gastroenterol Hepatol. 2010:7(5):281 287. doi:10.1038/nrgastro.2010.39. 20368740 PMC4077542

[msaf020-B13] Cope EC, Gould E. Adult neurogenesis, glia, and the extracellular matrix. Cell Stem Cell. 2019:24(5):690–705. 10.1016/j.stem.2019.03.023. 31051133 PMC7961263

[msaf020-B14] Dai JK, Wang SX, Shan D, Niu HC, Lei H. A diffusion tensor imaging atlas of white matter in tree shrew. Brain Struct Funct. 2017:222(4):1733–1751. 10.1007/s00429-016-1304-z. 27624528

[msaf020-B15] Dann E, Henderson NC, Teikichmann SA, Morgan MD, Marioni JC. Differential abundance testing on single-cell data using k-nearest neighbor graphs. Nat Biotechnol. 2022:40(2):245–253. 10.1038/s41587-021-01033-z. 34594043 PMC7617075

[msaf020-B16] Deczkowska A, Matcovitch-Natan O, Tsitsou-Kampeli A, Ben-Hamo S, Dvir-Szternfeld R, Spinrad A, Singer O, David E, Winter DR, Smith LK, et al Mef2C restrains microglial inflammatory response and is lost in brain ageing in an IFN-I-dependent manner. Nat Commun. 2017:8(1):717. 10.1038/s41467-017-00769-0. 28959042 PMC5620041

[msaf020-B17] Dinan TG, Cryan JF. Regulation of the stress response by the gut microbiota: implications for psychoneuroendocrinology. Psychoneuroendocrinology. 2012:37(9):1369–1378. 10.1016/j.psyneuen.2012.03.007. 22483040

[msaf020-B18] Dong CM, Wang XL, Wang GM, Zhang WJ, Zhu L, Gao S, Yang DJ, Qin Y, Liang QJ, Chen YL, et al A stress-induced cellular aging model with postnatal neural stem cells. Cell Death Dis. 2017:8(9):e3041. 10.1038/cddis.2017.445. 28880269 PMC5636989

[msaf020-B19] Fan Y, Huang ZY, Cao CC, Chen CS, Chen YX, Fan DD, He J, Hou HL, Hu L, Hu XT, et al Genome of the Chinese tree shrew. Nat Commun. 2013:4(1):1426. 10.1038/ncomms2416. 23385571

[msaf020-B20] Fan Y, Ye MS, Zhang JY, Xu L, Yu DD, Gu TL, Yao YL, Chen JQ, Lv LB, Zheng P, et al Chromosomal level assembly and population sequencing of the Chinese tree shrew genome. Zool Res. 2019:40(6):506–521. 10.24272/j.issn.2095-8137.2019.063. 31418539 PMC6822927

[msaf020-B21] Faye C, McGowan JC, Denny CA, David DJ. Neurobiological mechanisms of stress resilience and implications for the aged population. Curr Neuropharmacol. 2018:16(3):234–270. 10.2174/1570159X15666170818095105. 28820053 PMC5843978

[msaf020-B22] Fernandes VM, Chen Z, Rossi AM, Zipfel J, Desplan C. Glia relay differentiation cues to coordinate neuronal development in *Drosophila*. Science. 2017:357(6354):886–891. 10.1126/science.aan3174. 28860380 PMC5835562

[msaf020-B23] Franjic D, Skarica M, Ma S, Arellano JI, Tebbenkamp ATN, Choi J, Xu C, Li Q, Morozov YM, Andrijevic D, et al Transcriptomic taxonomy and neurogenic trajectories of adult human, macaque, and pig hippocampal and entorhinal cells. Neuron. 2022:110(3):452–469.e14. 10.1016/j.neuron.2021.10.036. 34798047 PMC8813897

[msaf020-B24] García-Jiménez MJ, Torres-Rico M, de Paz JL, Nieto PM. The interaction between chondroitin sulfate and dermatan sulfate tetrasaccharides and pleiotrophin. Int J Mol Sci. 2022:23(6):3026. 10.3390/ijms23063026. 35328448 PMC8955691

[msaf020-B25] Gómez-Gaviro MV, Scott CE, Sesay AK, Matheu A, Booth S, Galichet C, Lovell-Badge R. Betacellulin promotes cell proliferation in the neural stem cell niche and stimulates neurogenesis. Proc Natl Acad Sci U S A. 2012:109(4):1317–1322. 10.1073/pnas.1016199109. 22232668 PMC3268286

[msaf020-B26] Guan Y-H, Zhang L-J, Wang S-Y, Deng Y-D, Zhou H-S, Chen D-Q, Zhang L-C. The role of microglia in Alzheimer’s disease and progress of treatment. Ibrain. 2022:8(1):37–47. 10.1002/ibra.12023. 37786418 PMC10529349

[msaf020-B27] Guo H, Miao L, Zhou F. Type-I-interferon-responsive microglia: participates in cerebral development and disease. MedComm (2020). 2024:5(7):e629. 10.1002/mco2.629. 38974712 PMC11225717

[msaf020-B28] Hansen CE, Vacondio D, van der Molen L, Jüttner AA, Fung WK, Karsten M, van Het Hof B, Fontijn RD, Kooij G, Witte ME, et al Endothelial-Ercc1 DNA repair deficiency provokes blood-brain barrier dysfunction. Cell Death Dis. 2025:16(1):1. 10.1038/s41419-024-07306-0. 39753531 PMC11698980

[msaf020-B29] Hao ZZ, Wei JR, Xiao D, Liu R, Xu N, Tang L, Huang M, Shen Y, Xing C, Huang W, et al Single-cell transcriptomics of adult macaque hippocampus reveals neural precursor cell populations. Nat Neurosci. 2022:25(6):805–817. 10.1038/s41593-022-01073-x. 35637371

[msaf020-B30] Heller AS, Shi TC, Ezie CEC, Reneau TR, Baez LM, Gibbons CJ, Hartley CA. Association between real-world experiential diversity and positive affect relates to hippocampal-striatal functional connectivity. Nat Neurosci. 2020:23(7):800–804. 10.1038/s41593-020-0636-4. 32424287 PMC9169417

[msaf020-B31] Hochgerner H, Zeisel A, Lönnerberg P, Linnarsson S. Conserved properties of dentate gyrus neurogenesis across postnatal development revealed by single-cell RNA sequencing. Nat Neurosci. 2018:21(2):290–299. 10.1038/s41593-017-0056-2. 29335606

[msaf020-B32] Horowitz AM, Fan X, Bieri G, Smith LK, Sanchez-Diaz CI, Schroer AB, Gontier G, Casaletto KB, Kramer JH, Williams KE, et al Blood factors transfer beneficial effects of exercise on neurogenesis and cognition to the aged brain. Science. 2020:369(6500):167–173. 10.1126/science.aaw2622. 32646997 PMC7879650

[msaf020-B33] Huang Q, Nie B, Ma C, Wang J, Zhang T, Duan S, Wu S, Liang S, Li P, Liu H, et al Stereotaxic (18)F-FDG PET and MRI templates with three-dimensional digital atlas for statistical parametric mapping analysis of tree shrew brain. J Neurosci Methods. 2018:293:105–116. 10.1016/j.jneumeth.2017.09.006. 28917660

[msaf020-B34] Hunt RF, Girskis KM, Rubenstein JL, Alvarez-Buylla A, Baraban SC. GABA progenitors grafted into the adult epileptic brain control seizures and abnormal behavior. Nat Neurosci. 2013:16(6):692–697. 10.1038/nn.3392. 23644485 PMC3665733

[msaf020-B35] Hunter CA, Jones SA. IL-6 as a keystone cytokine in health and disease. Nat Immunol. 2015:16(5):448–457. 10.1038/ni.3153. 25898198

[msaf020-B36] Ivanovska M, Abdi Z, Murdjeva M, Macedo D, Maes A, Maes M. CCL-11 or eotaxin-1: an immune marker for ageing and accelerated ageing in neuro-psychiatric disorders. Pharmaceuticals (Basel). 2020:13(9):230. 10.3390/ph13090230. 32887304 PMC7558796

[msaf020-B37] Jackson M, Song W, Liu MY, Jin L, Dykes-Hoberg M, Lin CI, Bowers WJ, Federoff HJ, Sternweis PC, Rothstein JD. Modulation of the neuronal glutamate transporter EAAT4 by two interacting proteins. Nature. 2001:410(6824):89–93. 10.1038/35065091. 11242047

[msaf020-B38] Jäkel S, Agirre E, Mendanha Falcão A, van Bruggen D, Lee KW, Knuesel I, Malhotra D, Ffrench-Constant C, Williams A, Castelo-Branco G. Altered human oligodendrocyte heterogeneity in multiple sclerosis. Nature. 2019:566(7745):543–547. 10.1038/s41586-019-0903-2. 30747918 PMC6544546

[msaf020-B39] Laffranchi M, Schioppa T, Sozio F, Piserà A, Tiberio L, Salvi V, Bosisio D, Musso T, Sozzani S, Del Prete A. Chemerin in immunity. J Leukoc Biol. 2024: qiae181. 10.1093/jleuko/qiae181. 39228313

[msaf020-B40] Li L, Medina-Menéndez C, García-Corzo L, Córdoba-Beldad CM, Quiroga AC, Calleja Barca E, Zinchuk V, Muñoz-López S, Rodríguez-Martín P, Ciorraga M, et al Soxd genes are required for adult neural stem cell activation. Cell Rep. 2022:38(5):110313. 10.1016/j.celrep.2022.110313. 35108528 PMC11783645

[msaf020-B41] Liebner S, Dijkhuizen RM, Reiss Y, Plate KH, Agalliu D, Constantin G. Functional morphology of the blood-brain barrier in health and disease. Acta Neuropathol. 2018:135(3):311–336. 10.1007/s00401-018-1815-1. 29411111 PMC6781630

[msaf020-B42] Lincoln S, Allen M, Cox CL, Walker LP, Malphrus K, Qiu Y, Nguyen T, Rowley C, Kouri N, Crook J, et al LRRTM3 interacts with APP and BACE1 and has variants associating with late-onset Alzheimer’s disease (LOAD). PLoS One. 2013:8(6):e64164. 10.1371/journal.pone.0064164. 23750206 PMC3672107

[msaf020-B43] Lu C, Sun X, Li N, Wang W, Kuang D, Tong P, Han Y, Dai J. CircRNAs in the tree shrew (*Tupaia belangeri*) brain during postnatal development and aging. Aging (Albany NY). 2018:10(4):833–852. 10.18632/aging.101437. 29723158 PMC5940110

[msaf020-B44] Lu T, Pan Y, Kao SY, Li C, Kohane I, Chan J, Yankner BA. Gene regulation and DNA damage in the ageing human brain. Nature. 2004:429(6994):883–891. 10.1038/nature02661. 15190254

[msaf020-B45] Mackenzie F, Ruhrberg C. Diverse roles for VEGF-A in the nervous system. Development. 2012:139(8):1371–1380. 10.1242/dev.072348. 22434866

[msaf020-B46] Malanchi I, Santamaria-Martínez A, Susanto E, Peng H, Lehr HA, Delaloye JF, Huelsken J. Interactions between cancer stem cells and their niche govern metastatic colonization. Nature. 2011:481(7379):85–89. 10.1038/nature10694. 22158103

[msaf020-B47] Mantovani A, Sica A, Sozzani S, Allavena P, Vecchi A, Locati M. The chemokine system in diverse forms of macrophage activation and polarization. Trends Immunol. 2004:25(12):677–686. 10.1016/j.it.2004.09.015. 15530839

[msaf020-B48] Mason I . Initiation to end point: the multiple roles of fibroblast growth factors in neural development. Nat Rev Neurosci. 2007:8(8):583–596. 10.1038/nrn2189. 17637802

[msaf020-B49] Meng Z, Li FL, Fang C, Yeoman B, Qiu Y, Wang Y, Cai X, Lin KC, Yang D, Luo M, et al The Hippo pathway mediates semaphorin signaling. Sci Adv. 2022:8(21):eabl9806. 10.1126/sciadv.abl9806. PMC9132450 35613278

[msaf020-B50] Menting JG, Whittaker J, Margetts MB, Whittaker LJ, Kong GK, Smith BJ, Watson CJ, Záková L, Kletvíková E, Jiráček J, et al How insulin engages its primary binding site on the insulin receptor. Nature. 2013:493(7431):241–245. 10.1038/nature11781. 23302862 PMC3793637

[msaf020-B51] Miyashita A, Arai H, Asada T, Imagawa M, Matsubara E, Shoji M, Higuchi S, Urakami K, Kakita A, Takahashi H, et al Genetic association of CTNNA3 with late-onset Alzheimer’s disease in females. Hum Mol Genet. 2007:16(23):2854–2869. 10.1093/hmg/ddm244. 17761686

[msaf020-B52] Navarro Negredo P, Yeo RW, Brunet A. Aging and rejuvenation of neural stem cells and their niches. Cell Stem Cell. 2020:27(2):202–223. 10.1016/j.stem.2020.07.002. 32726579 PMC7415725

[msaf020-B53] Niu RZ, Xu HY, Tian H, Zhang D, He CY, Li XL, Li YY, He J. Single-cell transcriptome unveils unique transcriptomic signatures of human organ-specific endothelial cells. Basic Res Cardiol. 2024:119(6):973–999. 10.1007/s00395-024-01087-5. 39508863

[msaf020-B54] Olah M, Patrick E, Villani AC, Xu J, White CC, Ryan KJ, Piehowski P, Kapasi A, Nejad P, Cimpean M, et al A transcriptomic atlas of aged human microglia. Nat Commun. 2018:9(1):539. 10.1038/s41467-018-02926-5. 29416036 PMC5803269

[msaf020-B55] Pagani JH, Zhao M, Cui Z, Avram SK, Caruana DA, Dudek SM, Young WS. Role of the vasopressin 1b receptor in rodent aggressive behavior and synaptic plasticity in hippocampal area CA2. Mol Psychiatry. 2015:20(4):490–499. 10.1038/mp.2014.47. 24863146 PMC4562468

[msaf020-B56] Patel YC . Somatostatin and its receptor family. Front Neuroendocrinol. 1999:20(3):157–198. 10.1006/frne.1999.0183. 10433861

[msaf020-B57] Rummel C . Inflammatory transcription factors as activation markers and functional readouts in immune-to-brain communication. Brain Behav Immun. 2016:54:1–14. 10.1016/j.bbi.2015.09.003. 26348582

[msaf020-B58] Safaiyan S, Kannaiyan N, Snaidero N, Brioschi S, Biber K, Yona S, Edinger AL, Jung S, Rossner MJ, Simons M. Age-related myelin degradation burdens the clearance function of microglia during aging. Nat Neurosci. 2016:19(8):995–998. 10.1038/nn.4325. 27294511 PMC7116794

[msaf020-B59] Schirmer L, Velmeshev D, Holmqvist S, Kaufmann M, Werneburg S, Jung D, Vistnes S, Stockley JH, Young A, Steindel M, et al Neuronal vulnerability and multilineage diversity in multiple sclerosis. Nature. 2019:573(7772):75–82. 10.1038/s41586-019-1404-z. 31316211 PMC6731122

[msaf020-B60] Schmidt MF, Gan ZY, Komander D, Dewson G. Ubiquitin signalling in neurodegeneration: mechanisms and therapeutic opportunities. Cell Death Differ. 2021:28(2):570–590. 10.1038/s41418-020-00706-7. 33414510 PMC7862249

[msaf020-B61] Schwartz GW, Zhou Y, Petrovic J, Fasolino M, Xu L, Shaffer SM, Pear WS, Vahedi G, Faryabi RB. TooManyCells identifies and visualizes relationships of single-cell clades. Nat Methods. 2020:17(4):405–413. 10.1038/s41592-020-0748-5. 32123397 PMC7439807

[msaf020-B62] Sherchan P, Travis ZD, Tang J, Zhang JH. The potential of Slit2 as a therapeutic target for central nervous system disorders. Expert Opin Ther Targets. 2020:24(8):805–818. 10.1080/14728222.2020.1766445. 32378435 PMC7529836

[msaf020-B63] Sideras P, Apostolou E, Stavropoulos A, Sountoulidis A, Gavriil A, Apostolidou A, Andreakos E. Activin, neutrophils, and inflammation: just coincidence? Semin Immunopathol. 2013:35(4):481–499. 10.1007/s00281-013-0365-9. 23385857 PMC7101603

[msaf020-B64] Skene NG, Grant SG. Identification of vulnerable cell types in major brain disorders using single cell transcriptomes and expression weighted cell type enrichment. Front Neurosci. 2016:10:16. 10.3389/fnins.2016.00016. 26858593 PMC4730103

[msaf020-B65] Sorrells SF, Paredes MF, Cebrian-Silla A, Sandoval K, Qi D, Kelley KW, James D, Mayer S, Chang J, Auguste KI, et al Human hippocampal neurogenesis drops sharply in children to undetectable levels in adults. Nature. 2018:555(7696):377–381. 10.1038/nature25975. 29513649 PMC6179355

[msaf020-B66] Steinhoff M, Buddenkotte J, Shpacovitch V, Rattenholl A, Moormann C, Vergnolle N, Luger TA, Hollenberg MD. Proteinase-activated receptors: transducers of proteinase-mediated signaling in inflammation and immune response. Endocr Rev. 2005:26(1):1–43. 10.1210/er.2003-0025. 15689571

[msaf020-B67] Steppan CM, Bailey ST, Bhat S, Brown EJ, Banerjee RR, Wright CM, Patel HR, Ahima RS, Lazar MA. The hormone resistin links obesity to diabetes. Nature. 2001:409(6818):307–312. 10.1038/35053000. 11201732

[msaf020-B68] Stuart T, Butler A, Hoffman P, Hafemeister C, Papalexi E, Mauck WM, 3rd, Hao Y, Stoeckius M, Smibert P, Satija R. Comprehensive integration of single-cell data. Cell. 2019:177(7):1888–1902.e21. 10.1016/j.cell.2019.05.031. 31178118 PMC6687398

[msaf020-B69] Sultan S, Li L, Moss J, Petrelli F, Cassé F, Gebara E, Lopatar J, Pfrieger FW, Bezzi P, Bischofberger J, et al Synaptic integration of adult-born hippocampal neurons is locally controlled by astrocytes. Neuron. 2015:88(5):957–972. 10.1016/j.neuron.2015.10.037. 26606999

[msaf020-B70] Szwed A, Kim E, Jacinto E. Regulation and metabolic functions of mTORC1 and mTORC2. Physiol Rev. 2021:101(3):1371–1426. 10.1152/physrev.00026.2020. 33599151 PMC8424549

[msaf020-B71] Tan HL, Chiu SL, Zhu Q, Huganir RL. GRIP1 regulates synaptic plasticity and learning and memory. Proc Natl Acad Sci U S A. 2020:117(40):25085–25091. 10.1073/pnas.2014827117. 32948689 PMC7547244

[msaf020-B72] Tobin MK, Musaraca K, Disouky A, Shetti A, Bheri A, Honer WG, Kim N, Dawe RJ, Bennett DA, Arfanakis K, et al Human hippocampal neurogenesis persists in aged adults and Alzheimer’s disease patients. Cell Stem Cell. 2019:24(6):974–982.e973. 10.1016/j.stem.2019.05.003. 31130513 PMC6608595

[msaf020-B73] Trapnell C, Cacchiarelli D, Grimsby J, Pokharel P, Li S, Morse M, Lennon NJ, Livak KJ, Mikkelsen TS, Rinn JL. The dynamics and regulators of cell fate decisions are revealed by pseudotemporal ordering of single cells. Nat Biotechnol. 2014:32(4):381–386. 10.1038/nbt.2859. 24658644 PMC4122333

[msaf020-B74] Um JW, Pramanik G, Ko JS, Song MY, Lee D, Kim H, Park KS, Südhof TC, Tabuchi K, Ko J. Calsyntenins function as synaptogenic adhesion molecules in concert with neurexins. Cell Rep. 2014:6(6):1096–1109. 10.1016/j.celrep.2014.02.010. 24613359 PMC4101519

[msaf020-B75] Vandenbark AA, Meza-Romero R, Benedek G, Offner H. A novel neurotherapeutic for multiple sclerosis, ischemic injury, methamphetamine addiction, and traumatic brain injury. J Neuroinflammation. 2019:16(1):14. 10.1186/s12974-018-1393-0. 30683115 PMC6346590

[msaf020-B76] van der Klaauw AA, Croizier S, Mendes de Oliveira E, Stadler LKJ, Park S, Kong Y, Banton MC, Tandon P, Hendricks AE, Keogh JM, et al Human semaphorin 3 variants link melanocortin circuit development and energy balance. Cell. 2019:176(4):729–742.e718. 10.1016/j.cell.2018.12.009. 30661757 PMC6370916

[msaf020-B77] Wang F, Ding P, Liang X, Ding X, Brandt CB, Sjöstedt E, Zhu J, Bolund S, Zhang L, de Rooij L, et al Endothelial cell heterogeneity and microglia regulons revealed by a pig cell landscape at single-cell level. Nat Commun. 2022a:13(1):3620. 10.1038/s41467-022-31388-z. 35750885 PMC9232580

[msaf020-B78] Wang W, Wang M, Yang M, Zeng B, Qiu W, Ma Q, Jing X, Zhang Q, Wang B, Yin C, et al Transcriptome dynamics of hippocampal neurogenesis in macaques across the lifespan and aged humans. Cell Res. 2022b:32(8):729–743. 10.1038/s41422-022-00678-y. 35750757 PMC9343414

[msaf020-B79] Wang Y, Chung DH, Monteleone LR, Li J, Chiang Y, Toney MD, Beal PA. RNA binding candidates for human ADAR3 from substrates of a gain of function mutant expressed in neuronal cells. Nucleic Acids Res. 2019a:47(20):10801–10814. 10.1093/nar/gkz815. 31552420 PMC6846710

[msaf020-B80] Wang Y, Sun Y, Li H, Xu J. Galectin-8 alters immune microenvironment and promotes tumor progression. Am J Cancer Res. 2023:13:2517–2529. 37424827 PMC10326578

[msaf020-B81] Wang YY, Niu RZ, Wang JD, Jin Y, Wang TH, Liu F. Establishment of brain ischemia model in tree shrew. Brain Res. 2019b:1718:194–200. 10.1016/j.brainres.2019.05.011. 31077648

[msaf020-B82] Weigelt CM, Sehgal R, Tain LS, Cheng J, Eßer J, Pahl A, Dieterich C, Grönke S, Partridge L. An insulin-sensitive circular RNA that regulates lifespan in *Drosophila*. Mol Cell. 2020:79(2):268–279.e265. 10.1016/j.molcel.2020.06.011. 32592682 PMC7318944

[msaf020-B83] Xia T, Yu J, Du M, Chen X, Wang C, Li R. Vascular endothelial cell injury: causes, molecular mechanisms, and treatments. MedComm (2020). 2025:6(2):e70057. 10.1002/mco2.70057. 39931738 PMC11809559

[msaf020-B84] Xiao Q-X, Chen J-J, Fang C-L, Su Z-Y, Wang T-H. Neurotrophins-3 plays a vital role in anti-apoptosis associated with NGF and BDNF regulation in neonatal rats with hypoxic-ischemic brain injury. Ibrain. 2020:6(2):12–17. 10.1002/j.2769-2795.2020.tb00047.x.

[msaf020-B85] Yan S-S, Campos de Souza S, Xie Z-D, Bao Y-X. Research progress in clinical trials of stem cell therapy for stroke and neurodegenerative diseases. Ibrain. 2023:9(2):214–230. 10.1002/ibra.12095. 37786546 PMC10529019

[msaf020-B86] Yang AC, Vest RT, Kern F, Lee DP, Agam M, Maat CA, Losada PM, Chen MB, Schaum N, Khoury N, et al A human brain vascular atlas reveals diverse mediators of Alzheimer’s risk. Nature. 2022:603(7903):885–892. 10.1038/s41586-021-04369-3. 35165441 PMC9635042

[msaf020-B87] Yu G, Wang LG, Han Y, He QY. clusterProfiler: an R package for comparing biological themes among gene clusters. Omics. 2012:16(5):284–287. 10.1089/omi.2011.0118. 22455463 PMC3339379

[msaf020-B88] Yu Y, Yu S, Battaglia G, Tian X. Amyloid-ß in Alzheimer’s disease: structure, toxicity, distribution, treatment, and prospects. Ibrain. 2024:10:266–289. 10.1002/ibra.12155. 39346788 PMC11427815

[msaf020-B89] Zhang B, Gaiteri C, Bodea LG, Wang Z, McElwee J, Podtelezhnikov AA, Zhang C, Xie T, Tran L, Dobrin R, et al Integrated systems approach identifies genetic nodes and networks in late-onset Alzheimer’s disease. Cell. 2013:153(3):707–720. 10.1016/j.cell.2013.03.030. 23622250 PMC3677161

[msaf020-B90] Zhang H, Li J, Ren J, Sun S, Ma S, Zhang W, Yu Y, Cai Y, Yan K, Li W, et al Single-nucleus transcriptomic landscape of primate hippocampal aging. Protein Cell. 2021:12(9):695–716. 10.1007/s13238-021-00852-9. 34052996 PMC8403220

[msaf020-B91] Zhao W, Morinaga J, Ukawa S, Endo M, Yamada H, Kawamura T, Wakai K, Tsushita K, Ando M, Suzuki K, et al Plasma angiopoietin-like protein 2 levels and mortality risk among younger-old Japanese people: a population-based case-cohort study. J Gerontol A Biol Sci Med Sci. 2022:77(6):1150–1158. 10.1093/gerona/glac017. 35037044

[msaf020-B92] Zhong S, Ding W, Sun L, Lu Y, Dong H, Fan X, Liu Z, Chen R, Zhang S, Ma Q, et al Decoding the development of the human hippocampus. Nature. 2020:577(7791):531–536. 10.1038/s41586-019-1917-5. 31942070

[msaf020-B93] Zhong S, Zhang S, Fan X, Wu Q, Yan L, Dong J, Zhang H, Li L, Sun L, Pan N, et al A single-cell RNA-seq survey of the developmental landscape of the human prefrontal cortex. Nature. 2018:555(7697):524–528. 10.1038/nature25980. 29539641

[msaf020-B94] Zhou Y, Bond AM, Shade JE, Zhu Y, Davis CO, Wang X, Su Y, Yoon KJ, Phan AT, Chen WJ, et al Autocrine Mfge8 signaling prevents developmental exhaustion of the adult neural stem cell pool. Cell Stem Cell. 2018:23(3):444–452.e4. 10.1016/j.stem.2018.08.005. 30174295 PMC6128767

[msaf020-B95] Zhu J, Chen F, Luo L, Wu W, Dai J, Zhong J, Lin X, Chai C, Ding P, Liang L, et al Single-cell atlas of domestic pig cerebral cortex and hypothalamus. Sci Bull (Beijing). 2021:66(14):1448–1461. 10.1016/j.scib.2021.04.002. 36654371

[msaf020-B96] Ziegler AN, Levison SW, Wood TL. Insulin and IGF receptor signalling in neural-stem-cell homeostasis. Nat Rev Endocrinol. 2015:11(3):161–170. 10.1038/nrendo.2014.208. 25445849 PMC5513669

