# Supplementary material for: Cross-Species Insights from Single-Nucleus Sequencing Highlight Aging-Related Hippocampal Features in Tree Shrew
Source: Mol Biol Evol. 2025 Feb 28;42(2):msaf020. doi: 10.1093/molbev/msaf020 (PMC11879083; doi:10.1093/molbev/msaf020)
Supplement: msaf020_Supplementary_Data [file msaf020_supplementary_data.zip › Supplementary materials.pdf]

Supplementary Materials for

Cross-species insights from single-nucleus sequencing highlight aging-related hippocampal features in tree shrew

Xiong et al.

This PDF file includes:

Fig. S1 to S16
Captions of Tables S1 to S15

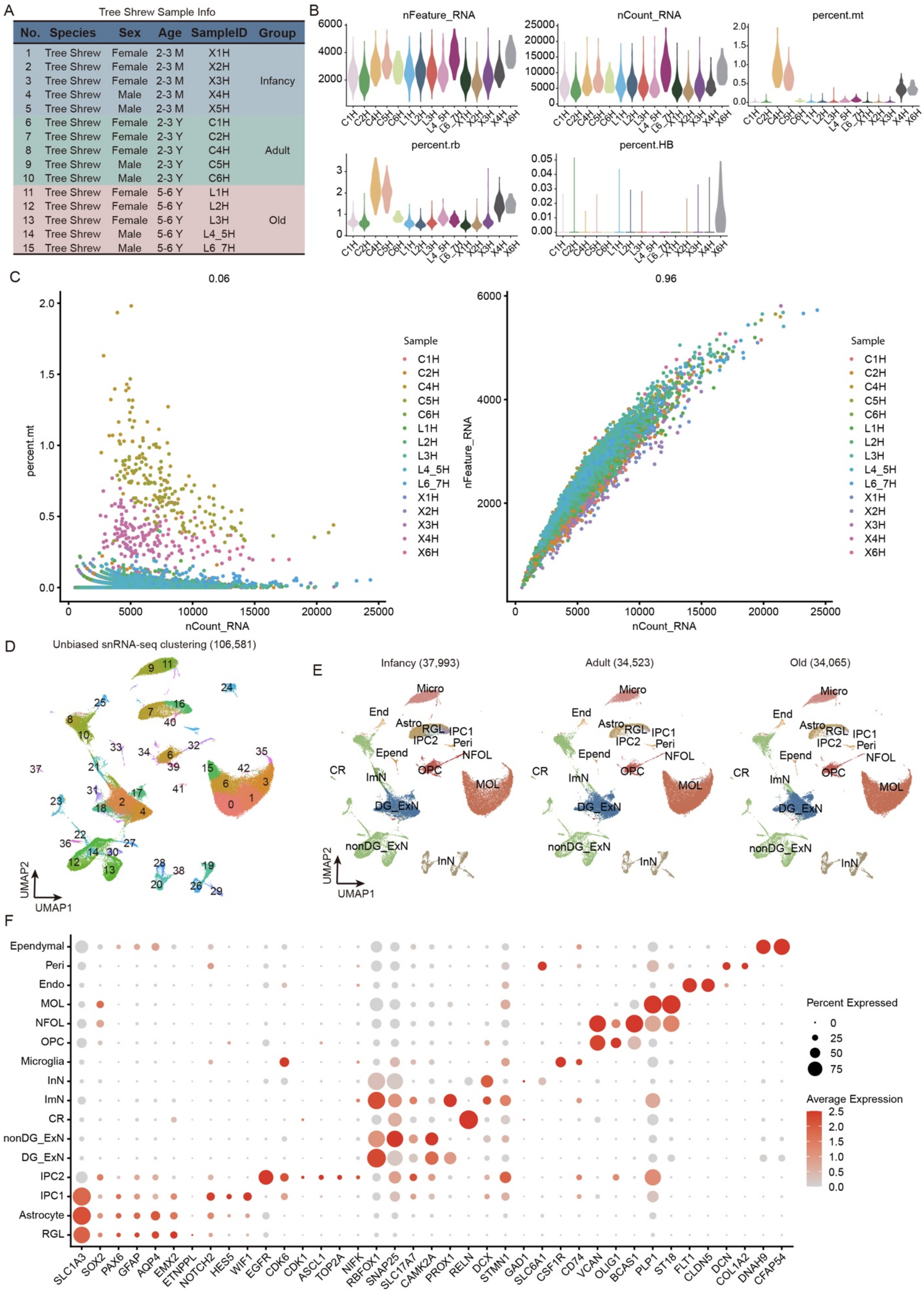
A
Tree Shrew Sample Info
No. Species Sex Age SampleID Group
1 Tree Shrew Female 2-3 M X1H
2 Tree Shrew Female 2-3 M X2H
3 Tree Shrew Female 2-3 M X3H Infancy
4 Tree Shrew Male 2-3 M X4H
5 Tree Shrew Male 2-3 M X5H
6 Tree Shrew Female 2-3 Y C1H
7 Tree Shrew Female 2-3 Y C2H
8 Tree Shrew Female 2-3 Y C4H Adult
9 Tree Shrew Male 2-3 Y C5H
10 Tree Shrew Male 2-3 Y C6H
11 Tree Shrew Female 5-6 Y L1H
12 Tree Shrew Female 5-6 Y L2H
13 Tree Shrew Female 5-6 Y L3H Old
14 Tree Shrew Male 5-6 Y L4_5H
15 Tree Shrew Male 5-6 Y L6_7H
B
nFeature_RNA
nCount_RNA
percent.mt
percent.rb
percent.HB
C
0.06
0.96
percent.mt
nFeature_RNA
nCount_RNA
Sample
C1H C2H C4H C5H C6H L1H L2H L3H L4_5H L6_7H X1H X2H X3H X4H X6H
D
Unbiased snRNA-seq clustering (106,581)
UMAP1
UMAP2
E
Infancy (37,993)
Adult (34,523)
Old (34,065)
Micro End Astro RGL IPC1 IPC2 Peri Epend NFOL OPC CR ImN MOL DG_ExN nonDG_ExN InN
F
Ependymal Peri Endo MOL NFOL OPC Microglia InN ImN CR nonDG_ExN DG_ExN IPC2 IPC1 Astrocyte RGL
SLC1A3 SOX2 PAX6 GFAP AQP4 EMX2 ETNPPL NOTCH2 HES5 WIF1 EGFR CDK6 CDK1 ASCL1 TOP2A NFIX RBFOX1 SNAP25 SLC17A7 CAMK2A PROX1 RELN DCX STMN1 GAD1 SLC6A1 CSF1R CD74 VCAN OLIG1 BCAS1 PLP1 ST18 FLT1 CLDN5 DCN COL1A2 DNAH9 CFAP54
Percent Expressed
0
25
50
75
Average Expression
2.5
2.0
1.5
1.0
0.5
0.0

Supplementary Fig. 1 Transcriptomic cell type taxonomy of TS hippocampus. (A) Detailed information of individual TS included in this study. (B) Violin plots show 15 snRNA-seq data quality of nFeature_RNA, nCount_RNA, percent.mt, percent.rb, and percent.HB. (C) Left: Scatter plot showing the relationship between the total UMI counts (nCount_RNA) and the proportion of mitochondrial genes (percent.mt) for each cell across different samples. Each dot represents a single cell, colored by sample. A low proportion of mitochondrial genes (< 5–10%) indicates good quality cells, while higher values may suggest stressed or dying cells. Right: Scatter plot showing the correlation between the total UMI counts (nCount_RNA) and the number of detected genes (nFeature_RNA) for each cell across different samples. Each dot represents a single cell, colored by sample. The high correlation (R = 0.96) suggests consistent detection of features across cells, which indicates data quality. (D) UMAP plot showing distribution of cell cluster in the TS hippocampus. (E) UMAP plot showing distribution of cell types in the TS hippocampus. Labels in UMAP: RGL, radial glia-like cells; IPC, intermediate progenitor cells; Astro, Astrocytes; CR, Cajal–Retzius cells; DG, dentate gyrus; ExN, excitatory neurons; InN, inhibitory neurons; Astro, astrocytes; Micro, microglia; MOL, mature oligodendrocytes; OPC, oligodendrocyte progenitor cells; Endo, endothelial cells; Peri, pericytes. (F) Bubble dot plots of the top cluster specific marker genes. The size of the dot indicates expression percentage and the darkness of the color indicates average expression.

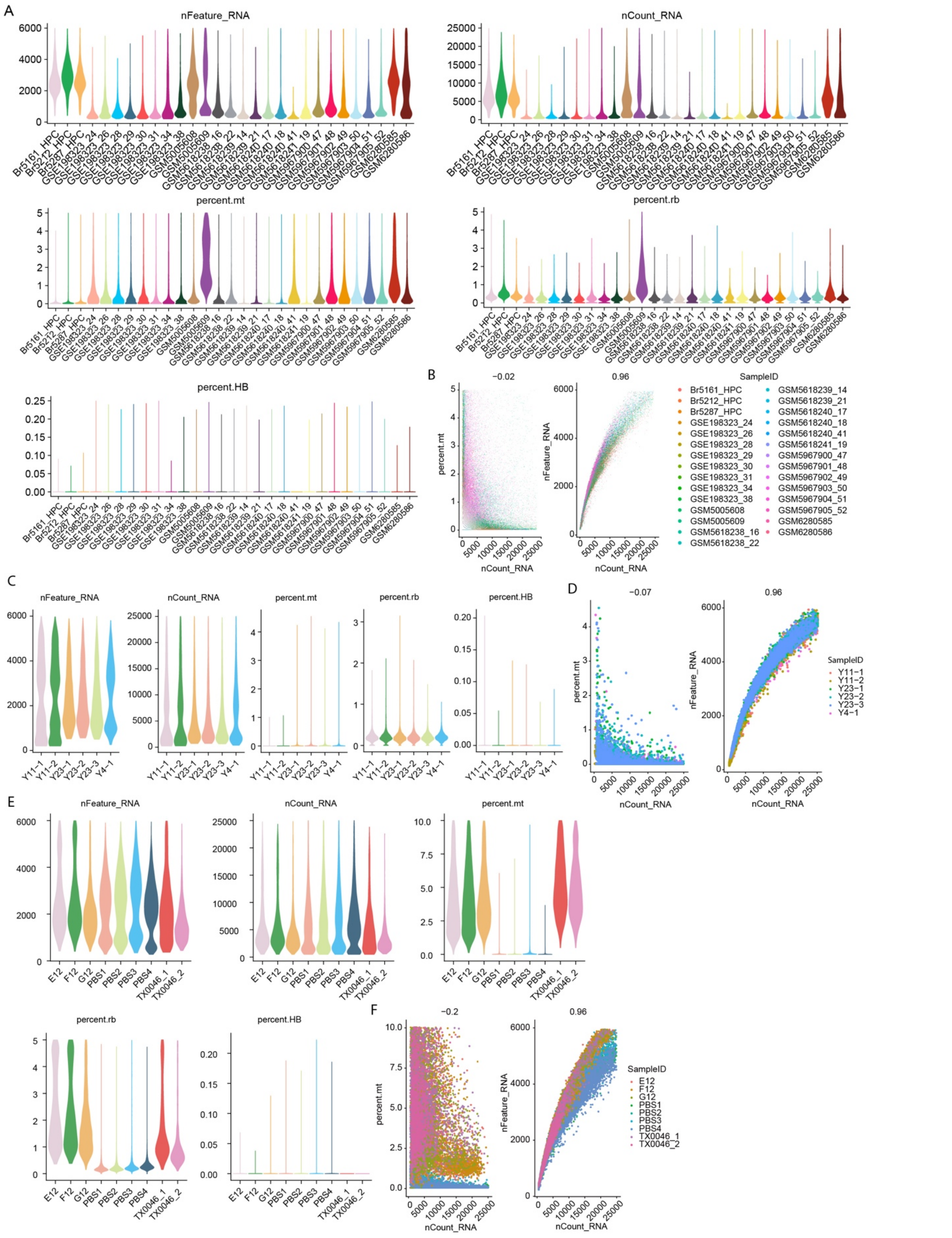

Supplementary Fig. 2 Quality control (QC) metrics of snRNA-seq data across different samples from human, macaque and mouse. (A) Violin plots showing QC metrics across human

snRNA-seq samples. Each violin represents one sample, showing the distribution of nFeature_RNA, nCount_RNA, percent.mt, percent.rb and percent. HB to assess data quality. nFeature_RNA: Number of detected genes per cell. nCount_RNA: Total number of UMIs per cell. percent.mt: Percentage of mitochondrial gene counts per cell. percent.rb: Percentage of ribosomal gene counts per cell. percent. HB: Percentage of hemoglobin gene counts per cell. (B) Scatter plots of mitochondrial gene percentage (percent.mt) versus total UMI counts (nCount_RNA) (left), and the correlation between total UMI counts (nCount_RNA) and the number of detected genes (nFeature_RNA) (right). The R values (-0.02 and 0.96) indicate weak correlation between mitochondrial gene percentage and UMI counts, and strong correlation between UMI counts and gene detection, respectively. (C) Violin plots of QC metrics (nFeature_RNA, nCount_RNA, percent.mt, percent.rb, and percent.HB) grouped by macaque sample. (D) Scatter plots for sample groups Y1–Y4 showing percent.mt versus nCount_RNA (left) and nFeature_RNA versus nCount_RNA (right), with corresponding R values of -0.07 and 0.96. (E) Violin plots of QC metrics for mouse snRNA-seq sample. The plots display distributions of nFeature_RNA, nCount_RNA, percent.mt, percent.rb, and percent.HB for each group. (F) Scatter plots for mouse sample showing percent.mt versus nCount_RNA (left) and nFeature_RNA versus nCount_RNA (right), with corresponding R values of -0.2 and 0.96.

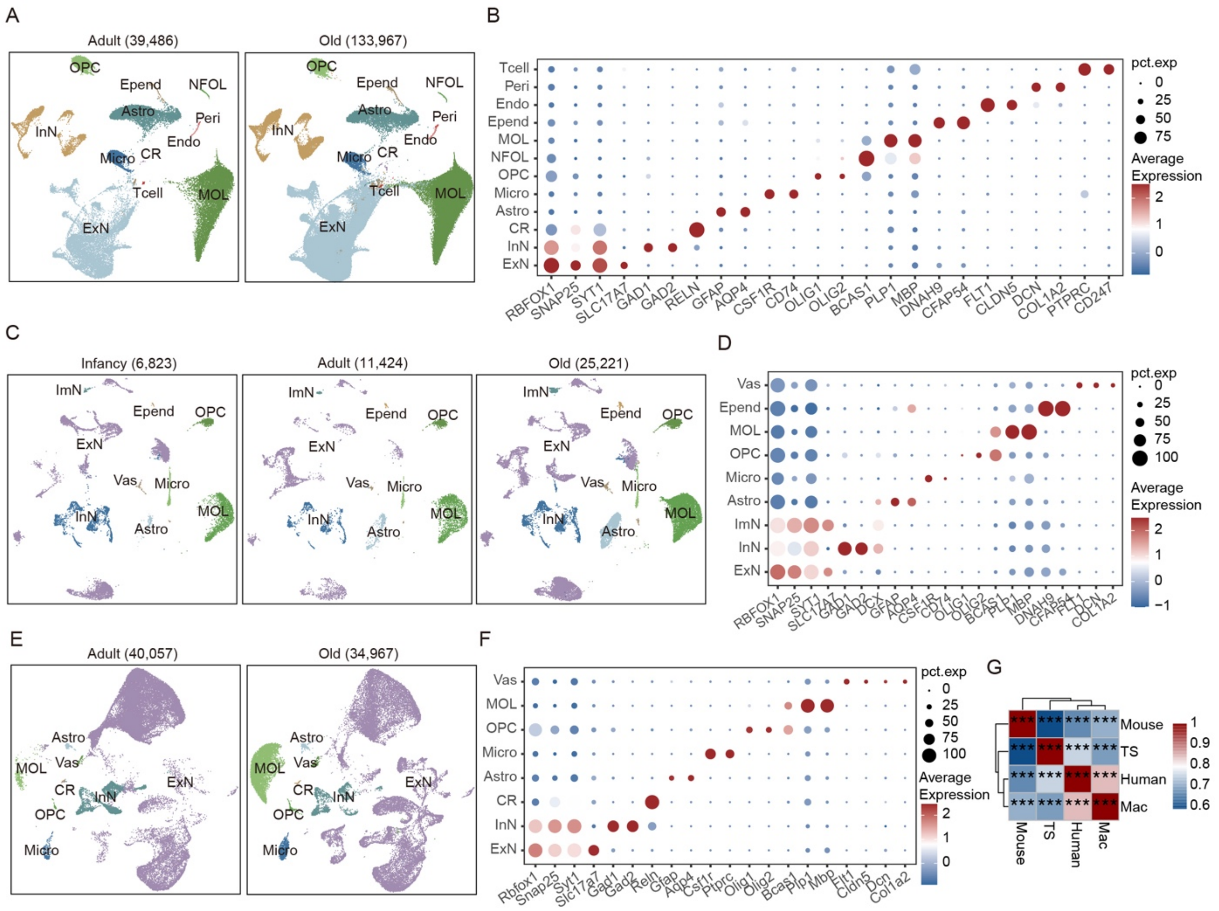

Supplementary Fig. 3 Cross-species comparison of cell types across human, monkey, and mouse hippocampal snRNA-seq data. (A) UMAP visualization of hippocampal cell clusters in adult (left, n = 39,486) and old (right, n = 133,967) human samples. Major cell types are labeled, including excitatory neurons (ExN), inhibitory neurons (InN), oligodendrocyte precursor cells (OPC), astrocytes (Astro), microglia (Micro), oligodendrocytes (MOL), vascular cells (Vas), endothelial cells (Endo), and other clusters. (B) Dot plot showing marker gene expression across identified cell types in human hippocampus. The size of each dot represents the percentage of cells expressing the gene (pct.exp), and the color indicates the average expression level. (C) UMAP visualization of hippocampal cell clusters in infancy (left, n = 6,823), adult (middle, n = 11,424), and old (right, n = 25,221) macaque samples. (D) Dot plot showing marker gene expression across monkey hippocampal cell types, with dot size indicating the percentage of cells expressing the gene and color representing the average expression level. (E) UMAP visualization of hippocampal cell clusters in adult (left, n = 40,057) and old (right, n = 34,967) samples. (F) Dot plot showing marker gene expression across mouse hippocampal cell types, with dot size representing the proportion of expressing cells and color indicating average expression levels. (G) Heatmap showing pairwise correlations of cell-type-specific gene expression profiles across species (mouse, TS, human, and macaque). Higher correlations (red) indicate conserved expression patterns, while lower correlations (blue) highlight species-specific differences. Statistical significance is indicated with asterisks (spearman; *$P < 0.05$, **$P < 0.01$, ***$P < 0.001$, significance test for Spearman's rank correlation coefficient using t-distribution approximation).

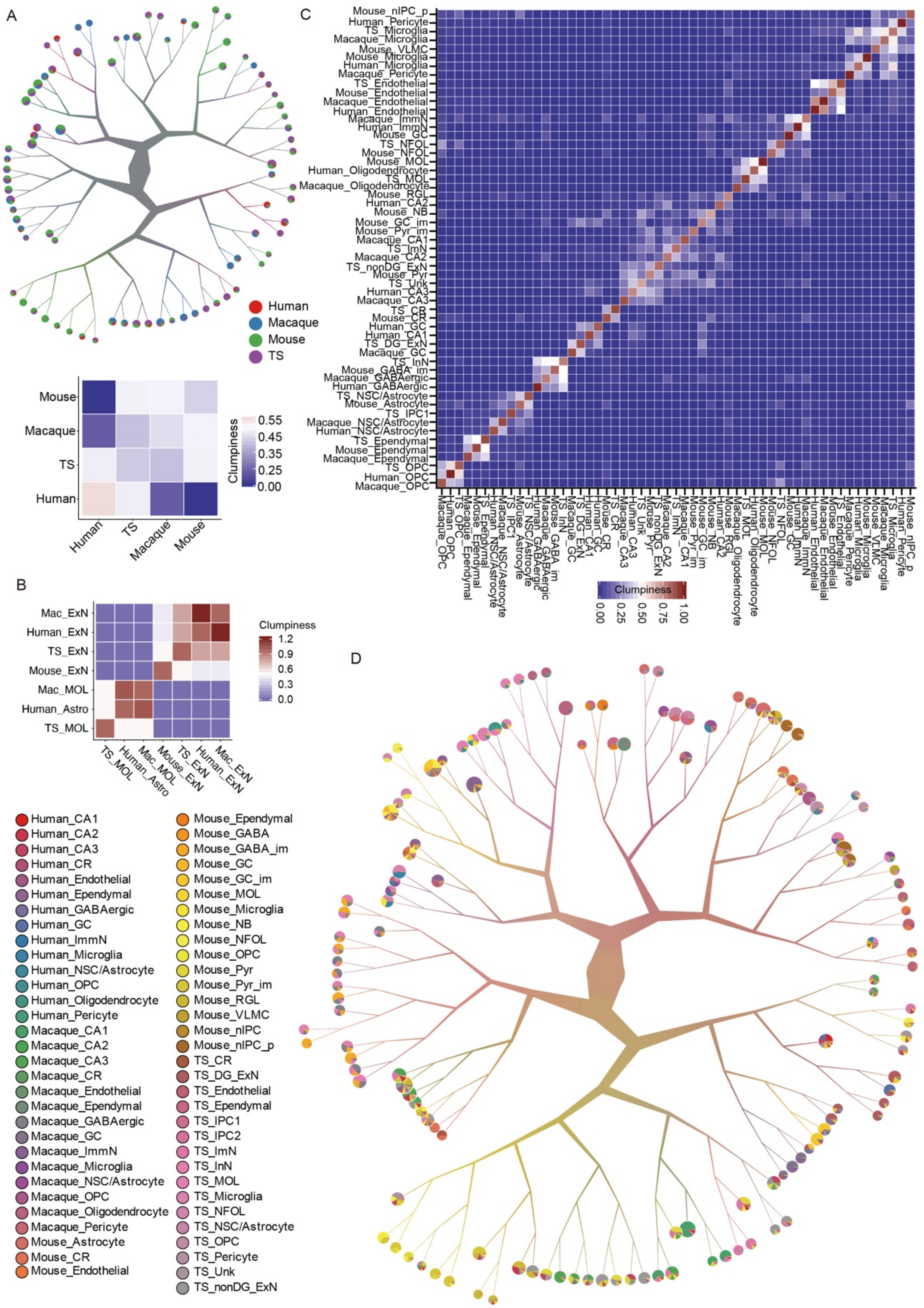

Supplementary Fig. 4 Cross-species single-cell transcriptomic comparison of hippocampal cell types using TooManyCells analysis. (A) Phylogenetic tree of cell types across four species (Human, Macaque, Mouse, and TS). Each node represents a distinct cell type, colored by species:

red for human, green for macaque, blue for mouse, and purple for TS. The overall clustering reflects evolutionary and functional relationships between the identified hippocampal cell types. The heatmap (bottom) shows the degree of clumpiness for each species, where higher values indicate greater cell clustering or co-localization within specific branches. (B) Heatmap highlighting the clumpiness scores for specific cell types under more stringent filtering criteria. Rows and columns correspond to selected major cell types across species, including ExN, MOL, Astro. Higher clumpiness values (red) indicate stronger species-specific clustering. (C) Heatmap showing pairwise clumpiness scores between all cell types across species. Cell types along the x-axis and y-axis are grouped by species (Human, Macaque, Mouse, and TS). Higher clumpiness scores (red) indicate strong co-localization or conserved clustering patterns, whereas lower scores (blue) suggest weaker relationships. (D) Detailed phylogenetic tree of hippocampal cell types, showing finer branching and clustering relationships across species. Nodes are colored by species as in panel A, with specific cell types labeled in the legend. The tree structure highlights conserved and divergent relationships between species, reflecting evolutionary and functional distinctions in hippocampal cell type composition.

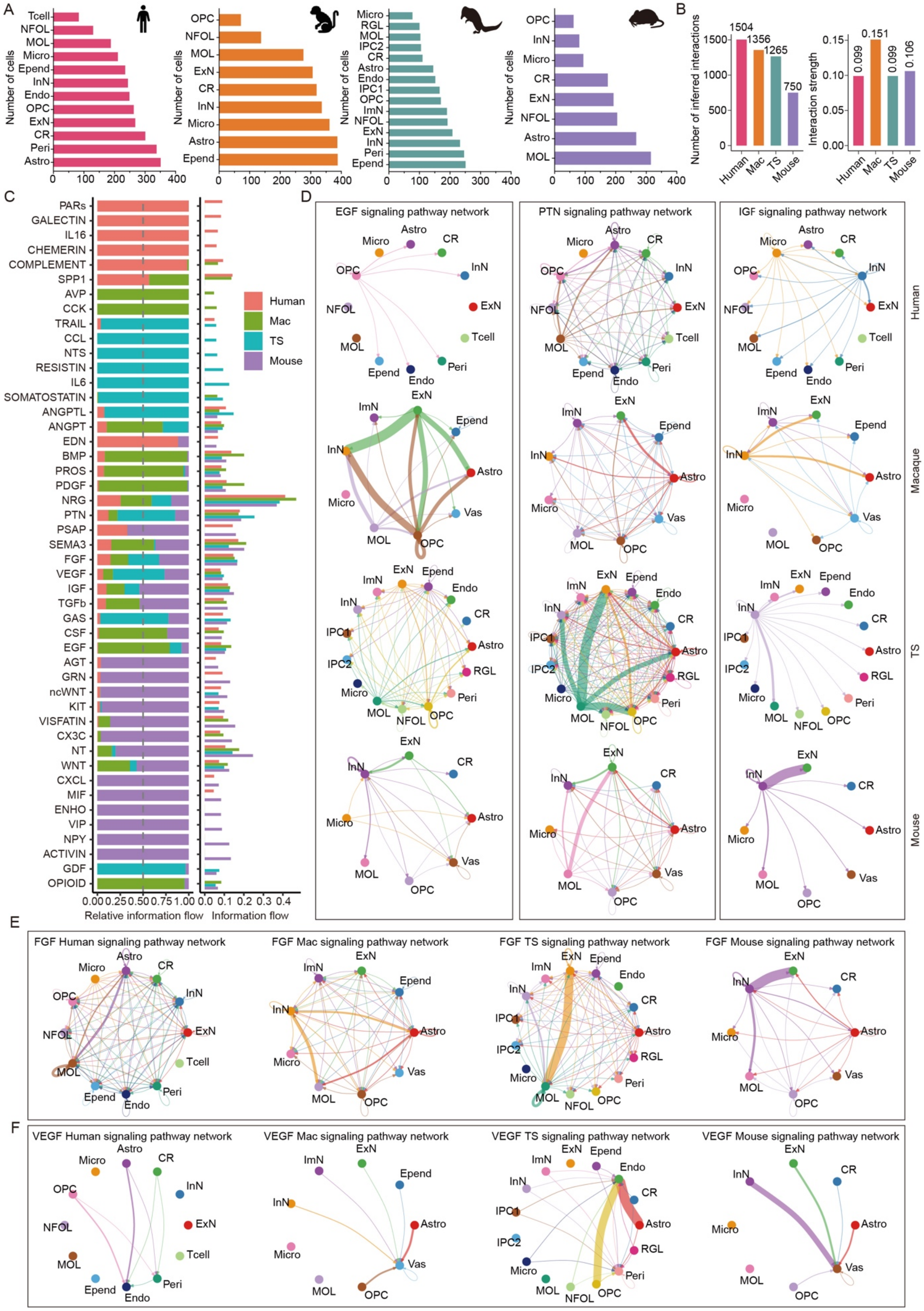
A
Number of cells
Tcell
NFOL
MOL
Micro
Epend
InN
Endo
OPC
ExN
CR
Peri
Astro
RGL
IPC2
IPC1
ImN
0 100 200 300 400
B
Number of inferred interactions
1504
1356
1265
750
Human
Mac
TS
Mouse
Interaction strength
0.099
0.151
0.099
0.106
C
PARs
GALECTIN
IL16
CHEMERIN
COMPLEMENT
SPP1
AVP
CCK
TRAIL
CCL
NTS
RESISTIN
IL6
SOMATOSTATIN
ANGPTL
ANGPT
EDN
BMP
PROS
PDGF
NRG
PTN
PSAP
SEMA3
FGF
VEGF
IGF
TGFb
GAS
CSF
EGF
AGT
GRN
ncWNT
KIT
VISFATIN
CX3C
NT
WNT
CXCL
MIF
ENHO
VIP
NPY
ACTIVIN
GDF
OPIOID
Human
Mac
TS
Mouse
0.00 0.25 0.50 0.75 1.00
Relative information flow
0.0 0.1 0.2 0.3 0.4
Information flow
D
EGF signaling pathway network
PTN signaling pathway network
IGF signaling pathway network
Human
Macaque
TS
Mouse
Vas
E
FGF Human signaling pathway network
FGF Mac signaling pathway network
FGF TS signaling pathway network
FGF Mouse signaling pathway network
F
VEGF Human signaling pathway network
VEGF Mac signaling pathway network
VEGF TS signaling pathway network
VEGF Mouse signaling pathway network

Supplementary Fig.5 Comparison of cell communication and signaling pathway across species. (A) Bar plots showing the number of species-related cell-cell interactions between the indicated cell type with other cell types in hippocampus. (B) Cross-species comparison of inferred cell-cell interactions and interaction strengths. Left: Bar plot showing the number of inferred cell-cell interactions across species. Human (1504) exhibits the highest number of interactions, followed by macaque (1356), TS (1265), and mouse (750), indicating species-specific differences in cell-cell communication complexity. Right: Bar plot showing the average interaction strength for each species. Macaque shows the highest interaction strength (0.151), while human (0.099), tree shrew (0.099), and mouse (0.106) display lower interaction strengths. (C) Bar plots showing the strength of species-related cell-cell interactions in hippocampus. (D) Comparison of cell–cell communication of *EGF*, *PTN and IGF* signaling pathway in human, macaque, TS and mouse. (E) Comparison of cell–cell communication of *FGF* signaling pathway in human, macaque, TS and mouse. (F) Comparison of cell–cell communication of *VEGF* signaling pathway in human, macaque, TS and mouse.

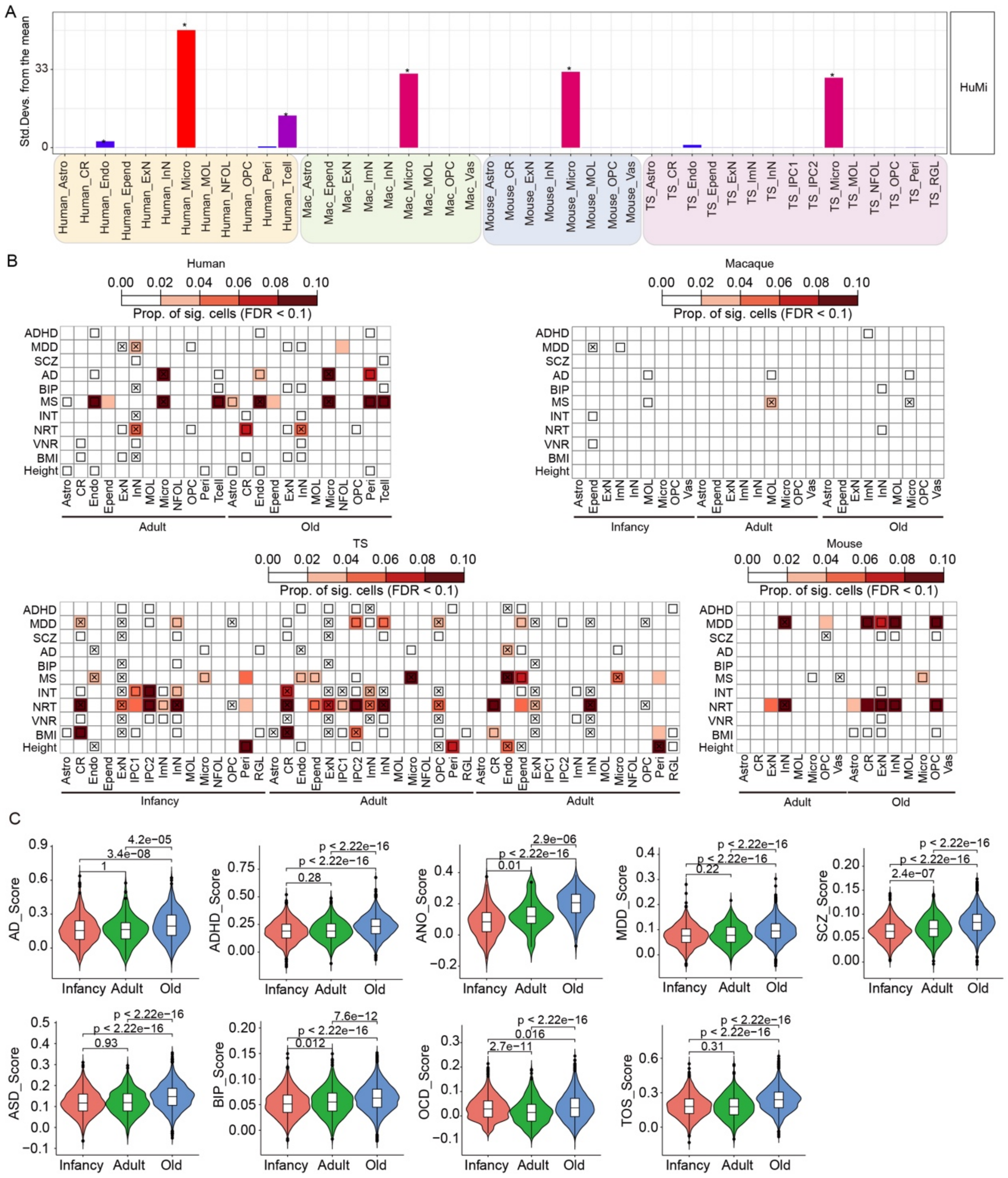

Supplementary Fig.6 Cross-species and age-specific cell-type associations with neuropsychiatric and neurological disorders using EWCE and scDRS analysis. (A) Bar plot showing the standard deviation (SD) from the median for significant gene enrichment across specific cell types in different species (Human, Macaque, Mouse, and TS). (B) Heatmaps display the proportion of significant cells (FDR < 0.1) associated with various neuropsychiatric and neurological disorders across species and age groups. Disorders analyzed include AD (Alzheimer's disease), SCZ (schizophrenia), MDD (major depressive disorder), ASD (autism spectrum disorder), BIP (bipolar disorder), ADHD. Squares denote significant tissues-disease associations across all pairs of cell and diseases/ traits. Cross symbols denote significant heterogeneity in association with disease across individual cells within a given cell type. Red shading indicates higher proportions of significant cells, with darker shades reflecting stronger associations. (C)

Violin plots comparing disorder-associated scores (e.g., AD, ADHD, ANO, MDD, SCZ, ASD, BIP, OCD, and TOS) across infancy, adult, and old age groups. The statistical significance of differences between age groups is indicated with *P*-values (two-sided Wilcoxon rank-sum test). Disorders such as MDD, SCZ, and ASD show significant increases in relevance scores with aging, particularly in the adult and old groups.

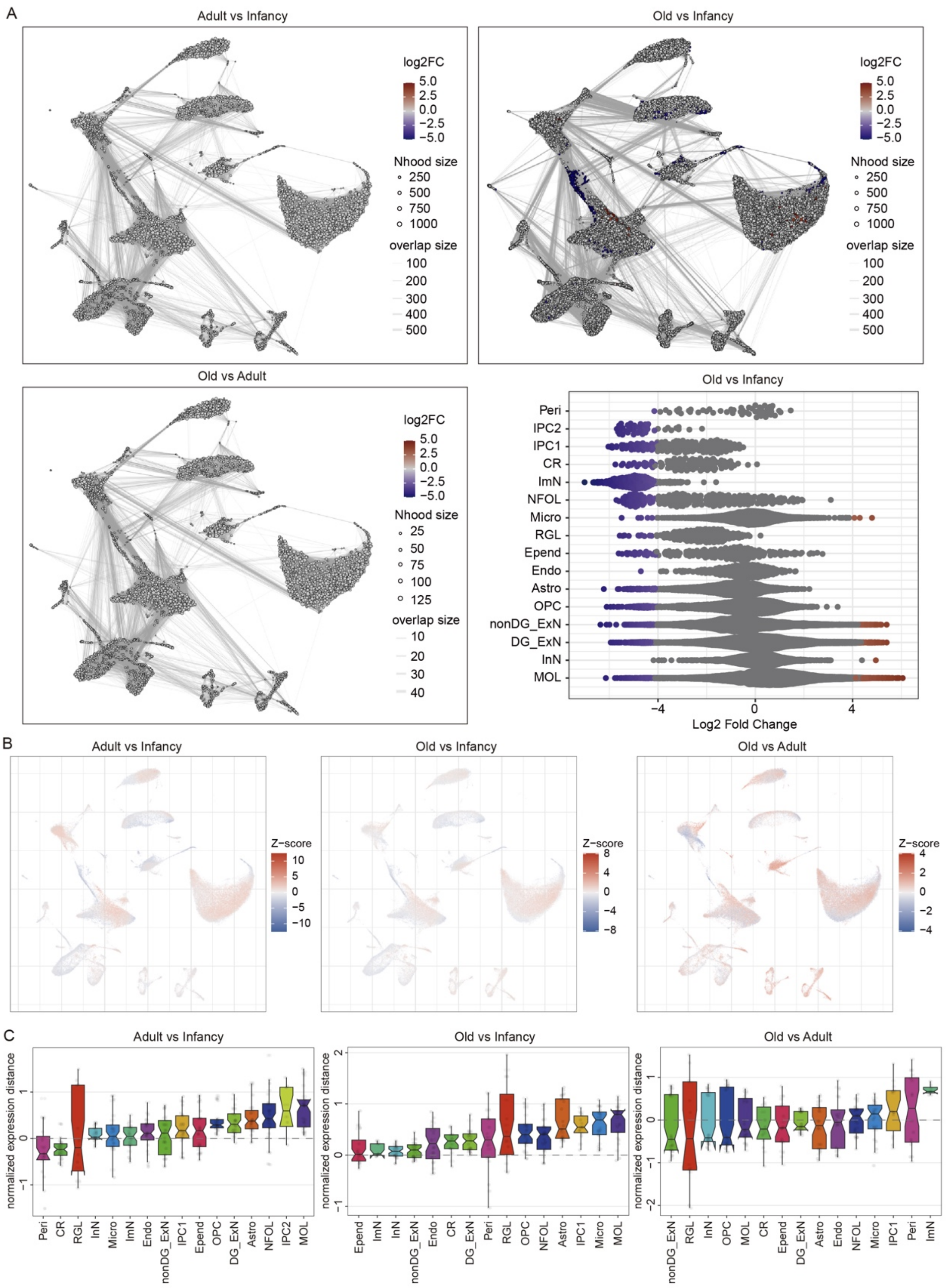

Supplementary Fig.7 Differential abundance and expression analysis of hippocampal cell types across developmental stages using Milo and Cacoa. (A) Neighborhood graph from Milo differential abundance analysis for the cell from different group. Nodes represent cellular neighborhoods and edges show neighborhood connections from the KNN graph. Node size reflects the number of cells in each neighborhood, while edge thickness indicates the degree of overlap between neighborhoods. Differential abundance neighborhoods are colored by the log-transformed

fold change across ages. Colors represent the log2 fold change (log2FC) in cell abundance, with positive changes in red and negative changes in blue. Top left: Adult vs Infancy; Top right: Old vs Infancy; Bottom left: Old vs Adult. (B) Z-score maps the cell from different group. Z-scores represent normalized changes in cell abundance across developmental stages. Red indicates increased, while blue indicates decreased. Specific regions of the UMAP show strong developmental differences. (C) Normalized expression distance for specific cell types. Boxplots showing the normalized expression distance for major cell types across different comparisons.

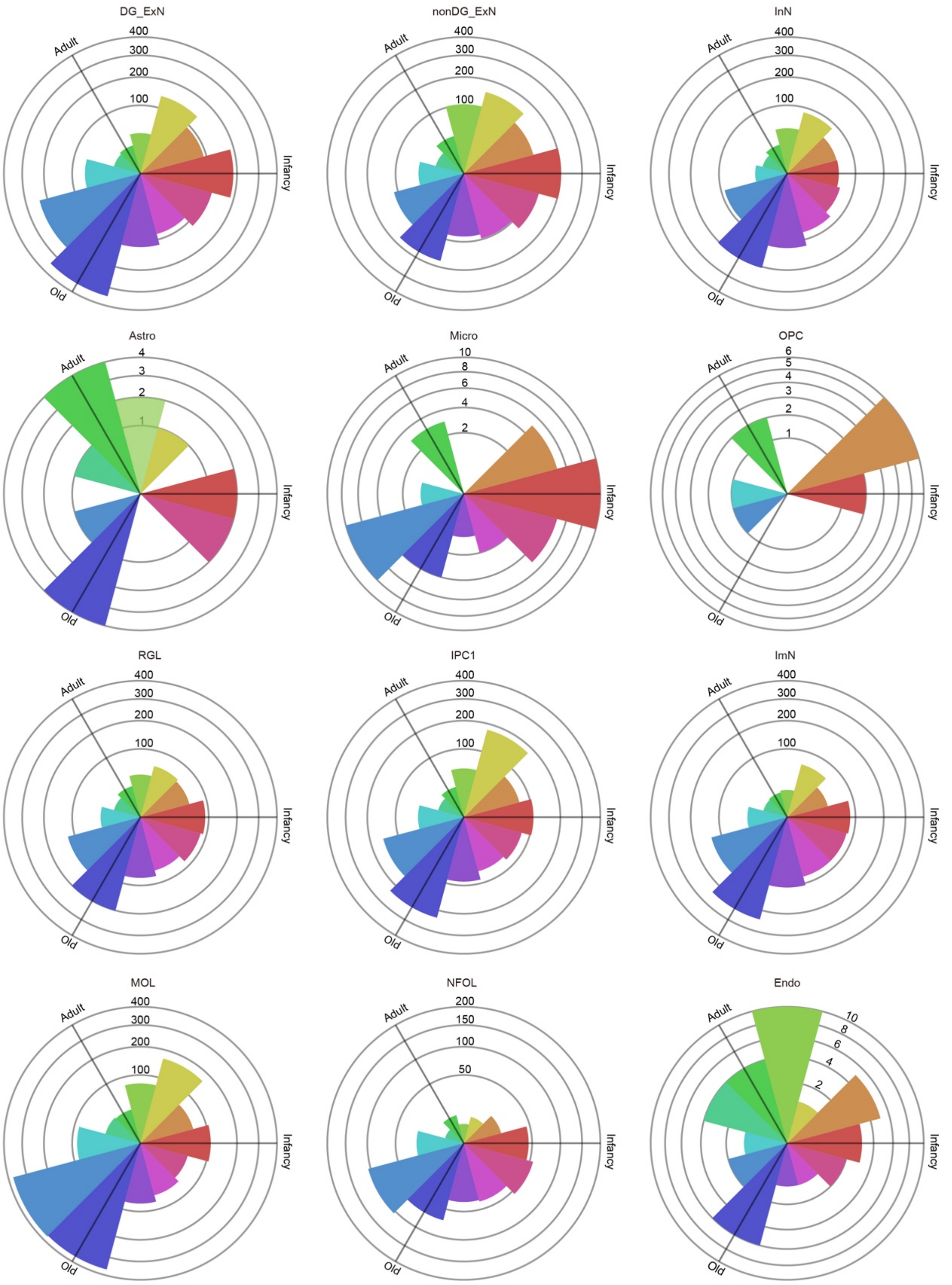

Supplementary Fig.8 Triwise analysis of DEGs (MAST, FDR-adjusted *P*-value < 0.05, $|\log_2 FC| > 0.25$) across three age groups (Infancy, Adult, and Old). Each radar plot represents the distribution of DEGs for a specific cell type (labeled on each plot) across three age groups: Infancy, Adult, and Old. The direction and size of each bar indicate the magnitude and number of DEGs, highlighting changes in gene expression between developmental and aging stages.

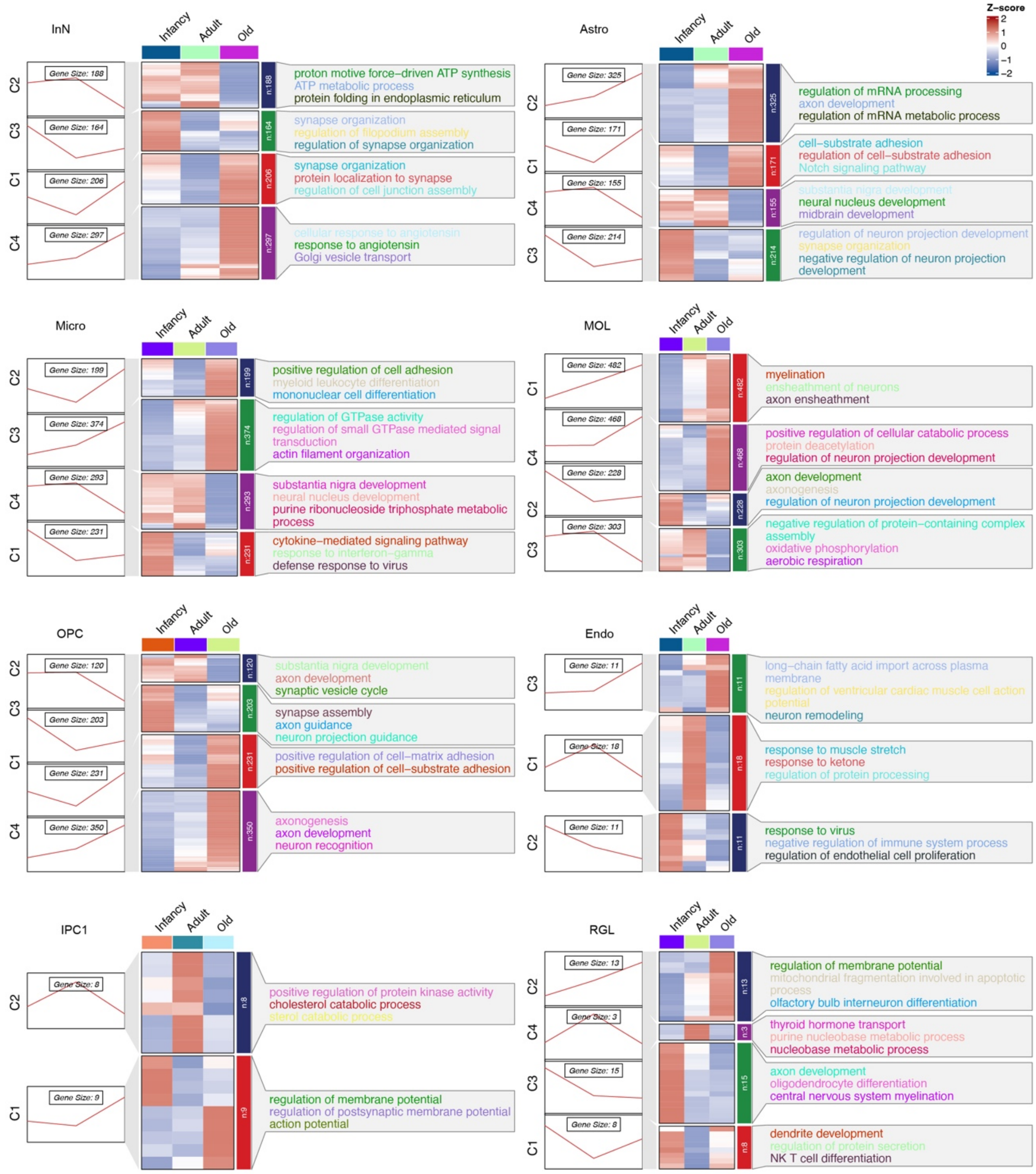

Supplementary Fig.9. Enrichment analysis of age-dependent gene clusters in various cell types across Infancy, Adult, and Old age groups. This figure presents age-associated transcriptional changes across diverse hippocampal cell types. Each panel corresponds to a specific cell type, with heatmaps and gene ontology (GO) enrichment terms. Gene clusters (C1, C2, C3, C4) represent distinct expression patterns and functional enrichment. Heatmaps display Z-scores of gene expression for age-dependent clusters across infancy, adult, and old stages. Red indicates upregulation, blue indicates downregulation. GO Enrichment Terms: Biological processes (BPs) enriched within each gene cluster are displayed next to the heatmaps.

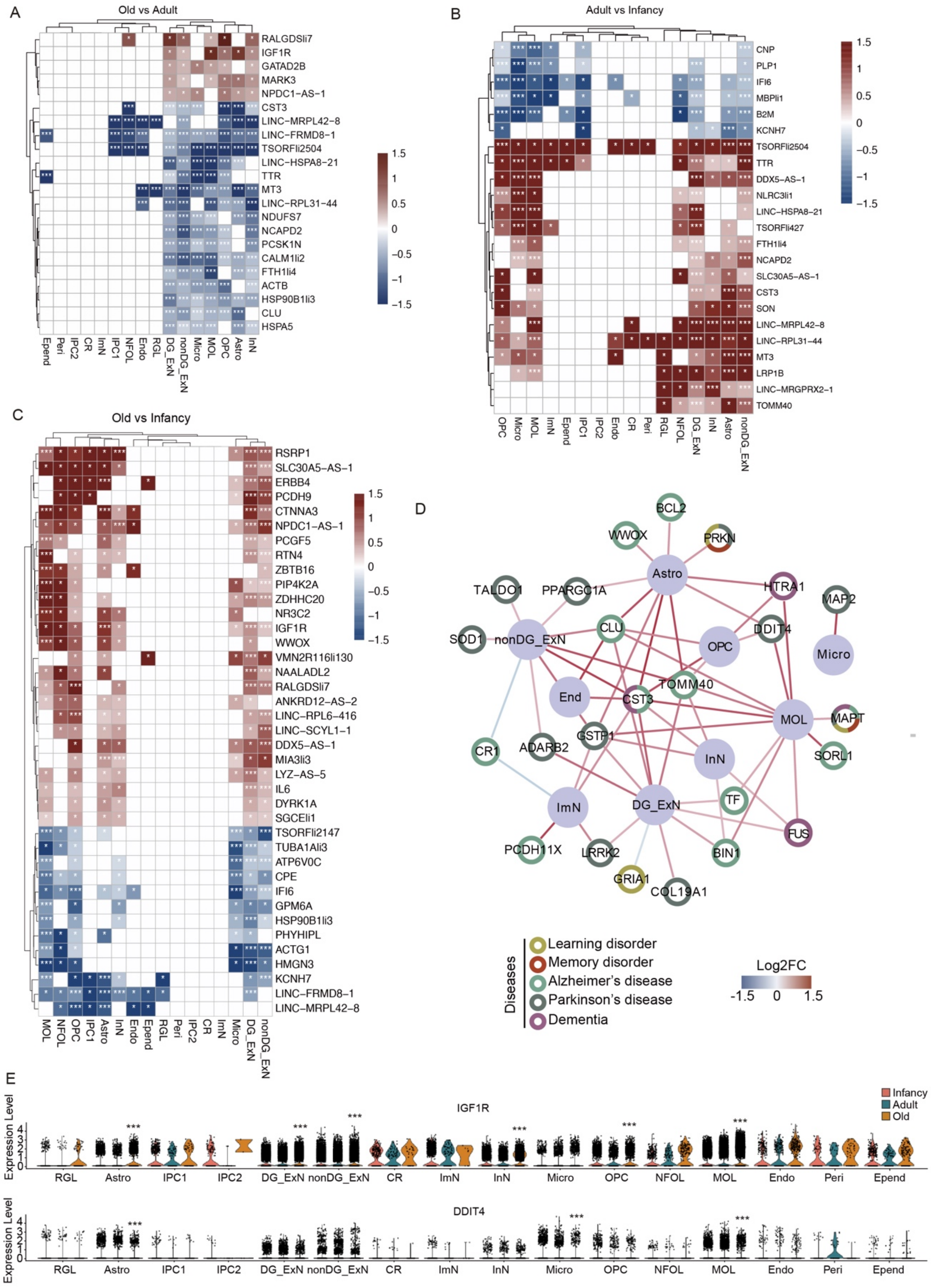

Supplementary Fig.10 Age-associated gene expression analysis of TS hippocampus across different cell types. (A-C) Heatmap showing genes differentially expressed (Adult vs Infancy, Aging vs Infancy, and Old vs Adult) in at least six cell types in the TS hippocampus (adjusted *P*.value< 0.05, |logFC| >0.25). Only genes with same direction of differential expression among

different cell types are included. (D) Network plot showing DEGs (adjusted *P*.value< 0.05, |logFC| > 0.25) associated with aging-related diseases in different cell types in theTS hippocampus. (E) Violin plots showing the expression levels of IGF1R and DDIT4 in TS hippocampus from infancy, adult and old groups (****P*<0.001).

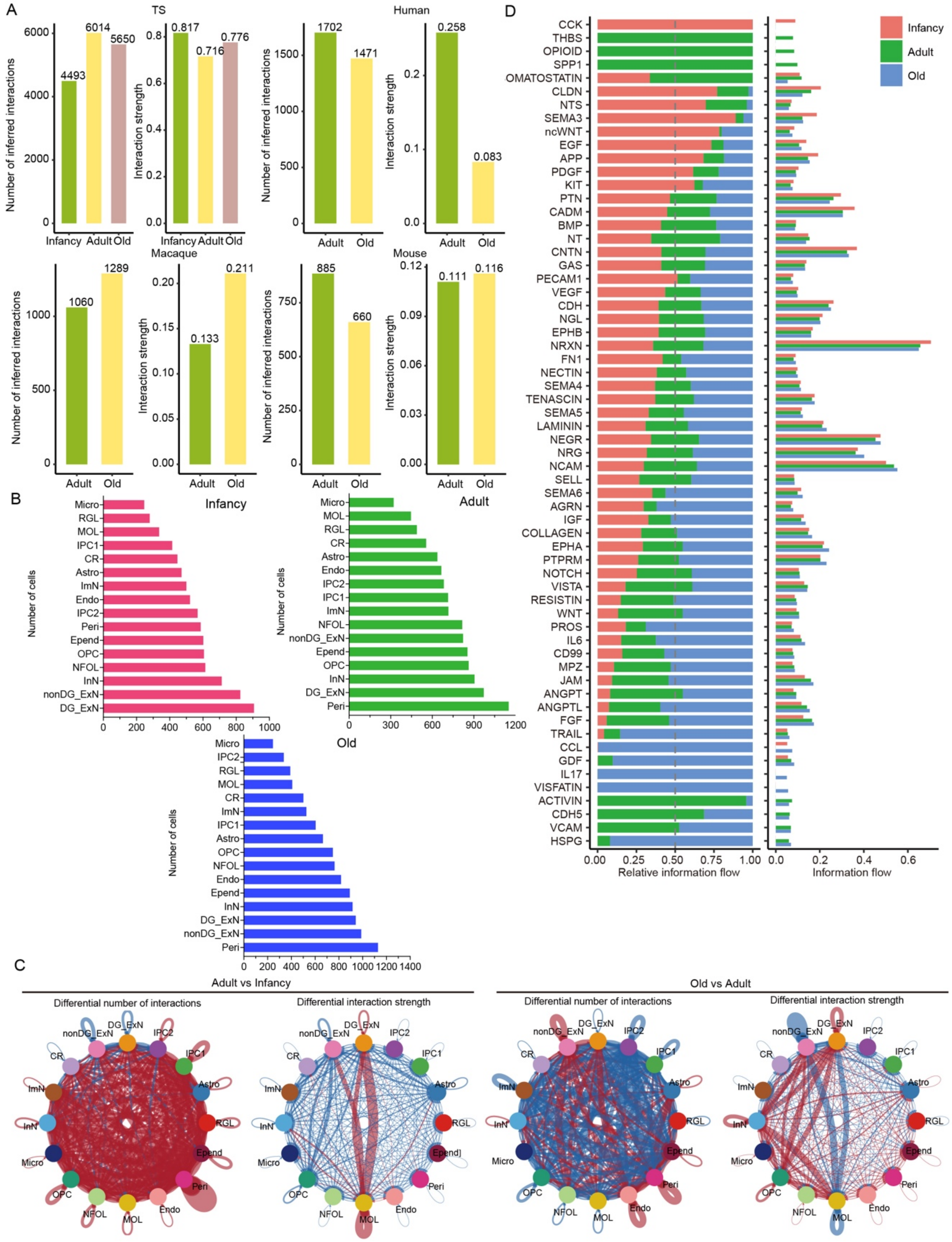

Supplementary Fig.11 Cellular and molecular aging characteristics of the the TS hippocampus. (A) Comparison among groups of inferred cell-cell interactions and interaction strengths from different species. Left: Bar plot showing the number of inferred cell-cell interactions across species. Right: Bar plot showing the average interaction strength for each species. (B) Bar plots showing the number of age-related cell-cell interactions between the

indicated cell type with other cell types in TS hippocampus. (C) Network plot showing the numbers of changed cell-cell interactions between indicated cell types in TS hippocampus. Edge color from blue to red indicates the number from low to high. (D) Bar plots of the ranking of signaling pathway by overall information flow differences in the interaction networks among infancy, adult and old TS hippocampus.

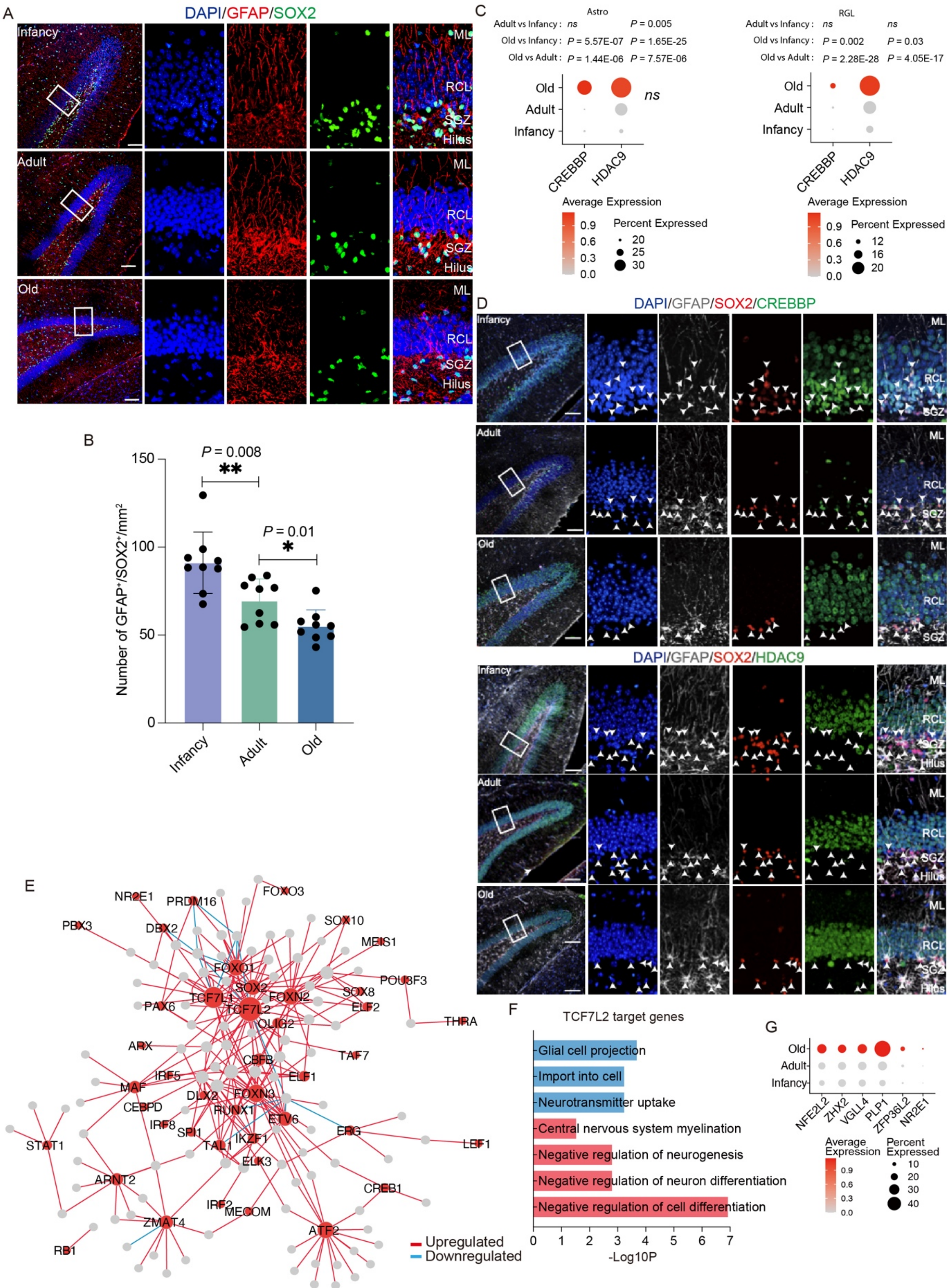

Supplementary Fig.12 Aging-related cellular and molecular alterations of the TS NSCs. (A) Representative microscopic fields of *GFAP* (red) /*SOX2* (green) double-positive cells in the DG

of the hippocampus from infancy, adult and aged TS. Blue, DAPI. Scale bar: low magnification, 100 μm; high magnification, 10 μm. *n* = 9 per group, one-way ANOVA, *$P < 0.05$, **$P < 0.01$. White rectangular boxes represent the focused areas. (B) Quantification of *GFAP/SOX2* double-positive cells in the DG of the hippocampus from infancy, adult and old TS. (C) Dot plots showing expression levels of *CREBBP* and *HDAC9* in NSC (RGL) and astrocyte in the TS hippocampus from infancy, adult, and old groups. (D) Representative microscopic fields of *GFAP* (white) / *SOX2* (red) / *CREBBP* (green) triple-positive cells and *GFAP* (white) / *SOX2* (red) / *HDAC9* (green) triple-positive cells in the DG of the hippocampus from infancy, adult and old TS. Blue, DAPI. Scale bar: low magnification, 100 μm; high magnification, 10 μm. *n* = 4 per group. White rectangular boxes represent the focused areas and white arrowhead indicates the cells of interest. (E) Network plot showing transcriptional regulators of NSC/astrocyte aging-related DEG. (F) Bar plot showing GO terms of target genes of *TCF7L2*. Orange, upregulation; blue, downregulation. (G) Dot plot showing the expression changes of aging-related DEGs associated with negative regulation of neurogenesis in NSC/astrocyte of the TS hippocampus from infancy, adult and old groups.

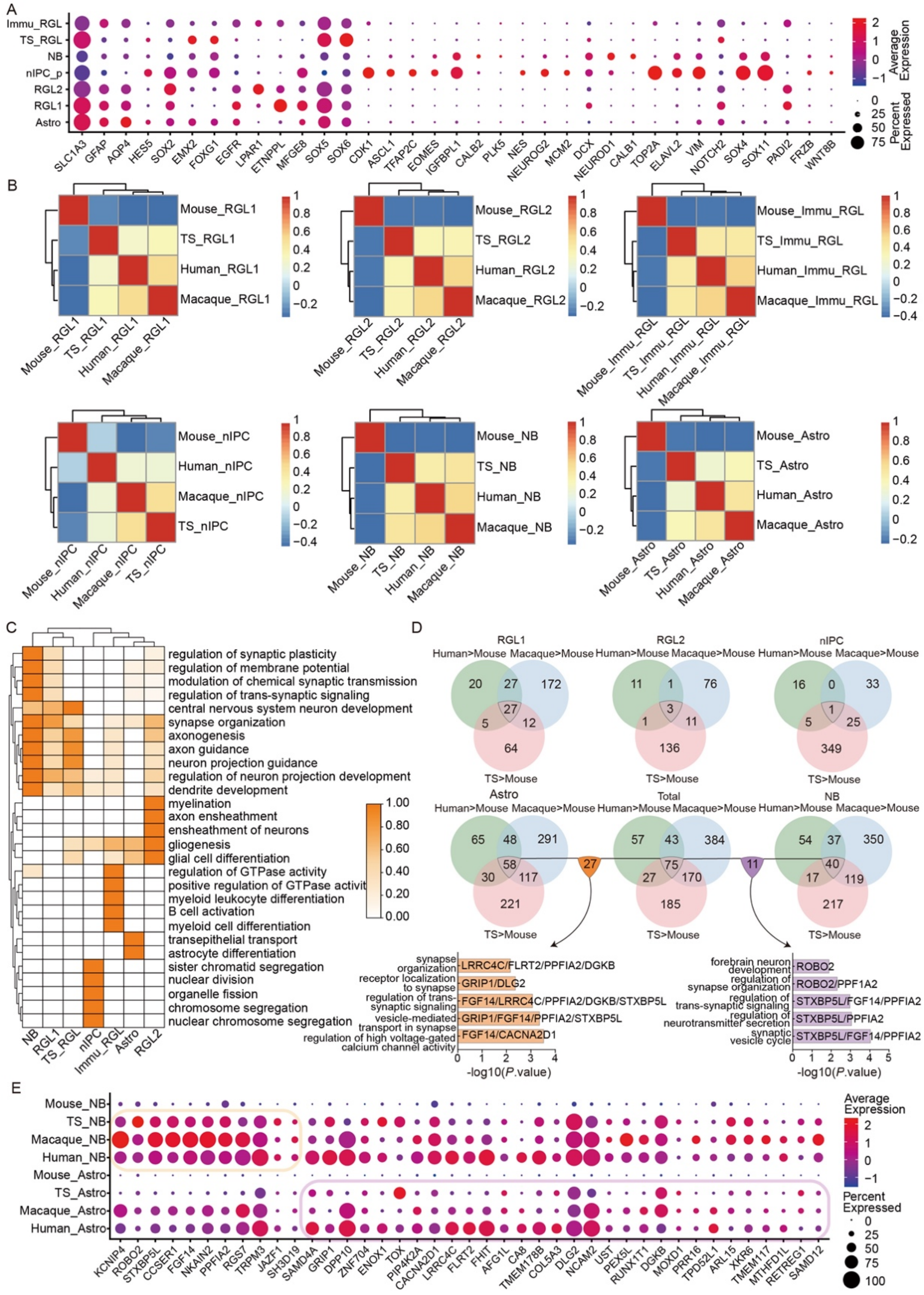

Supplementary Fig.13 The astrocyte and NSC was identified in the hippocampus from human, macaque, TS and mouse. (A) Bubble dot plots of the canonical marker genes of astrocyte, RGL and NB. The size of the dot indicates expression percentage and the darkness of the color indicates average expression. (B) Cell type similarity among human, macaques, TS and mice. The color of each cell represents the alignment score between cells. The larger value (red in the

heatmap) indicates a better alignment. Colorbar indicates the alignment score. (C) Heatmap of enriched GO terms in NSC and astrocyte subclusters. (D) Venn showing the DEGs between mouse and other species (adjusted *P*-value < 0.05, |logFC| > 0.25) in different cell types in hippocampus. (E) Bubble dot plots of the TS and primate special marker genes of astrocyte and NB. The size of the dot indicates expression percentage and the darkness of the color indicates average expression.

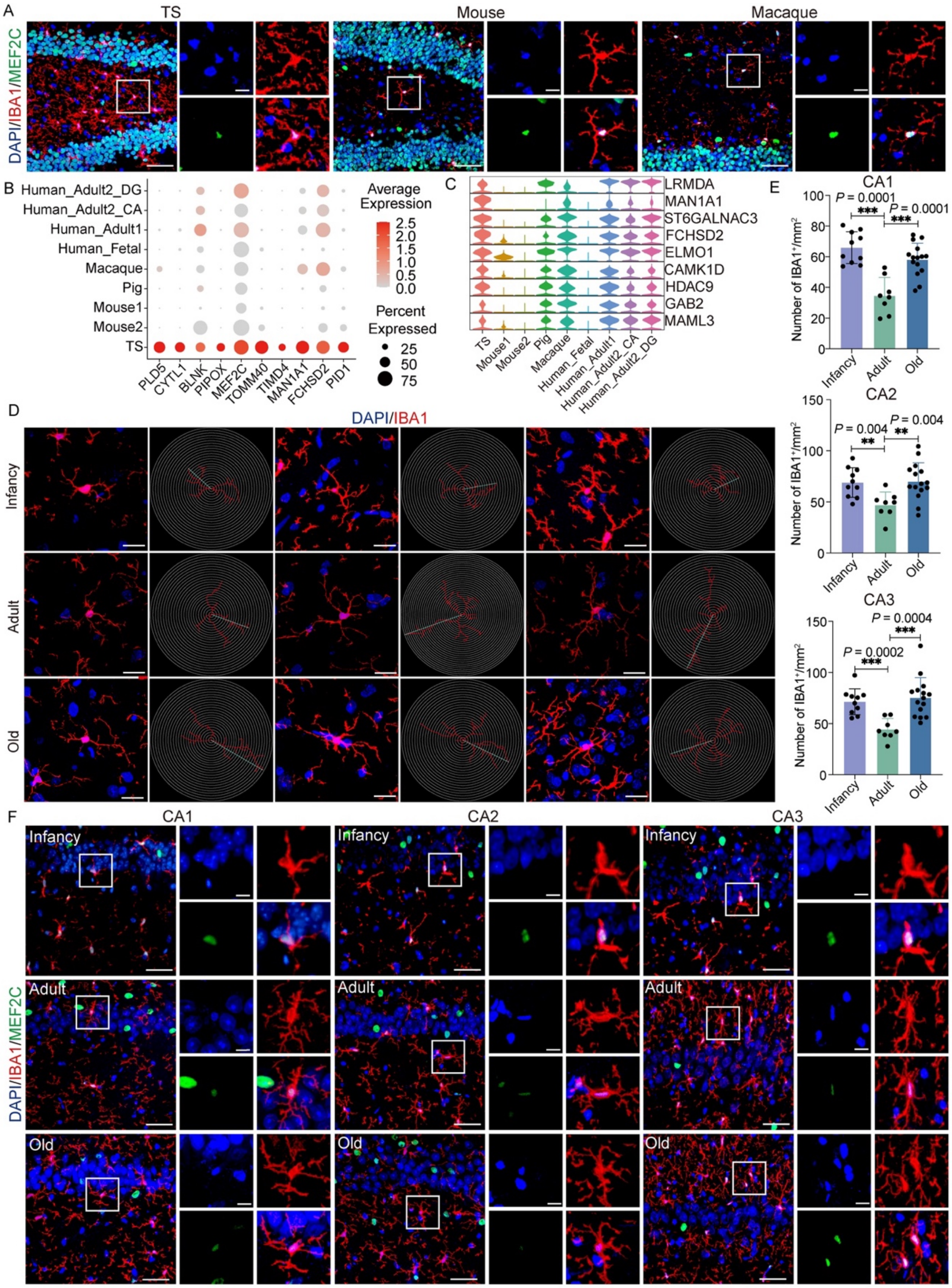

Supplementary Fig.14 Transcriptional and morphological heterogeneity in TS microglia. (A) Representative microscopic fields of *IBA1* (red) / *MEF2C* (green) double-positive cells in the DG of the hippocampus from TS, mouse and macaque. Blue, DAPI. Scale bar: low magnification, 50

μm; high magnification, 10 μm. *n* = 5 per group. (B) Dot plot showing the average expression of TS-special genes of microglia. (C) Violin plot showing the average expression of TS/primate-special genes of microglia. (D) Manual tracking of hippocampus *IBA1*-positive (red) microglia. Sholl analysis: step size = 20 μm. Blue, DAPI. Scale bar: 100 μm. (E) Quantification of *IBA1*-positive cells in the CA1, CA2 and CA3 of the hippocampus from infancy, adult and old TS. *n* = 8-10 per group, one-way ANOVA, $^{**}P < 0.01$, $^{***}P < 0.001$. (F) Representative microscopic fields of *IBA1* (red) / *MEF2C* (green) double-positive cells in the CA regions of the hippocampus from infancy, adult and old TS. Blue, DAPI. Scale bar: low magnification, 50 μm; high magnification, 10 μm. White rectangular boxes represent the focused areas and white arrowhead indicates the cells of interest.

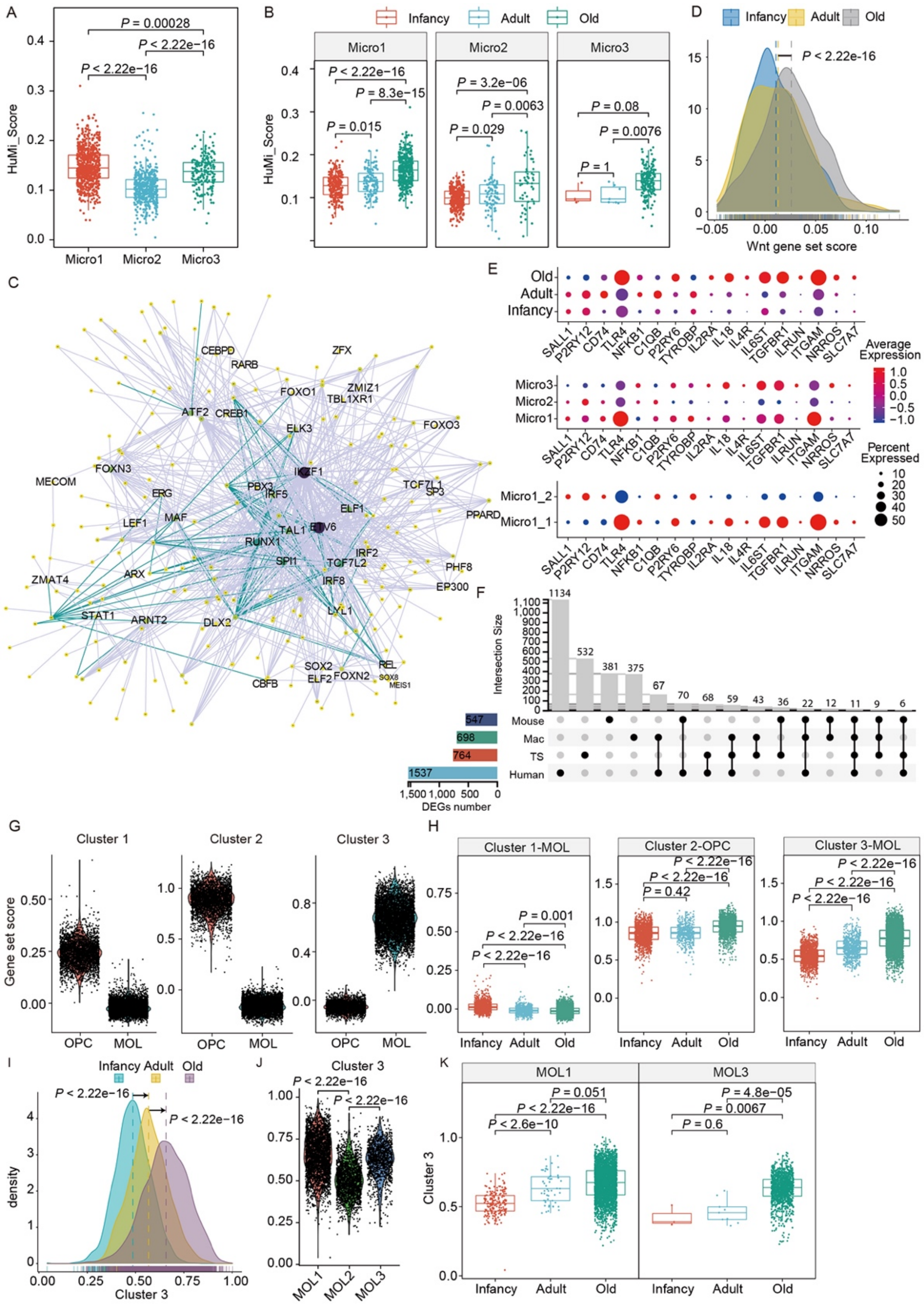

Supplementary Fig.15 Transcriptional heterogeneity of cells derived from TS microglia and MOL. (A) Boxplot showing gene set scores of HuMi_aged gene in microglia subclusters (two-sided Wilcoxon rank-sum test). (B) Boxplot showing increasing gene set scores in microglia subclusters with age (two-sided Wilcoxon rank-sum test). (C) Network plot showing transcriptional regulators of microglia aging-related DEG. (D) Density plot showing gene set scores of genes related to canonical Wnt signaling pathway in microglia of the TS hippocampus

from infancy, adult and old groups. (E) Top, dot plot showing the average expression of activated and non-activated genes of microglia from infancy, adult and old groups. Middle, dot plot showing the average expression of activated and non-activated genes of microglia from microglia subclusters. Below, dot plot showing the average expression of activated and non-activated genes of microglia from micro1_1 and micro1_2. (F) Cross-species comparison of DEGs (FDR-adjusted P-value < 0.05, $|\log_2 FC| > 0$) in the hippocampus microglia. Left bar plot: Number of DEGs identified in each species. Human has the highest number of DEGs (1537), followed by tree shrew (764), macaque (698), and mouse (547), indicating species-specific transcriptional changes. Right upset plot: Intersection analysis of DEGs across the four species (Human, TS, Macaque, and Mouse). The bar heights represent the size of DEG intersections across species. The largest group (1134 DEGs) is unique to mouse, while smaller subsets are shared across multiple species, highlighting conserved and species-specific transcriptional responses. (G) Violin plots showing gene set scores of indicated clusters in OPC and MOL. (H) Box plots showing gene set scores of indicated clusters in different cell type from infancy, adult and old groups (two-sided Wilcoxon rank-sum test). (I) Density plot showing gene set scores of clusters 3 in MOL of the TS hippocampus from infancy, adult and old groups. (J) Violin plots showing gene set scores of clusters 3 in MOL subclusters (two-sided Wilcoxon rank-sum test). (K) Box plots showing gene set scores of clusters 3 in MOL subclusters from infancy, adult and old groups (two-sided Wilcoxon rank-sum test).

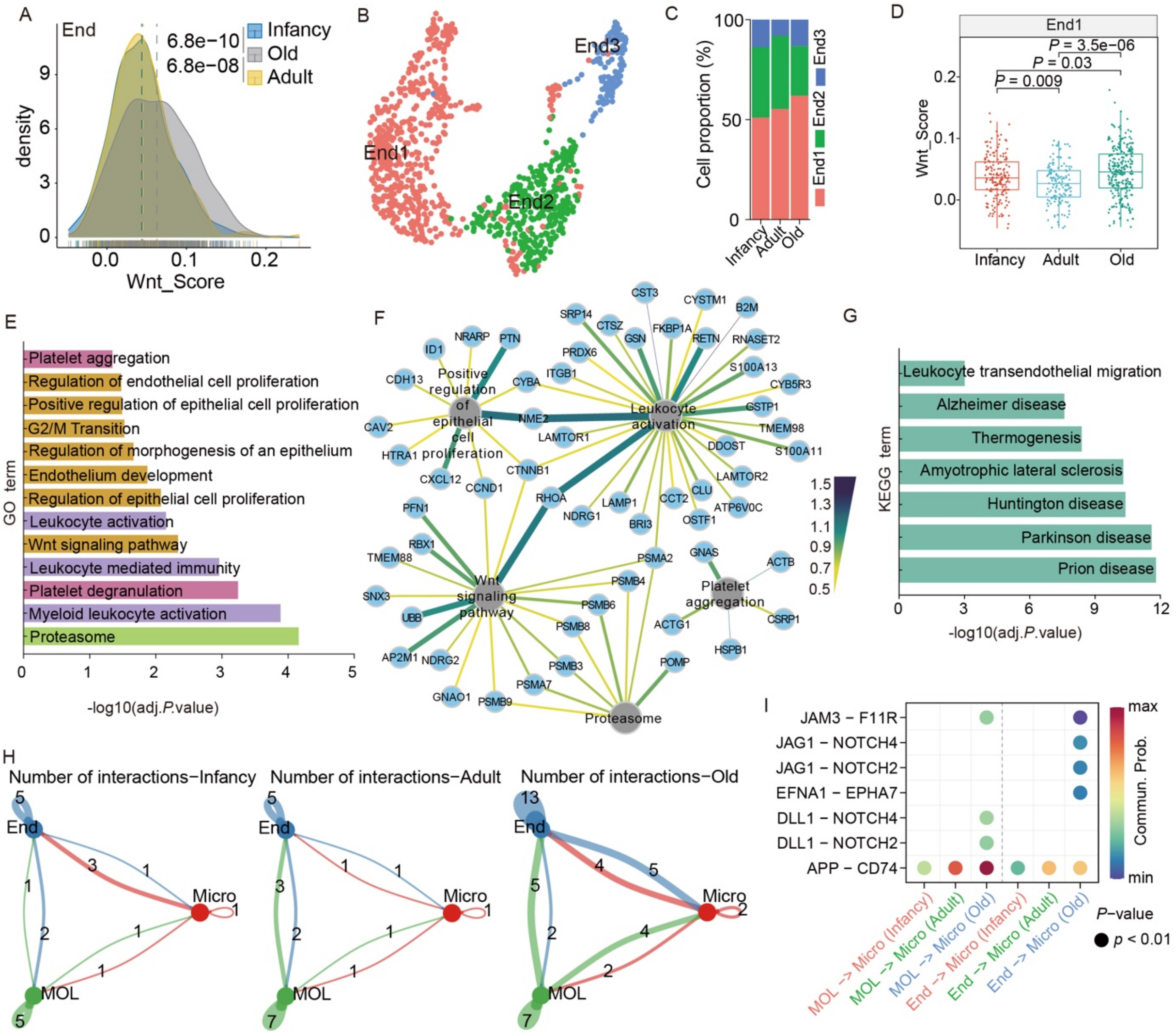

Supplementary Fig.16 Identification of the End populations. (A) Density plot shows gene set scores of genes related to canonical Wnt signaling pathway in End of the TS hippocampus from infancy, adult and old groups. (B) Left, Visualization of End subclusters using UMAP. (C) Bar plot shows the proportions of End subclusters in the hippocampus from infancy, adult and old groups. (D) Density plot shows gene set scores of genes related to canonical Wnt signaling pathway in End1 of the TS hippocampus from infancy, adult and old groups. (E) Enriched GO terms for End aging-related gene from adult and old groups. (F) Network plot of enriched GO terms in End. Nodes for genes were colored by log2fold change derived from analysis of DEGs. (G) Enriched KEGG terms for End aging-related gene from adult and old groups. (H) Network plot shows the ligand–receptor interaction number between each of the two different cell types from infancy, adult and old groups. (I) Dot plot shows the ligand–receptor interaction strength between each of the two different cell types from infancy, adult and old groups.

Supplementary Tables

Supplementary Table 1
The number of cells for clusters and cell types.

Supplementary Table 2
Marker genes for all cell types.

Supplementary Table 3
Marker genes of different cell types in TS hippocampus.

Supplementary Table 4
GSEA of different cell types in TS hippocampus.

Supplementary Table 5
GSEA for TS cell clusters.

Supplementary Table 6
Transcriptional regulatory network governing cell-specific DEGs.

Supplementary Table 7
Information of snRNA-seq datasets used in this study.

Supplementary Table 8
Spearman correlation analysis of snRNA-seq dataset cross species.

Supplementary Table 9
CellChat analysis for human, macaques, TS and mice.

Supplementary Table 10
Disease-related gene sets used in this study.

Supplementary Table 11
Development-related DEGs for each cell type (infancy v.s. adult, adjust.p.value < 0.05, logFC > 0.25).

Supplementary Table 12
Aging-related DEGs for each cell type (adult v.s. old, adjust.p.value < 0.05, logFC > 0.25).

Supplementary Table 13
CellChat analysis for the cell type of hippocampus from infancy, adult and old TS.

Supplementary Table 14
Marker genes of microglia subtype in TS hippocampus

Supplementary Table 15

The antibody information used in this study
